# Inhibition of mitochondrial complex II in neuronal cells triggers unique pathways culminating in autophagy with implications for neurodegeneration

**DOI:** 10.1038/s41598-020-79339-2

**Published:** 2021-01-15

**Authors:** Sathyanarayanan Ranganayaki, Neema Jamshidi, Mohamad Aiyaz, Santhosh-Kumar Rashmi, Narayanappa Gayathri, Pulleri Kandi Harsha, Balasundaram Padmanabhan, Muchukunte Mukunda Srinivas Bharath

**Affiliations:** 1 Department of Neurochemistry, National Institute of Mental Health and Neurosciences (NIMHANS), No. 2900, Hosur Road, Bangalore, Karnataka 560029 India; 2 Department of Radiological Sciences, Ronald Reagan UCLA Medical Center, Los Angeles, CA 90095 USA; 3 Genotypic Technology Pvt. Ltd., 2/13, Balaji Complex, 80 feet Road, RMV 2nd Stage, Bangalore, Karnataka 560094 India; 4 Department of Neuropathology, NIMHANS, No. 2900, Hosur Road, Bangalore, Karnataka 560029 India; 5 Department of Neurovirology, NIMHANS, No. 2900, Hosur Road, Bangalore, Karnataka 560029 India; 6 Department of Biophysics, NIMHANS, No. 2900, Hosur Road, Bangalore, Karnataka 560029 India; 7 Department of Clinical Psychopharmacology and Neurotoxicology, National Institute of Mental Health and Neurosciences (NIMHANS), No. 2900, Hosur Road, Bangalore, Karnataka 560029 India

**Keywords:** Computational biology and bioinformatics, Neuroscience, Systems biology

## Abstract

Mitochondrial dysfunction and neurodegeneration underlie movement disorders such as Parkinson’s disease, Huntington’s disease and Manganism among others. As a corollary, inhibition of mitochondrial complex I (CI) and complex II (CII) by toxins 1-methyl-4-phenylpyridinium (MPP^+^) and 3-nitropropionic acid (3-NPA) respectively, induced degenerative changes noted in such neurodegenerative diseases. We aimed to unravel the down-stream pathways associated with CII inhibition and compared with CI inhibition and the Manganese (Mn) neurotoxicity. Genome-wide transcriptomics of N27 neuronal cells exposed to 3-NPA, compared with MPP^+^ and Mn revealed varied transcriptomic profile. Along with mitochondrial and synaptic pathways, Autophagy was the predominant pathway differentially regulated in the 3-NPA model with implications for neuronal survival. This pathway was unique to 3-NPA, as substantiated by in silico modelling of the three toxins. Morphological and biochemical validation of autophagy markers in the cell model of 3-NPA revealed incomplete autophagy mediated by mechanistic Target of Rapamycin Complex 2 (mTORC2) pathway. Interestingly, Brain Derived Neurotrophic Factor (BDNF), which was elevated in the 3-NPA model could confer neuroprotection against 3-NPA. We propose that, different downstream events are activated upon neurotoxin-dependent CII inhibition compared to other neurotoxins, with implications for movement disorders and regulation of autophagy could potentially offer neuroprotection.

## Introduction

Mitochondrial dysfunction is a common mechanism underlying neurodegeneration associated with movement disorders^1^. Mitochondrial inhibitors induce neurotoxicity in vitro and in vivo and recapitulate many features of neurodegenerative diseases with motor impairment. 1-methyl-4-phenylpyridinium (MPP^+^) is the most widely studied mitochondrial complex I (CI) inhibitor, with implications for Parkinson’s disease. 3-Nitropropionic acid (3-NPA) is a selective inhibitor of mitochondrial complex II (CII) (Succinate Dehydrogenase; SDH)^2^, with implications for Huntington’s disease (HD)^2–5^.

Most studies on neurotoxicity and dysfunction of respiratory complexes focus on CI damage. Of late, CII function, regulation and response to pathophysiological stimuli has emerged to be crucial in bioenergetics and human disease since it is at the crossroads of two essential pathways: Oxidative phosphorylation and Krebs cycle^6^. Genetic mutations and epigenetic changes in CII genes and dysfunction of SDH activity are linked with cancer^7–12^ and mitochondrial disorders including Leigh’s syndrome and optic atrophy^13–15^. CII-mediated reverse electron transfer and ROS generation is linked with ischaemia–reperfusion injury^16^. CII is therefore a promising target for human diseases^17^. In the brain, 3-NPA-mediated CII inhibition induces striatal dysfunction and neurotoxicity that entails mitochondrial dysfunction, metabolic defects and increased ROS^2–5^. We previously showed that 3-NPA neurotoxicity correlated with altered mitochondrial proteome^18^. 3-NPA toxicity is different in cortical and striatal neurons^19^ involving mitochondrial and non-mitochondrial events^20^, thereby highlighting regional specificity in the brain and neuronal cell specificity of neurotoxic mechanisms.

Neurotoxic cell death mechanisms may not be common across toxins. Previously, we compared Idiopathic Parkinson’s disease (iPD) and Manganism, both manifesting as movement disorder with nigrostriatal pathology, by studying neurotoxic models [MPP^+^ and Mn models for iPD and Manganism respectively]^21^. Loss of neuronal processes was observed in the MPP^+^ and not the Mn model. While MPP^+^ lowered the electrophysiological activity of dopaminergic neurons, Mn did not. Transcriptomics revealed several differentially expressed genes to be unique to either models with genes related to neuritogenesis and neuronal proliferation revealing contrasting profile in both. Genome-wide DNA methylation profile was different between both models indicating that iPD and atypical Parkinsonism have divergent neurotoxicological manifestation at the neuronal level.

Similarly, neurotoxin-mediated inhibition of mitochondrial complexes could display varied down-stream events. While CI inhibition induced mitochondria-dependent classical apoptotic mechanisms^3,21–23^, CII inhibition induced relatively lower caspase-3 mechanisms and demonstrated higher dependence on necrotic mechanisms and more rapid cell death^24,25^. Morphological analysis revealed significant differences in ultrastructural changes between MPP^+^ and 3-NPA neurotoxic models^18,21^.

We hypothesized that neuronal death leading to movement disorder induced by different neurotoxins, entail varied mechanisms. Neurotoxic effects following CII inhibition could be different from the events induced by a CI inhibitor and Mn. To address this, we have in the current study, characterized the morphological and molecular changes induced by the CII inhibitor 3-NPA in neuronal cells and compared the same with the alterations induced by CI inhibitor MPP^+^ and the metal toxin Mn, with implications for movement disorder.

## Results

### 3-NPA neurotoxicity is associated with unique transcriptomic profile in N27 neuronal cells compared with other neurotoxins

3-NPA treatment induced dose (0–8 mM) and time (0–48 h) dependent neurotoxicity in N27 cells with LD_50_ of 4 mM at 48 h (Supplementary Figure 1A,B) (consistent with previous studies^26^) and altered cellular morphology including loss of processes (Supplementary Figure 1E). 3-NPA at LD_50_ inhibited Complex II (CII) activity (Supplementary Figure 2A), and induced mitochondrial dysfunction (Supplementary Figure 2B–D) including ultrastructural changes, consistent with the published neurotoxicological features of 3-NPA^2,5,18^. Ultrastructural analysis revealed elongated mitochondria at 4 mM 3-NPA (24 h) (Figure S3Bi), enlarged mitochondria with abnormal, circular cristae (Figure S3Ci) and loss of cristae (Figure S3Cii), both at 4 mM 3-NPA (48 h). These data indicate mitochondrial dysfunction following 3-NPA treatment. 3-NPA mediated mitochondrial dysfunction was associated with increased cellular hydroperoxides, increased nitric oxide release and decreased [GSH]/[GSSG] ratio (Supplementary Figure 4)^18^.

**Figure 1:**
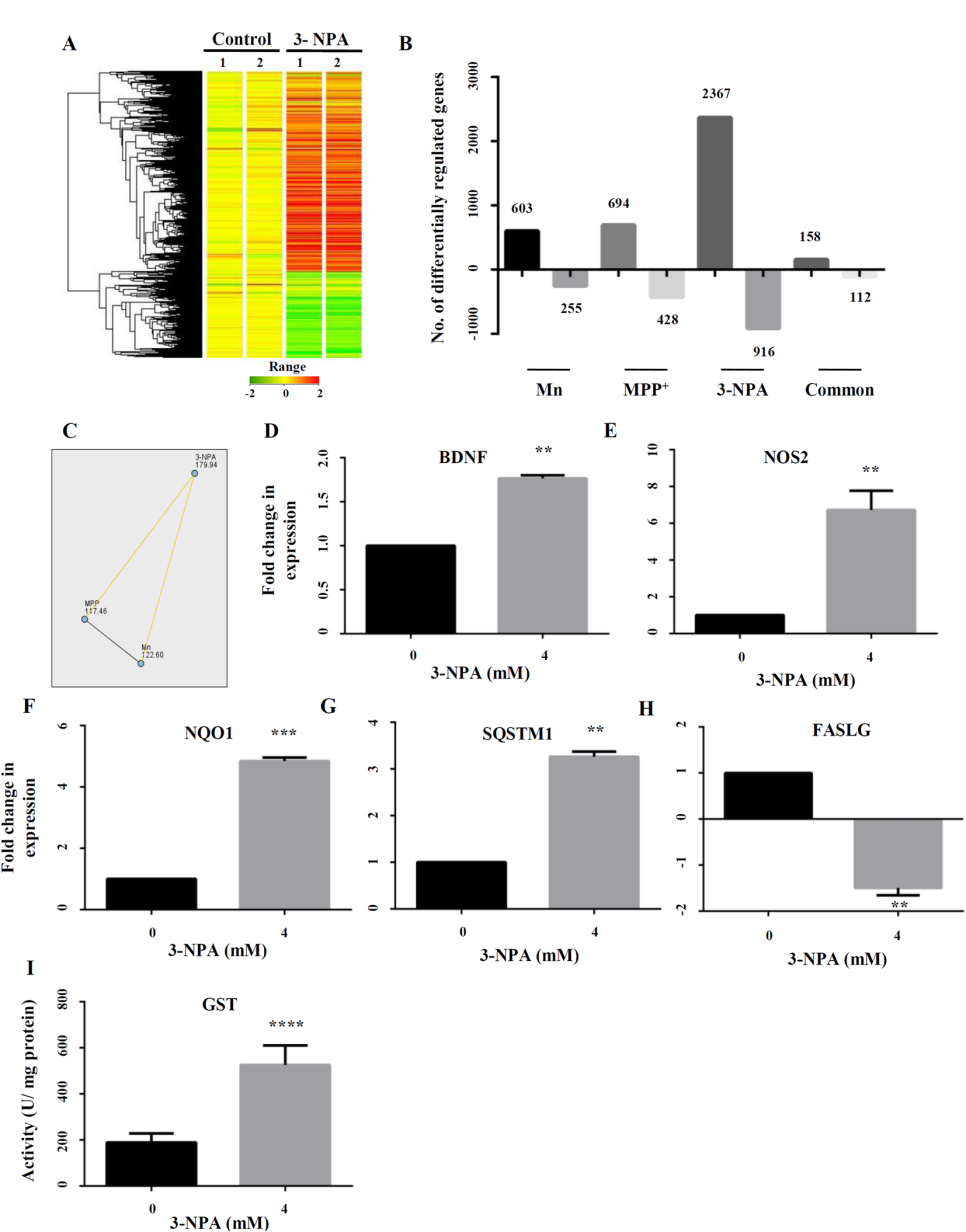
Summary of the transcriptomics data along with functional classification and GO analysis of the differentially regulated genes upon 3-NPA/Mn/MPP^+^ treatment. Whole genome microarray of control and treated N27 cells (Mn/MPP^+^/3-NPA) following mRNA extraction and hybridization against rat genome array revealed several differentially regulated genes. The heat map of 3-NPA transcriptome vs. control is depicted (**A**) along with the colour coded range of gene expression. The heat map was generated using GeneSpring GX software (Agilent). (**B**) Number of genes differentially regulated in Mn/MPP^+^/3-NPA models along with common genes across the groups. Non-parametric analysis by Kruskal Wallis test followed by post-hoc pair wise comparison with Dunn–Bonferroni correction (**C**) of differentially regulated genes across the three models revealed that the dataset of 3-NPA was different and exclusive from that of Mn (p < 0.001) and MPP^+^ (p < 0.001). To validate the microarray data, 5 genes: BDNF (**D**), NOS2 (**E**), NQO1 (**F**), SQSTM1 (**G**) and FASLG (**H**) were tested, whose expression was consistent with the microarray data. Total GST activity was higher in 3-NPA treatment vs. control (I), consistent with the microarray data (n = 3 trials per experiment; **p < 0.01, ***p < 0.001, ****p < 0.0001).

To investigate whether CII inhibition altered the gene expression profile, whole genome transcriptomics was carried out in 3-NPA treated N27 cells at LD_50_ (vs. control) by cDNA microarray analysis (Fig. 1A). The raw intensities of the microarray signals were normalized following rigorous quality checks and background correction. High data quality was depicted by the MVA graphs and box plots, while principal component analysis (PCA) ascertained the quality of grouping of the data of the biological replicates (Supplementary Figure 5). Of ~ 30,000 genes [> 1.75 and < 0.55-fold vs. control as threshold for up and down-regulation respectively] that were obtained, analysis was carried out on genes within the threshold limit and were filtered with p-value ≤ 0.05, followed by False Discovery Rate (FDR) analysis (p < 0.1) to obtain the number of up-regulated and down-regulated genes (Fig. 1B and Supplementary Tables 2, 3).

To analyse whether the altered transcriptome was specific to CII inhibition, we compared this data with the transcriptome profile of N27 cells exposed to the complex I (CI) inhibitor MPP^+^ and the metal toxin Mn (Supplementary Figure 1C,D and Fig. 1B) at LD_50_, as descried earlier by us^21^ (to avoid variability due to cell type and ensure easier comparison, we employed N27 cells for all toxins treated at their respective LD_50_, although this concentration varies across the three toxins).

Among the differentially expressed genes, 2367 genes were exclusively up-regulated in the 3-NPA model, 694 in MPP^+^ and 603 in Mn (vs. control), while 158 were common to all. Among the under-expressed genes, 916 were down regulated exclusively in the 3-NPA model, 428 in MPP^+^ and 255 in Mn (vs. control), while 112 genes were common to all (Fig. 1B). Pair-wise comparison of the transcriptome revealed significant differences between 3-NPA and the other two toxins (Fig. 1C). Non-parametric analysis by Kruskal–Wallis test highlighted statistically significant difference in mean ranks of fold change among the three toxins (x^2^ = 34.4, p < 0.001). Post-hoc pair-wise comparison with Dunn–Bonferroni correction revealed that CII-inhibition dependent transcriptomic changes showed significantly higher fold change compared to Mn (p < 0.001) and MPP^+^ (p < 0.001) (Fig. 1C). These data highlight significant genome-wide transcriptomic differences following CII-inhibition, compared with CI-inhibition and Mn neurotoxicity.

Five of the differentially expressed genes (4 up-regulated and 1 down-regulated) in the 3-NPA model were randomly selected for validation of the microarray data, by qRT-PCR. Brain Derived Neurotrphic Factor (BDNF)^27^, up-regulated by 1.8-fold in microarray data showed ~ twofold increase in the RT-PCR experiment (Fig. 1D). Nitric Oxide Synthase 2 (NOS2), up-regulated by 2.8-fold in the microarray experiment showed ~ 6.5-fold increase in qRT-PCR (Fig. 1E). Similarly, NAD(P)H Quinone Dehydrogenase 1 (NQO1)^28^, up-regulated by 2.64-fold in the microarray experiment showed ~ fivefold increase by qRT-PCR (Fig. 1F). Sequesterome 1 (SQSTM1), a key autophagic protein^29^ up-regulated by twofold in the microarray experiment showed ~ threefold increase by qRT-PCR (Fig. 1G). On the other hand, FAS Ligand (FASLG)^30^, down-regulated by 2.8-fold in the microarray data showed ~ 1.8-fold down-regulation by qRT-PCR (Fig. 1H). Several isoforms of glutathione-S-transferase (GST)^31^ showed consistent up-regulation in the microarray data (Table 1), which was validated by elevated enzyme activity of GST in the 3-NPA treatment (vs. control) (Fig. 1I).

**Table 1:** List of various subunits and isoforms of glutathione-s-transferase (GST) upregulated in 3-NPA treated N27 cells.

Sl. no	Gene symbol	Gene name	Fold change (3NPA/control)
1	Gsta2	Glutathione S-transferase alpha 2	3.84
2	Gsta3	Glutathione S-transferase A3	15.70
3	Gsta4	Glutathione S-transferase alpha 4	3.29
4	Gsta5	Glutathione S-transferase Yc2 subunit	5.12
5	Gsta5	Glutathione S-transferase alpha 5	6.00
6	Gstm1	Glutathione S-transferase mu 1	1.84
7	Gstm7	Glutathione S-transferase, mu 7	2.16
8	Gstp1	Glutathione S-transferase pi 1	2.03
9	Mgst2	Microsomal glutathione S-transferase 2	2.11
10	Mgst3	Microsomal glutathione S-transferase 3	7.55

The gene symbol and gene name of different GST subunits/isoforms along with the fold change in expression (vs. control) noted in the transcriptomics experiment are provided.

### Differences in functional pathways induced by 3-NPA, Mn and MPP^+^: focus on autophagy-related genes

Functional analysis revealed global differences among Mn, MPP^+^ and 3-NPA models. K-mean clustering carried out to partition genes with a particular level of expression as apriori set of six clusters indicated different clustering pattern among the three models (Supplementary Figure 6A–C) with cluster CL6, showing maximum number of genes in Mn and CL3 in both MPP^+^ and 3-NPA. The number of genes significantly differentially expressed among the groups as a fraction of variability is plotted in the Pareto graph (Supplementary Figure 6D).

**Figure 2:**
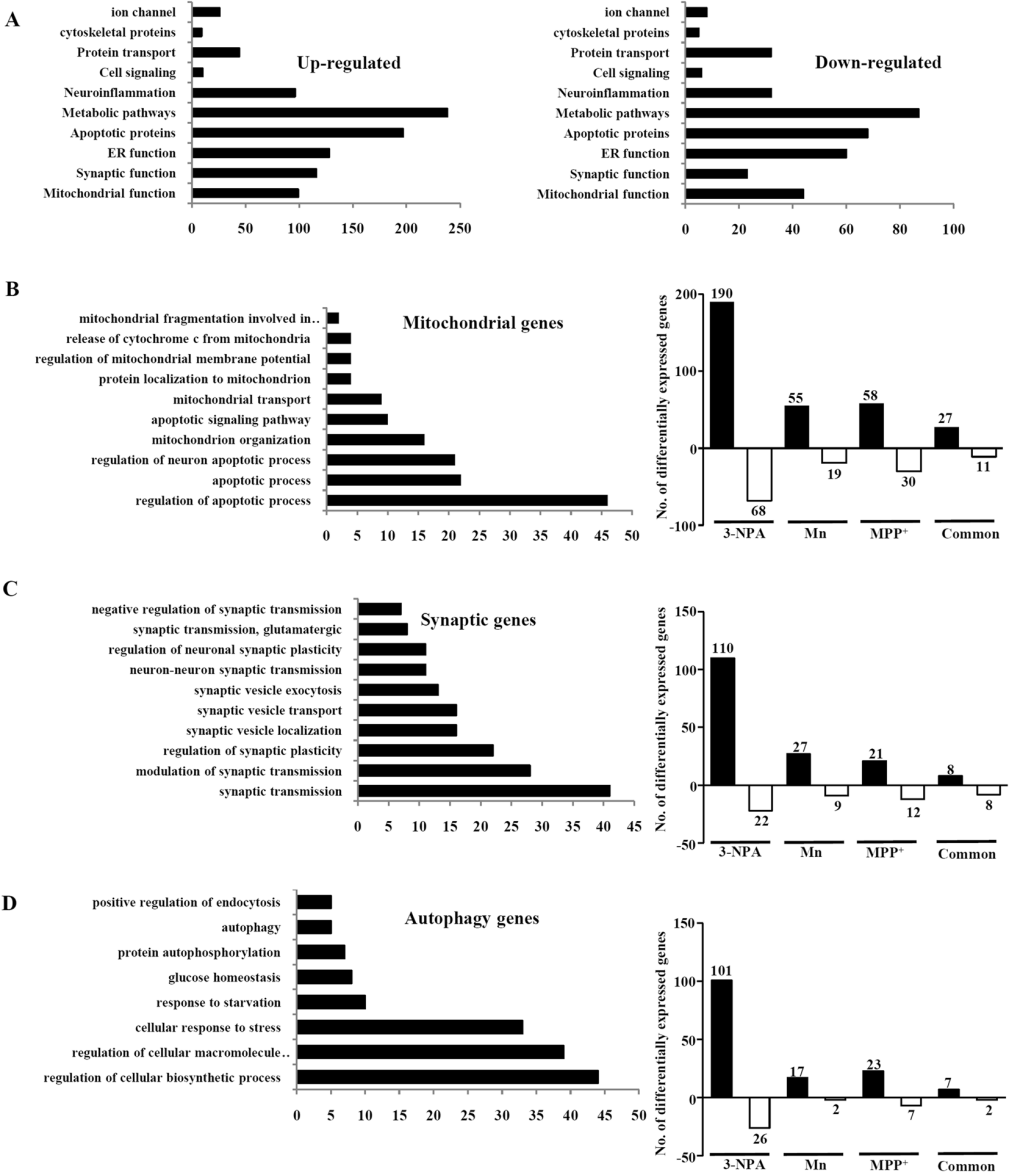
Functional classification and GO analysis of the differentially regulated genes upon Mn/MPP^+^/3-NPA treatment. (**A**) Functional classification of all the up- and down-regulated genes in the 3-NPA model. Functional classification and the number of differentially regulated genes among three major pathways—mitochondrial (**B**), synaptic (**C**) and autophagic (**D**) are shown.

Gene ontology analysis of the up and down-regulated genes showed varied biological processes associated with 3-NPA (Supplementary Figure 7). The up-regulated genes were part of several pathways but significantly clustered into 3 groups: mitochondrial, synaptic and autophagic. Down-regulated genes predominantly clustered as mitochondrial, metabolic and apoptotic functional groups (Fig. 2A–D). Comparison of the differentially expressed mitochondrial genes revealed 88 in MPP^+^ (58 genes up-regulated; 30 genes down-regulated), 74 in Mn (55 up-regulated; 19 down-regulated) and 258 in 3-NPA (190 up-regulated; 68 down-regulated) that were differentially regulated and unique to each group, while 38 genes (27 up-regulated and 11 down-regulated) were common (Fig. 2B). Regarding synaptic genes, 33 in MPP^+^ (21 genes up-regulated; 12 genes down-regulated), 36 in Mn (27 up-regulated; 9 down-regulated) and 132 genes in 3-NPA (110 up-regulated and 22 down-regulated) treatment, while 16 genes (8 up-regulated and 8 down-regulated) were common across the 3 groups (Fig. 2C). Regarding autophagy genes, 30 in MPP^+^ (23 genes up-regulated and 7 genes down-regulated), 19 in Mn (17 up-regulated and 2 down-regulated), 127 in 3-NPA (101 up-regulated and 26 down-regulated) were differentially regulated and unique to each group, while only 9 (7 up-regulated and 2 down-regulated) were common (Fig. 2D). Overall, the number of autophagy genes common across the 3 toxins was the lowest, compared with synaptic and mitochondrial genes. Most of the autophagy genes were altered only in 3-NPA and not in Mn/MPP^+^ models (Table 2) highlighting the importance of autophagy in 3-NPA neurotoxicity. To substantiate this and understand the metabolic changes across the three toxins, we built in silico models and characterized the differences.

**Table 2:** List of autophagic and apoptotic genes differentially regulated across Mn/MPP^+^/3-NPA treatment.

Gene symbol	Gene name	Fold change (vs. control)		
		3-NPA	Mn	MPP^+^
**Autophagy**				
Atg3	Autophagy related 3	0.45	UC	UC
Atg16l1	Autophagy related 16-like 1 (*S. cerevisiae*)	3.03	UC	UC
Unc45b	unc-45 homolog B (*C. elegans*)	3.08	UC	UC
Unc5c	unc-5 homolog C (*C. elegans*)	1.94	UC	UC
Unc5cl	unc-5 homolog C (*C. elegans*)-like	7.62	UC	UC
Uncx	UNC homeobox	2.48	UC	UC
Ulk2	Unc-51 like kinase 2 (*C. elegans*)	0.42	UC	UC
Unc50	unc-50 homolog (*C. elegans*)	0.51	UC	UC
Gabarapl1	GABA(A) receptor-associated protein like 1	2.52	UC	UC
Sqstm1	Sequestosome 1	1.98	UC	UC
Traf3ip3	TRAF3 interacting protein 3	2.26	UC	UC
Tnik	TRAF2 and NCK interacting kinase	3.62	UC	UC
Traf6	TNF receptor-associated factor 6, E3 ubiquitin protein ligase	2.10	UC	UC
Mdm2	p53 E3 ubiquitin protein ligase	3.11	UC	UC
Mdm4	Mdm4 p53 binding protein homolog (mouse)	2.25	UC	UC
Dapk1	Death associated protein kinase 1	2.69	UC	UC
Dapk3	Death-associated protein kinase 3	0.54	UC	UC
Map1b	Microtubule-associated protein 1B	3.24	UC	UC
Prkaa1	Protein kinase, AMP-activated, alpha 1 catalytic subunit	1.89	UC	UC
Prkaa2	Protein kinase, AMP-activated, alpha 2 catalytic subunit	2.17	UC	UC
Prkab2	Protein kinase, AMP-activated, beta 2 non-catalytic subunit	3.48	UC	UC
Prkag2	Protein kinase, AMP-activated, gamma 2 non-catalytic subunit	10.25	UC	UC
Cnksr1	Connector enhancer of kinase suppressor of Ras 1	2.23	UC	UC
Pih1d1	PIH1 domain containing 1	0.46	UC	UC
Pih1d2	PIH1 domain containing 2	0.56	UC	UC
Ctss	Cathepsin S	2.43	UC	UC
Ctsw	Cathepsin W	5.41	UC	UC
Xdh	Xanthine dehydrogenase	4.88	UC	UC
Dnm1l	dynamin 1-like	0.53	UC	UC
Parp11	Poly (ADP-ribose) polymerase family, member 11	3.94	UC	UC
Tiparp	TCDD-inducible poly(ADP-ribose) polymerase	1.90	UC	UC
Ppara	Peroxisome proliferator activated receptor alpha	1.85	UC	UC
Ppargc1b	Peroxisome proliferator-activated receptor gamma, coactivator 1 beta	2.01	UC	UC
Rictor	RPTOR independent companion of MTOR, complex 2	2.01	UC	UC
Deptor	DEP domain containing MTOR-interacting protein	2.41	UC	UC
Sgk2	Serum/glucocorticoid regulated kinase 2	2.88	UC	UC
Eif4ebp1	Eukaryotic translation initiation factor 4E binding protein 1	1.81	UC	UC
Perp	PERP, TP53 apoptosis effector	1.99	UC	UC
Tp53i11	Tumor protein p53 inducible protein 11	1.80	UC	UC
Tp53rk	TP53 regulating kinase	1.82	UC	UC
Tp53inp2	Tumor protein p53 inducible nuclear protein 2	0.34	UC	UC
Tprkb	Tp53rk binding protein	0.52	UC	UC
Sesn2	sestrin 2	3.14	UC	UC
**Genes activated by AKT**				
Usp9x	Ubiquitin specific peptidase 9, X-linked	0.52	UC	UC
Plk5	Polo-like kinase 5	3.83	UC	UC
Mdm2	p53 E3 ubiquitin protein ligase	3.11	UC	UC
Chuk	Conserved helix-loop-helix ubiquitous kinase	1.82	UC	UC
Stim1	Stromal interaction molecule 1	2.06	UC	UC
**FOXO family genes**				
Foxa1	Forkhead box A1	2.00	UC	UC
Foxe1	Forkhead box E1 (thyroid transcription factor 2)	2.76	UC	UC
Foxj2	Forkhead box J2	1.79	UC	UC
Foxk1	Forkhead box K1	2.07	UC	UC
Foxo6	Forkhead box O6	2.27	UC	UC
Foxc1	Forkhead box C1	0.41	UC	UC
Foxq1	Forkhead box Q1	0.33	UC	UC
**RAB family genes**				
Rab25	RAB25, member RAS oncogene family	0.03	UC	UC
Rab2a	RAB2A, member RAS oncogene family	0.57	UC	UC
Rab32	RAB32, member RAS oncogene family	0.38	UC	UC
Rabl5	RAB, member RAS oncogene family-like 5	0.40	UC	UC
Rab11fip1	RAB11 family interacting protein 1 (class I)	2.85	UC	UC
Rab20	RAB20, member RAS oncogene family	2.09	UC	UC
Rab30	RAB30, member RAS oncogene family	2.15	UC	UC
Rab3a	RAB3A, member RAS oncogene family	3.10	UC	UC
Rab3il1	RAB3A interacting protein (rabin3)-like 1	3.11	UC	UC
Rab6b	RAB6B, member RAS oncogene family	1.98	UC	UC
Rab7b	Rab7b, member RAS oncogene family	2.02	UC	UC
Rab9b	RAB9B, member RAS oncogene family	2.53	UC	UC
**RAS family genes**				
Radil	Ras association and DIL domains	2.80	UC	UC
Rasa3	RAS p21 protein activator 3	1.81	UC	UC
Rasa4	RAS p21 protein activator 4	2.16	UC	UC
Rasa4	RAS p21 protein activator 4	2.20	UC	UC
Rasal1	RAS protein activator like 1 (GAP1 like)	2.42	UC	UC
Rasal3	RAS protein activator like 3	2.93	UC	UC
Rasgrp2	RAS guanyl releasing protein 2 (calcium and DAG-regulated)	2.15	UC	UC
Rasl10a	RAS-like, family 10, member A	2.13	UC	UC
Rassf5	Ras association (RalGDS/AF-6) domain family member 5	4.39	UC	UC
Rassf9	Ras association (RalGDS/AF-6) domain family (N-terminal) member 9	2.38	UC	UC
Rem2	RAS (RAD and GEM) like GTP binding 2	2.37	UC	UC
Rerg	RAS-like, estrogen-regulated, growth-inhibitor	1.77	UC	UC
Rhebl1	Ras homolog enriched in brain like 1	2.06	UC	UC
Rhov	ras homolog family member V	3.85	UC	UC
RragB	Ras-related GTP binding B	2.78	UC	UC
Rragd	Ras-related GTP binding D	4.35	UC	UC
Raph1	Ras association (RalGDS/AF-6) and pleckstrin homology domains 1	0.50	UC	UC
Rasl11a	RAS-like family 11 member A	0.26	UC	UC
Rasl12	RAS-like, family 12	0.50	UC	UC
Rasl2-9	RAS-like, family 2, locus 9	0.52	UC	UC
**Growth factors**				
Bdnf	Brain-derived neurotrophic factor	1.80	2.08	UC
Grb7	Growth factor receptor bound protein 7	2.22	UC	UC
Igf2	Insulin-like growth factor 2	4.42	UC	UC
Igf2bp2	Insulin-like growth factor 2 mRNA binding protein 2	2.24	UC	UC
Igf2r	Insulin-like growth factor 2 receptor	1.91	UC	UC
Igfals	Insulin-like growth factor binding protein, acid labile subunit	2.08	UC	UC
Igfbp2	Insulin-like growth factor binding protein 2	2.49	UC	UC
Igfbp5	Insulin-like growth factor binding protein 5	3.71	UC	UC
Igfbpl1	Insulin-like growth factor binding protein-like 1	5.99	UC	UC
**Apoptosis**				
Bbc3	Bcl-2 binding component 3	1.82	UC	UC
Bcl11a	B-cell CLL/lymphoma 11A (zinc finger protein)	1.94	UC	UC
Bcl2	B-cell CLL/lymphoma 2	2.05	UC	UC
Bcl2l10	BCL2-like 10 (apoptosis facilitator)	1.95	UC	UC
Bcl6b	B-cell CLL/lymphoma 6, member B	1.96	UC	UC
Bik	BCL2-interacting killer (apoptosis-inducing)	2.23	UC	UC
Hrk	Harakiri, BCL2 interacting protein (contains only BH3 domain)	13.02	UC	UC
Bcl2l11	BCL2-like 11 (apoptosis facilitator)	0.51	UC	UC
Bcl2l12	BCL2-like 12 (proline rich)	0.46	UC	UC
Bcl3	B-cell CLL/lymphoma 3	0.51	UC	UC
Bnip2	BCL2/adenovirus E1B interacting protein 2	0.46	UC	UC
Aen	Apoptosis enhancing nuclease	3.21	UC	UC
Aifm3	Apoptosis-inducing factor, mitochondrion-associated 3	2.21	UC	UC
Casp4	Caspase 4, apoptosis-related cysteine peptidase	2.23	UC	UC
Faim2	Fas apoptotic inhibitory molecule 2	1.83	UC	UC
Casp9	Caspase 9, apoptosis-related cysteine peptidase	0.42	UC	UC
Cidea	Cell death-inducing DFFA-like effector a	2.28	UC	UC
Pdcd1	Programmed cell death 1	1.84	UC	UC
Dad1	Defender against cell death 1	0.56	UC	UC
Capn8	Calpain 8	11.25	UC	UC
Capns2	Calpain, small subunit 2	2.59	UC	UC

The gene symbol and gene name of different Autophagy and Apoptotic markers along with the fold change in expression (vs. control) noted in the transcriptomics experiment are provided.

UC = unchanged.

### Network interpretation of differentially expressed transcripts: in silico analysis

**Figure 3:**
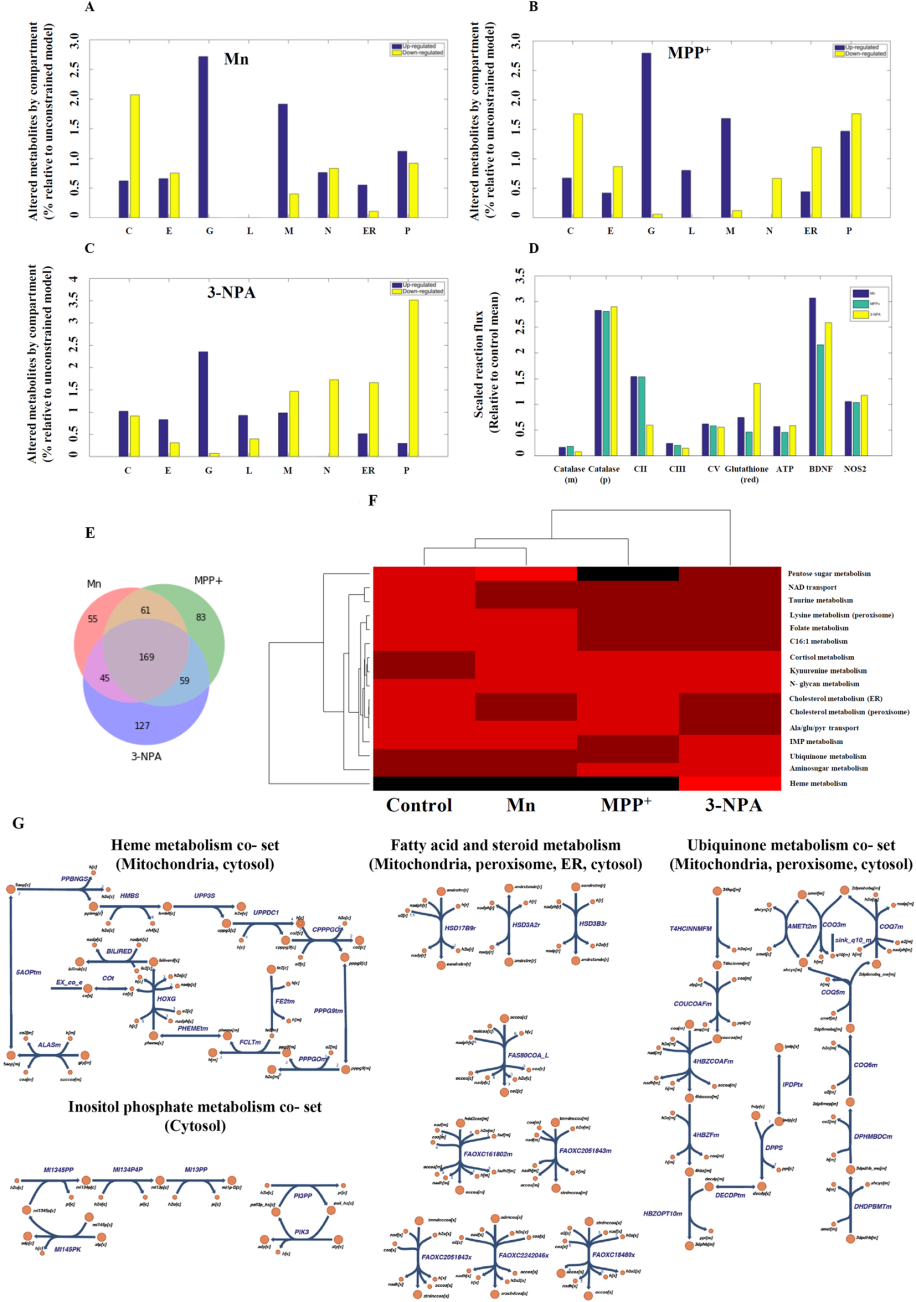
Organellar changes based on metabolic network interpretation of differentially expressed transcripts in toxic cell models vs. control. (**A**–**C**) Mn, MPP^+^, and 3-NPA organelle changes, respectively (C = cytosol; E = extracellular; G = golgi apparatus; L = lysosome; M = mitochondria; N = nucleus; ER = endoplasmic reticulum; P = peroxisome). (**D**) Flux changes for selected enzymes for context specific GeMMs. Mean reaction fluxes for different enzymes and metabolites relative to the corresponding mean flux for untreated N27 cells are shown. All pairwise comparisons are statistically significant for the paired t-test at p < 0.05 following Bonferroni correction except for: Mn vs. MPP^+^ NOS2, and Mn vs 3-NPA NOS2. (m = mitochondria, p = peroxisome, red = reduced state). (**E**) Venn diagram of the reactions based on the differentially expressed genes in each neurotoxic model vs. control (generated using Python (Matplotlib library); URL—https://matplotlib.org/). (**F**) Clustering of reaction co-sets for GeMMs for control, Mn, MPP^+^ and 3-NPA treated cells (generated using MATLAB version 2015b). The colour scale is linear from 0 (Black) to 1 (Bright red). (**G**) Pathway maps of the co-sets and reaction pathways that are differentially active in 3-NPA treated cells and observed to be involved in autophagy (generated using Escher; URL—https://escher.github.io). The heme co-set is responsible for the flux restrictions on CII. Detailed descriptions of the co-sets and constituent reactions are provided in the Supporting information spreadsheets.

Since cellular functions are compartmentalized in organelles, in silico analysis of the differentially expressed transcripts in the context of a metabolic network was carried out focused on variation (enrichment or reduction) of organelle metabolism as assessed by their size. The compartments included the cytosol, endoplasmic reticulum, golgi apparatus, lysosome, mitochondria peroxisome, and extracellular compartment. The identified set of up- and down-regulated genes were used to extract the reaction sub-networks for each of the toxins. The salient observations from this assessment (Fig. 3A–C) reflect a large change in the golgi for all neurotoxins with relatively larger changes in the mitochondria of Mn and MPP^+^ models in comparison to 3-NPA, and lack of lysosomal metabolites in Mn. Altered peroxisomal metabolites are also of note. These findings are consistent with autophagy in 3-NPA treated cells, although the mechanisms by which these changes are manifested are not clear, thus requiring a detailed exploration of the network flux state differences.

### Context-specific GeMM analysis

Context-specific GeMMs were constructed using the transcriptomes, to further characterize the functional metabolic differences among the three models. The different feasible flux states for each of the models were assessed and estimates of the flux states were calculated using FBA, to interrogate the metabolic content and capabilities. Fluxes were compared among the 3 models (vs. control for catalase, mitochondrial and peroxisomal isoforms), CII, CIII and CV, and NOS2, and evaluation of production of ATP, glutathione, and BDNF (Fig. 3D). These fluxes were significantly altered for each of the neurotoxic models and were different among them for almost all of the enzymes (or metabolite demands). These predictions are consistent with experimental data (Fig. 1D,E, Supplementary Figures 2 and 4), which is notable considering that a *qualitative* (i.e. present/absent calls) interpretation of the transcriptomics were used to generate the GeMMs, yet lowered CII, along with altered ATP, glutathione, and BDNF were noted.

Clustering of reactions differentially present in Mn, MPP^+^ and 3-NPA GeMMs according to the canonical or pre-defined sub-systems indicated changes in multiple areas of metabolism, including amino acids, sugars, and fatty acids (Supplementary Figure 8A). For more nuanced and context-specific characterization of the varied metabolic capabilities in the 3 models, cell- and context-specific functional sub-systems were calculated to generate N27 specific pathway sets (co-sets) (see Supplementary information spreadsheets). The N27 co-sets provide a functional interpretation of the different transcriptomic states. At a correlation threshold of 0.95, reaction co-sets were calculated for each model, resulting in 380, 369, 398, and 357 co-sets for the untreated, Mn, MPP^+^ and 3-NPA, respectively. For pathways that involve as least 6 reactions (biochemical transformations or transport reactions), there were 23, 22, 22, and 20 sets for the untreated, Mn, MPP^+^, 3-NPA conditions, respectively (see Supplementary information spreadsheets). As expected, focusing on the largest co-sets, there was a high-degree of overlap between the untreated and toxin treated conditions. However, there were four co-sets that were not present in any of the models but were present in the untreated N27 GeMMs; these involved Keratan sulfate metabolism pathways (involving 3 different co-sets) and Glycogen metabolism. Additionally, the Melanin biosynthesis co-set was not present in MPP^+^ treated cells.

The altered structure of the co-sets in toxin-treated conditions (vs. control) indicates a shift in the utilization of glycosaminoglycan and glycogen. Hierarchical clustering of the mean fluxes for the largest co-sets reveals that the Heme metabolism co-set (Fig. 3F) is the most discriminating pathway between 3-NPA treated cells (vs. other toxins). Further, Uniquinone and IMP Metabolism co-sets were more active in 3-NPA treated cells. Reactions of NAD, taurine, and folate metabolism was decreased (vs. control) in all of the 3 toxin-treated GeMMs, likely reflecting increased oxidative stress. Another clear discriminating pattern is lowered Pentose sugar metabolism (Supplementary Figure 8B) in MPP^+^ treated cells compared to others (although 3-NPA is also decreased relative to untreated and Mn treated).

Comparison of the sampled flux states between each of the GeMMs and control identified 330, 372, and 400 statistically different reaction fluxes for Mn, MPP^+^ and 3-NPA (see Supplementary information spreadsheets; p < 0.05 following Bonferroni corrections and at least a twofold difference in mean flux between treated and untreated cells), respectively. 169 reactions were shared among all toxic models (Fig. 3E). 3-NPA treated cells had the largest number of differentially active reactions comparison to control and Mn/MPP + treated cells (Fig. 3E and Supplementary information spreadsheets). Fatty acid metabolism, particularly the oxidation of long chain fatty acids that involve the peroxisome, were among these reactions (see Supplementary Information).

Taken together, (i) changes seen in the mitochondria/lysosome/peroxisome/golgi, (ii) metabolism of long chain fatty acids (requiring partial peroxisomal metabolism) in 3-NPA model, (iii) changes seen in sugar polymer in all the models compared to control and (iv) alterations in heme and ubiquinone metabolism in the 3-NPA model in particular (Fig. 3G), highlight the possible role of autophagy particularly in the 3-NPA model. Consequently, we tested whether autophagy plays an important role down-stream in the 3-NPA model.

### N27 cells treated with 3-NPA use autophagy as a rescue mechanism

**Figure 4:**
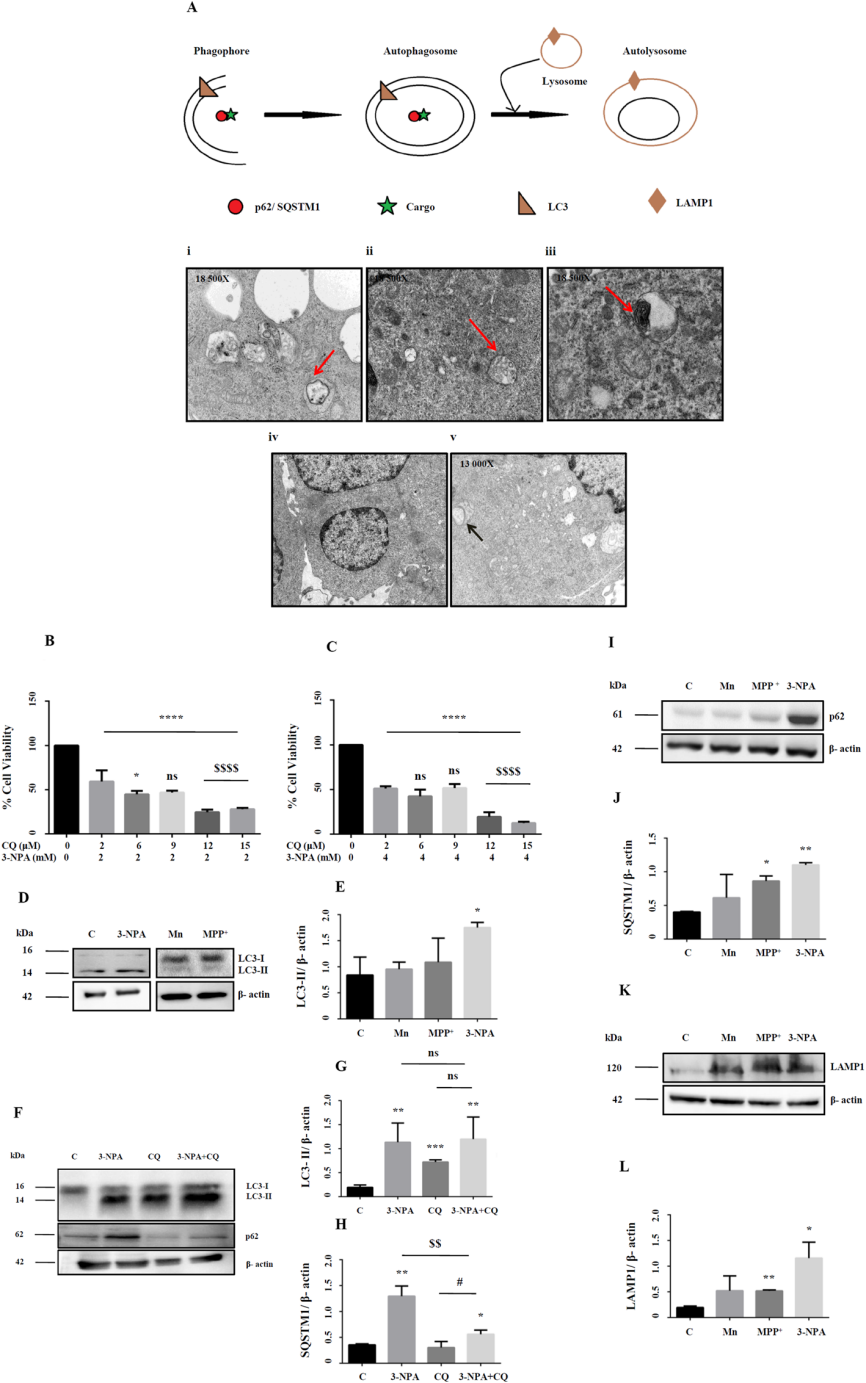
Ultrastructural and biochemical evidences of autophagy in 3-NPA toxicity. Representative electron micrographs of 3-NPA cell model showed evidences of autophagy at various stages starting from formation of autophagosomes (**A**-i) and formation of autolysosomes (**A**-ii). Accumulation of multilamellar vesicles (**A**-iii), a characteristic feature of incomplete autophagy was also observed. Mn treatment did not show discernable morphological changes (**A**-iv). Whereas, MPP^+^ treatment caused cytoplasmic vacuolations along with the presence of abnormal mitochondria (arrow) (n = 3 experiments per group). Arrows represent the described pathological features [schematic was generated using Microsoft Paint (2004)]. (**B**,**C**) Co-treatment of 3-NPA model at 2 and 4 mM with chloroquine (0–15 µM) showed a dose and time dependent increase in cell death as shown by cell viability assay (n = 6 trials per experiment; *p < 0.05, ****p < 0.0001 compared to its respective untreated control; ^$$$$^p  < 0.0001 compared to 3-NPA treated N27 cells at 2 mM and 4 mM; ns-not significant). (**D**–**J**) Standard autophagy markers depicting different stages of autophagy were examined in 3-NPA/Mn/MPP^+^ treated N27 cells. LC3 conversion that represents the autophagosomal mass, was maximum in 3-NPA treatment (**D**,**E**, corresponding complete blots in supplementary Figure 9i (3-NPA) and ii (Mn and MPP^+^)) which was confirmed by CQ cotreatment (**F**,**G**, corresponding complete blot in supplementary Figure 9iv). The LC3 western data for Mn and MPP^+^ is from a blot that is different from the 3-NPA blot (**D**). The β-actin western data for Mn and MPP^+^ is from a blot that is different from the 3-NPA blot (**D**). The status of autophagy upon 3-NPA treatment was further evaluated upon co-treatment with CQ (15 µM), followed by assessment of the levels of LC3-II conversion (**F**,**G**) and p62 (**F**,**H**, corresponding complete blot in supplementary Figure 10v). LC3 converiosn was noted in 3-NPA, CQ and 3NPA + CQ; however, the differences among the three groups was not significant (ns). On the other hand, p62 showed significant over-expression in 3-NPA + CQ treatment both in comparison with control and CQ treatment alone (n = 3 trial per experiment; *p < 0.05, **p < 0.01, ***p < 0.001; compared to untreated control; ^#^p < 0.05, compared to CQ treatment alone; ^$$^p < 0.01, compared to 3-NPA treatment alone). p62/SQSTM1 was also higher in MPP^+^ and 3-NPA treated cells (**I**,**J**, corresponding complete blot in supplementary Figure 10i) indicating possible incomplete autophagy (n = 3 trials per experiment; *p < 0.05, **p < 0.01). LAMP1, the lysosome marker showed significant increase in MPP^+^ and 3-NPA model (**K**,**L**, corresponding complete blot in supplementary Figure 11i). The western data of LC3 in CQ + 3-NPA samples (**F**), p62 (**G**), LAMP1 (**I**) are from 2 different blots. All blots were cut to retain only the region depicting the protein of interest.

Ultrastructural analysis of the 3-NPA treated cells showed evidences of autophagy, including structures of phagophore, autophagosome, autolysosome and typical intracellular multi-lamellar inclusion bodies (Fig. 4A). Such structures were not observed in Mn and MPP^+^ models (Fig. 4A-iv and v). N27 cells treated with 3-NPA and chloroquine, an autophagy inhibitor showed increased cell death compared to 3-NPA treatment alone (Fig. 4B,C). Since CQ treatment increased cell death in the 3-NPA model, suppression of autophagic pathway could exacerbate the neurotoxic effects on 3-NPA leading to increased cell death.

Next, we carried out western blot of the molecular players of autophagy. Analysis of the autophagic flux revealed effective conversion of the microtubule-associated protein light chain 3A, LC3-I to LC3-II species only in 3-NPA and not in Mn/MPP^+^ models (Fig. 4D,E; corresponding full blots in supplementary Figure 9i (3-NPA) and ii (Mn and MPP^+^)). Further, western blot of LC3 in 3-NPA treated cells with and without CQ compared to control indicated relatively higher intensity of LC3-II band (Fig. 4F,G; corresponding full blot in supplementary Figure 9iv). CQ (positive control) independently increased LC3-I conversion compared to control. However, 3-NPA + CQ treatment does not show any further increase in LC3 conversion compared with 3-NPA treatment alone (Fig. 4F,G), indicating that 3-NPA causes incomplete autophagy by inducing possible lysosomal dysfunction rather that inducing formation of new autophagosomes. On the other hand, expression of SQSTM1 or protein 62 (p62) was elevated both in the 3-NPA and MPP^+^ models, with an increasing trend in Mn in the total extract (Fig. 4I,J; corresponding full blot in supplementary Figure 10i), which was also reflected in the soluble fraction (Supplementary Figure 10ii). While CQ treatment alone did not show significant change in p62 expression compared to control, CQ + 3-NPA co-treatment showed significant over expression of p62 (Fig. 4F,H; corresponding full blot in supplementary Figure 10v). Lysosome Associated Membrane Protein 1 (LAMP1), a marker of auto-lysosome showed highest expression in the 3-NPA model (Fig. 4K,L; corresponding full blot in supplementary Figure 11i). These data confirm the role of autophagy exclusively in the 3-NPA model.

### Analysis of the autophagic pathway in the 3-NPA model

**Figure 5:**
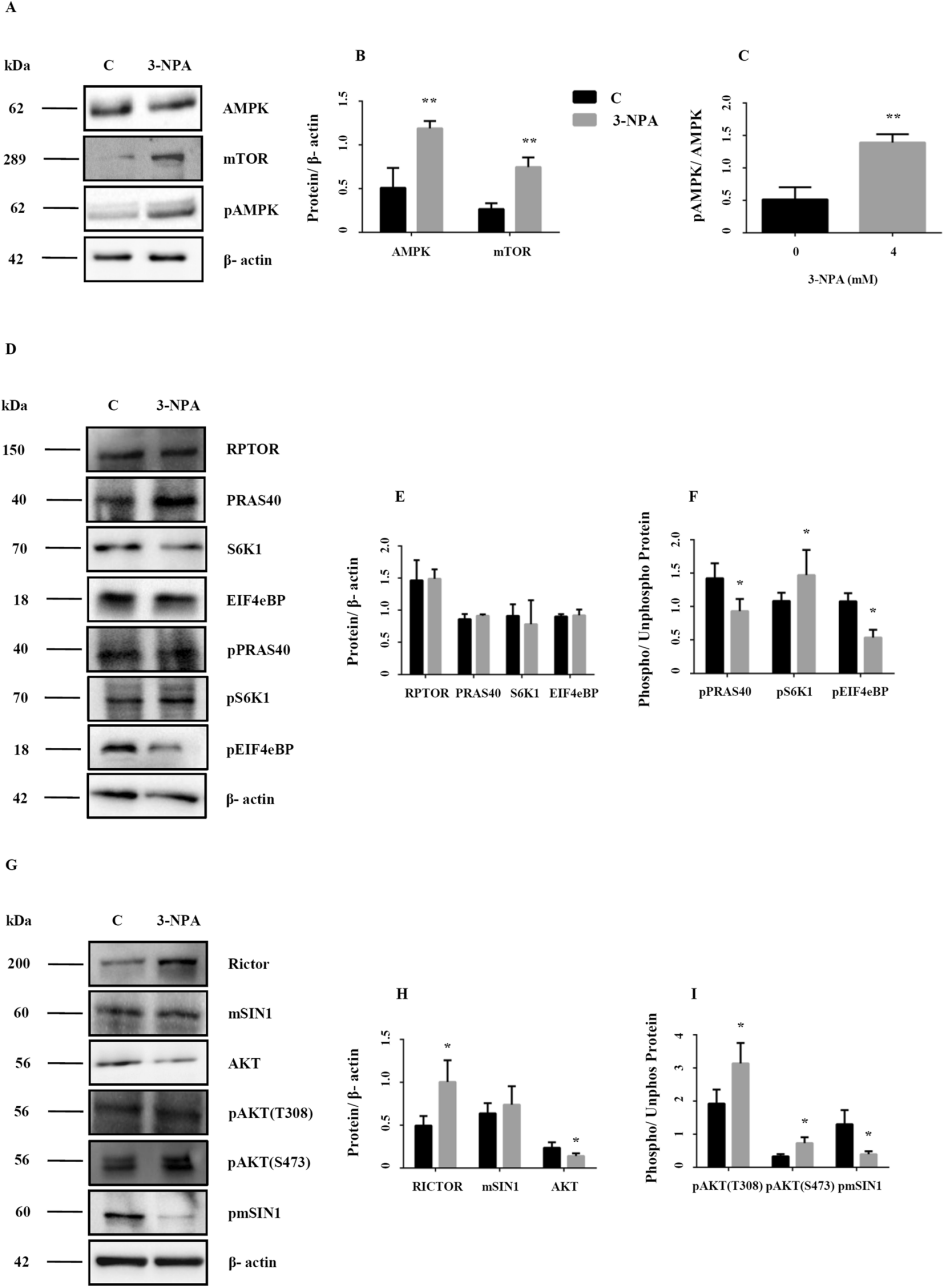
Mechanism and pathways activated in 3-NPA induced autophagy. Consistent with the microarray data, AMPK was up-regulated at the protein level (**A**,**B**, corresponding complete blot in supplementary Figure 11ii) and was activated upon 3-NPA treatment as indicated by increased phosphorylation (**A**,**C**, corresponding complete blot in supplementary Figure 11iii). mTOR itself can function in presence of or independent of AMPK, whose levels were moderately higher in 3-NPA treatment (**A**,**B**, corresponding complete blot in supplementary Figure 11iv) (n = 3 trials per experiment; *p < 0.05, **p < 0.01). Western blotting on total cell extracts revealed that the primary subunit of mTORC1, RAPTOR, was unchanged upon 3-NPA treatment (**D**,**E**, corresponding complete blot in supplementary Figure 12i) and so were the levels of the other regulatory subunit PRAS40 (**D**,**E**, corresponding complete blot in supplementary Figure 12ii) and its downstream targets including S6K1 (**D**,**E**, corresponding complete blot in supplementary Figure 12iii) and EIF4EBP (**D**,**E**, corresponding complete blot in supplementary Figure 12iv). However, the phosphorylation status of PRAS40 (pPRAS40-**D**,**F**, corresponding complete blot in supplementary Figure 13i) and EIF4EBP (pEIF4EBP-**D**,**F**, corresponding complete blot in supplementary Figure 13iv) was significantly lower in 3-NPA treatment along with increased phosphorylation of S6K1 (pS6K1-**D**,**F**, corresponding complete blot in supplementary Figure 13ii). (**G**–**I**) 3-NPA induced changes in mTORC2 subunits and its downstream targets. The primary subunit of mTORC2, RICTOR, was upregulated upon 3-NPA treatment (**G**,**H**, corresponding complete blot in supplementary Figure 14i) unlike the other regulatory subunit mSIN1 (**G**,**H**, corresponding complete blot in supplementary Figure 14ii) and its downstream target AKT (**G**,**H**, corresponding complete blot in supplementary Figure 14iii). However the phosphorylation status of mSIN1 (pSIN1-**G**,**I**, corresponding complete blot in supplementary Figure 15i) was much lower, while phosphorylation of AKT at S473 [pAKT (S473)-**G**,**I**, corresponding complete blot in supplementary Figure 15iii] and T308 (pAKT (T308)-**G**,**I**, corresponding complete blot in supplementary Figure 15ii) was significantly higher in 3-NPA treatment (n = 3 trials per experiment; *p < 0.05, **p < 0.01). The western data for all the proteins in this figure are from independent blots. All blots were cut to retain only the region depicting the protein of interest.

AMP-regulated Protein Kinase (AMPK) is one of the regulators of mechanistic Target of Rapamycin (mTOR), a negative regulator of autophagy^32^. The catalytic subunits of AMPK (PRKAA1 and PRKAA2), were up-regulated in the microarray data (Table 2, Fig. 5A,B; corresponding full blot in supplementary Figure 11ii). Western analysis revealed increased phosphorylation (at Thr172) of AMPK, thus confirming its activation in the 3-NPA model (Fig. 5A,C; corresponding full blot in supplementary Figure 11iii). AMPK mediated cascade could result either in mTOR-dependent or independent autophagy. mTOR exists as mTOR Complex 1 (mTORC1) and mTOR Complex 2 (mTORC2), which have shared and unique subunits^32^.

Microarray data of the 3-NPA model revealed differential regulation of the subunits and down-stream targets of both mTORC1 and mTORC2. mTOR showed unchanged expression (0.8-fold vs. control), while DEP-containing mTOR interacting protein (DEPTOR), a subunit common to both complexes was up-regulated (2.4-fold), and mTOR-associated protein, LST8 homolog (mLST8) (1.3-fold), another component of mTORC1 showed relatively unchanged expression (Table 2).

The unique components of mTORC1 including Regulator Associated Protein of mTORC1 (RPTOR) (0.91-fold) and AKT1 Substrate1 (Proline rich) (PRAS40) (1.1-fold) were relatively unchanged. Similarly, the down-stream targets of mTORC1 including Ribosomal Protein S6 Kinase polypeptide 1 (S6K1) (0.8-fold), UNC-51 like Kinase 1 (ULK1) (0.9-fold) showed relatively unchanged expression. On the other hand, two down-stream targets: Growth factor Receptor Bound protein 10 (GRB10) (0.26-fold) and Eukaryotic translation initiation factor 4e binding protein 1 (Eif4ebp1) (1.8-fold) showed differential regulation in the 3-NPA model (Table 2).

Western blot of mTORC1 components and down-stream targets validated the microarray data. Expression of RPTOR and PRAS was relatively unchanged vs. control (Fig. 5D,E; corresponding full blot in supplementary Figure 12i (RPTOR) and ii (PRAS40)), consistent with microarray data. However, expression of EIF4eBP1 was relatively unchanged, unlike the microarray data, which showed up-regulation (Fig. 5D,E; corresponding full blot in supplementary Figure 12iv). Since mTOR expression was higher on western blot (Fig. 5A,B; corresponding full blot in supplementary Figure 11iv), and to assess whether autophagy is triggered via mTORC1, we tested the phosphorylation status of mTORC1 component and downstream targets. Phosphorylation of PRAS40 at Thr246 and EIF4Ebp1 at T45 was relatively lower vs. control (Fig. 5D,F; corresponding full blot in supplementary Figure 13i (pPRAS40) and iv (pEIF4Ebp1)) indicating that mTORC1 is not activated. However, phosphorylation of S6K1 was increased (Fig. 5D,F; corresponding full blot in supplementary Figure 13ii).

Microarray data of mTORC2 components (Table 2) revealed that RPTOR-independent Companion of mTORC2 (RICTOR) was up-regulated (twofold), while mitogen activated Protein Kinase associated Protein 1 (mSIN1) (1.15-fold) and Proline rich 5-like protein (PROTOR) (1.3-fold) were relatively unchanged. The downstream genes Protein Kinase B (AKT) (0.8-fold) and Protein Kinase C (PKC) (1.4-fold) showed relatively unchanged expression while Serum Glucocorticoid regulated Kinase (SGK) was up-regulated (2.9-fold) (Table 2).

Western blot revealed that while RICTOR was over-expressed, mSIN1 was relatively unchanged (Fig. 5G,H; corresponding full blot in supplementary Figure 14i (RICTOR) and ii (mSIN1)), consistent with the microarray data. On the other hand, AKT expression showed decreasing trend, consistent with the microarray data. mTORC2-dependent autophagy is regulated by the phosphorylation of the target proteins such as AKT (Ser473)^33^. Ser473 of AKT was hyperphosphorylated (Fig. 5G,I; corresponding full blot in supplementary Figure 15iii), while Thr308 of AKT, was also hyperphosphorylated, which indicates complete activation (Fig. 5G,I; corresponding full blot in supplementary Figure 15ii). To confirm this, we searched the transcriptomics data for down-stream targets of AKT and found p53 E3 Ubiquitin Protein Ligase (Mouse double minute 2 or MDM2) (3.1-fold), Stromal Interaction Molecule 1 (STIM 1) (2.1-fold), Conserved Helix-loop-Helix Ubiquitous Kinase (Chuk) (1.8-fold) were over-expressed (Table 2). Further, mSIN1, the mTORC2 inhibitor was significantly hypo-phosphorylated, thereby lowering its inhibitory effects and potentially facilitating AKT phosphorylation and mTORC2 mediated autophagy (Fig. 5G,I; corresponding full blot in supplementary Figure 15i). These data confirm mTORC2-dependent autophagy in the 3-NPA model.

### Cellular interaction between apoptosis and autophagy in the 3-NPA model

**Figure 6:**
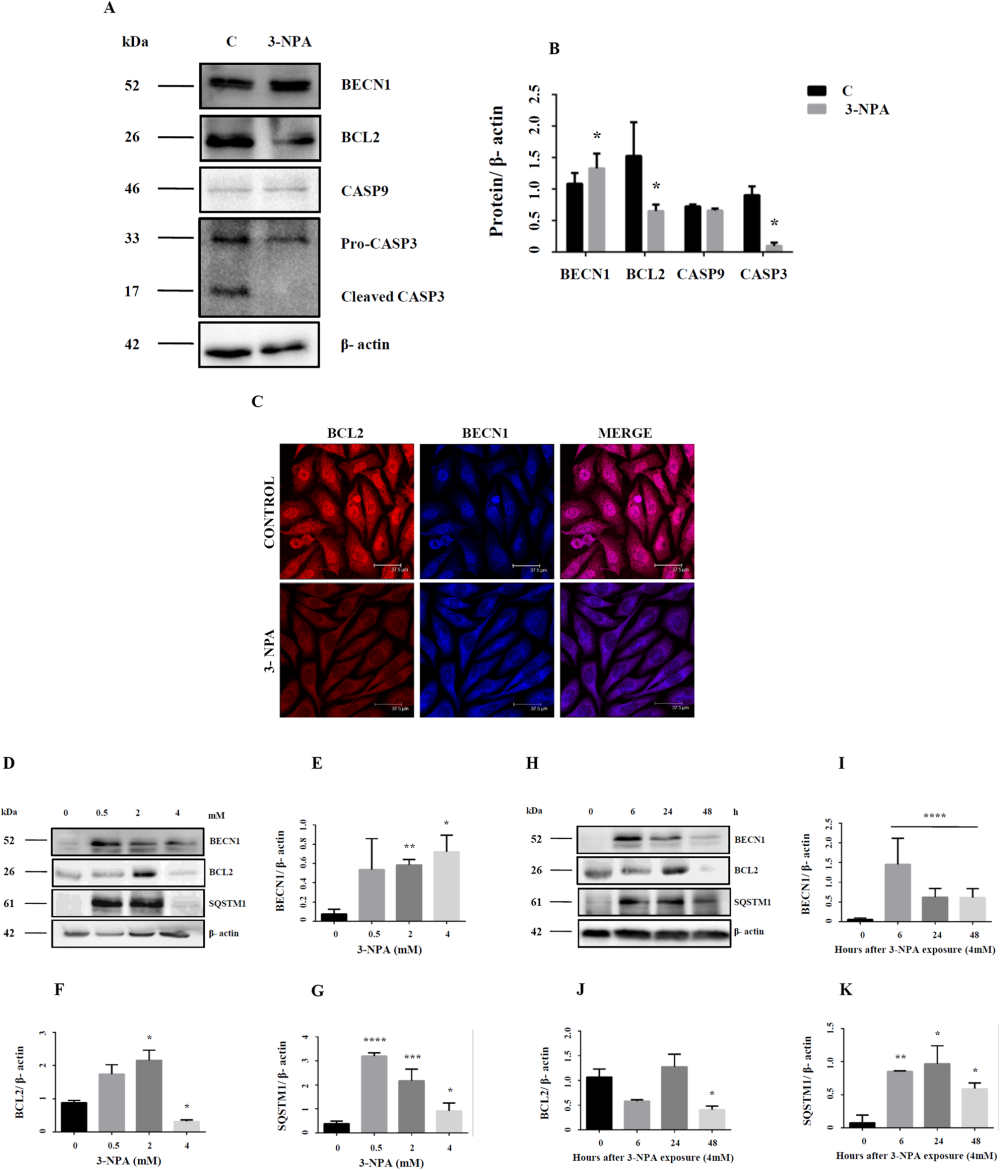
Dynamics between apoptosis and autophagy in 3-NPA treated N27 cells. BECN1, a key molecule in autophagy that balances autophagy and apoptosis was up-regulated (**A**,**B**, corresponding complete blot in supplementary Figure 16i) in 3-NPA treatment. Apoptotic markers BCL2 and CASP3 were down-regulated (**A**,**B**, corresponding complete blots in supplementary Figure 17i and vii) whereas CASP9 was slightly lowered but not statistically significant in 3-NPA treated cells (n = 3 trials per experiment; *p < 0.05). In case of CASP3, along with lowered pro-caspase levels in 3-NPA, the active form was also not generated. (**C**) Representative confocal images of control and 3-NPA treated cells stained with BCL2 and BECN1 vs. control (n = 3 coverslips per group). Dose (0–4 mM) (**D**–**G**, corresponding complete blots in supplementary Figure 16ii (BECN1), 17ii (BCL2) and iv (p62)) and time (0–48 h) (**H**–**K**, corresponding complete blots in supplementary Figures 16iv (BECN1), 17iii (BCL2) and vi (p62)) dependence of autophagic and apoptotic markers are shown in 3-NPA vs. control samples (n = 2 trials per experiment; *p < 0.05, **p < 0.01, ***p < 0.001, ****p < 0.0001). The western data for all the proteins in this figure are from independent blots. All blots were cut to retain only the region depicting the protein of interest.

A cross-talk between autophagy and apoptosis exists, with both regulating each other^34,35^. Certain genes play a dual role in both phenomena^34^. Beclin1-BCL2 complex for instance defines the balance between apoptosis and autophagy^34,35^. While Beclin1 was up-regulated, BCl2 expression was down-regulated in the 3-NPA model (Table 2) (Fig. 6A–C; corresponding full blot in supplementary Figure 16i (BECN1) and 17i (BCL2)). While BCL2 was localized primarily in the nucleus, 3-NPA treatment induced BCL2 labeling both in the nucleus and cytoplasm (Fig. 6C) with potential role in regulating apoptotis^36,37^. Microarray data also confirmed down-regulation of BCL2-dependent genes BCL2l11, BCL2l12, BCL3 and BNIP2. Further, we observed that 3-NPA treatment not only lowered the caspase 3 expression (quantification shown in Fig. 6B is for pro-caspase-3 band), but the cleaved active form was also minimal (Fig. 6A; corresponding full blot in supplementary Figure 17vii). Caspase-9 was showed slightly lowered expression, which was statistically not significant (Table 2) (Fig. 6A,B; corresponding full blot in supplementary Figure 16v). Since both autophagy and apoptosis is dynamic process, dose and tome dependent experiments were carried to validate the relative involvement of these pathways. We observed that in the lower doses, BCL2 expression was several fold higher compared to control, which then drastically decreased at the LD_50_ concentration (Fig. 6D,F; corresponding full blot in supplementary Figure 17ii). However, at earlier time points of 3-NPA treatment at 4 mM, BCL2 expression remained unchanged, which subsequently decreased 48 h (Fig. 6H,J; corresponding full blot in supplementary Figure 17iii). On the other hand, BECN1 was significantly upregulated at all time points and doses to different extents (Fig. 6D,E,H,I; corresponding full blot in supplementary Figure 16ii (dose) and iv (time)). SQSTM1 (p62) expression which was significantly elevated at the earlier stages and doses, decreased at LD_50_ but was found to be relatively higher compared to control (Fig. 6D,G,H,K; corresponding full blot in supplementary Figure 17iv (dose) and vi (time)). Considering these data, we speculate that at earlier stages of 3-NPA toxicity, both apoptotic and autophagic pathways are activated. However, at the later stages, owing to the downregulation of apoptotic proteins such as BCL2, along with consistently higher autophagic markers (BECN1 and p62) and reduced conversion of pro-caspase 3 to its active form (Fig. 6A,B), the cells may prefer autophagy over apoptotic pathways.

### Potential role of neurotrophic factors in enhancing autophagy in the 3-NPA model

**Figure 7:**
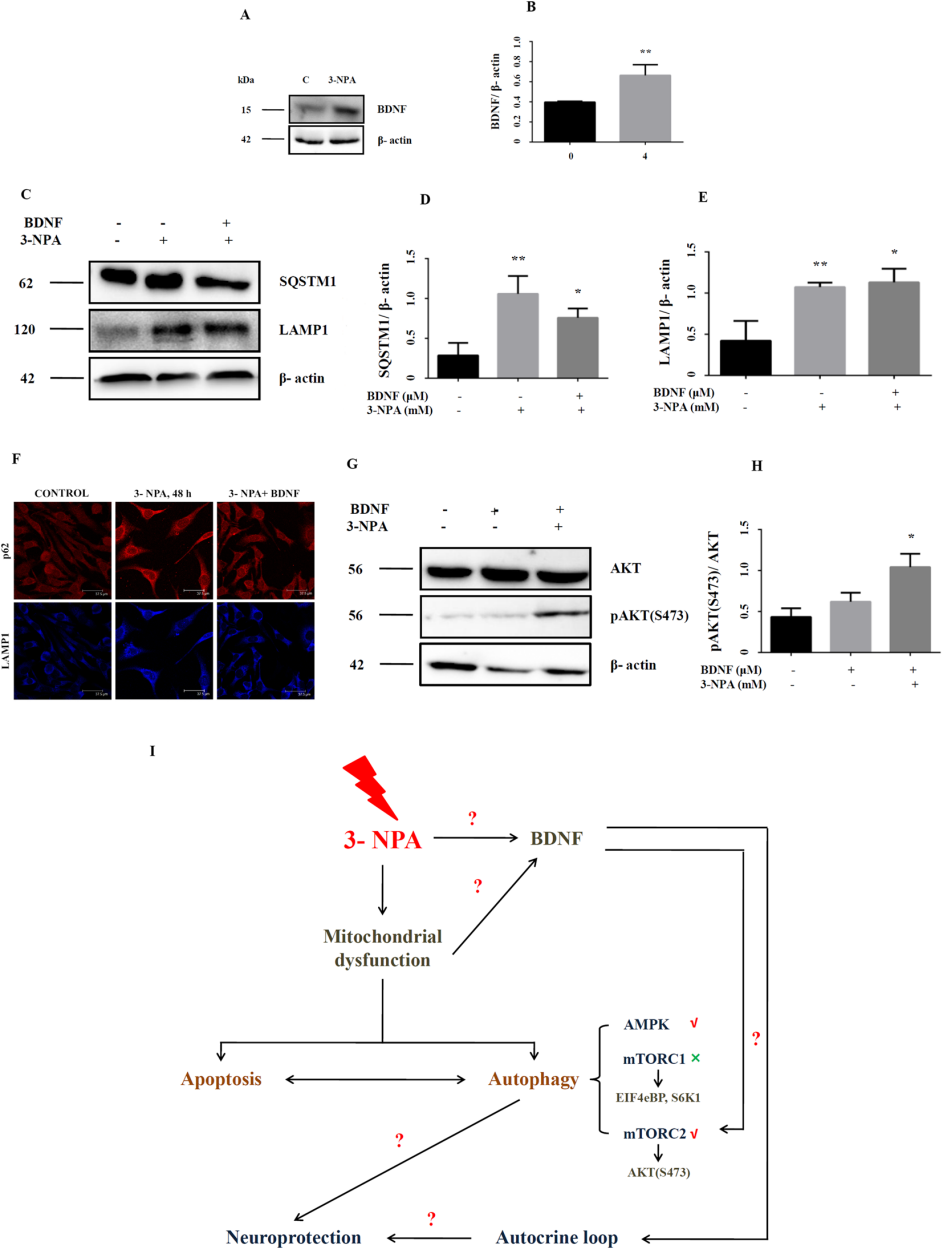
Growth factor mediated neuroprotection in 3-NPA treated N27 cells. Consistent with the microarray data, BDNF showed increased expression at the protein level (**A**,**B**, corresponding complete blot in supplementary Figure 18i). Co-treatment of 3-NPA treated N27 cells with BDNF could activate mTORC2 pathway as indicated by levels of pAKT (S473) (**G**,**H**, corresponding complete blot in supplementary Figure 18v). Autophagy was tending towards completion as indicated p62/SQSTM1 (**C**,**D**, corresponding complete blot in supplementary Figure 18ii) and LAMP1 (**C**,**E**, corresponding complete blot in supplementary Figure 18iii) in BDNF + 3-NPA co-treatment (n = 3 trials per experiment; *p < 0.05, **p < 0.01). This was validated further by immunocytochemistry (**F**) (n = 3 coverslips per group). (**I**) Depicts the schematic diagram summarizing the key findings of the study. 3-NPA mediated mitochondrial dysfunction alters the interaction and the ensuing balance between autophagy and apoptosis in neuronal cells with autophagy potentially conferring neuroprotection [Schematic was generated using Microsoft Powerpoint (2004)]. Autophagy pathway in the 3-NPA model entails mTORC2 (and not mTORC1) pathway and AMPK. 3-NPA mediated up-regulation of BDNF could positively influence neuroprotection either directly via autocrine loop or indirectly via upregulating the autophagy mechanism. The western data for all the proteins in this figure are from independent blots. All blots were cut to retain only the region depicting the protein of interest.

Trophic factors such as BDNF protect against cell death by enhancing autophagy^33,38^, although contradictory evidence exists^27^. Microarray data in the 3-NPA model revealed over-expression of BDNF (1.8-fold) and the same was confirmed by RT-PCR (Fig. 1D) and western blot (Fig. 7A,B; corresponding full blot in supplementary Figure 18i). We tested whether exogenous BDNF could promote autophagy in the 3-NPA model. Supplementation of the 3-NPA model with BDNF lowered p62 levels (Fig. 7C,D; corresponding full blot in supplementary Figure 18ii), while LAMP1 expression was unchanged (Fig. 7C,E; corresponding full blot in supplementary Figure 18iii), which was confirmed by immunocytochemistry (Fig. 7F). AKT phosphorylation could hasten the completion of autophagy by increased fusion of p62 with lysosome via re-organization of the cytoskeleton^39^. We observed increased phosphorylation of AKT at S473 (Fig. 7G,H; corresponding full blot in supplementary Figure 18v), which indicates AKT activation, with potential down-stream effects^39^.

## Discussion

### 3-NPA neurotoxicity entails unique cellular and transcriptomic profile

We investigated whether CII inhibitor triggers pathways that are different from CI inhibitor vs. metal toxin in neurons. This has implications for movement disorders and neurodegeneration as indicated in our previous study on Mn and MPP^+^ models^21^. Since the concentration of the three toxins are varied, it is challenging to emphatically state that the differences between CII and CI events are not influenced by dose-dependent effects of the toxin. Initial experiments with 3-NPA at concentrations comparable to Mn and MPP^+^ did not show any significant changes. Further, changes in established parameters such as ROS and GSH/GSSG could be noticed with highest significance at LD_50_ of 3-NPA (4 mM, 48 h). These data indicate that 3-NPA is a relatively weaker toxin, effective only at higher doses in this cell line compared to Mn and MPP^+^. The current study revealed autophagy pathway to be uniquely activated in the 3-NPA model. Mitochondrial dysfunction and autophagy in the 3-NPA model have been noted previously, but the downstream mechanisms were not explored in detail^18,20,40,41^.

3-NPA neurotoxicity is associated with apoptosis^2,42,43^. Our data revealed CASP9 levels to be unchanged in the 3-NPA model, while CASP3 was lowered (Fig. 6), consistent with other data^44^. 3-NPA probably activates calpain and cytochrome C relocalization that could downregulate CASP3 and CASP9^44^ and support cell survival. This could be possible in the 3-NPA cell model, since our transcriptomics data revealed up-regulation of Calpain 8 (CAPN8; 11.2-fold) and Calpain small subunit (CAPNS2; 2.6-fold).

In silico modelling using canonical pathway definitions revealed that autophagy could be triggered exclusively in the 3-NPA model with potential neuroprotective effects. Although one of the issues with pre-defined pathways is a lack of specificity for different experimental conditions and in turn inability to account for different cellular processes; the calculated N27 co-sets provided a means of calculating specific, functional pathways and subsequent characterization of biochemical alterations in the three models. The 3-NPA model was consistent with autophagy, with specific co-sets and associated metabolic reactions preferentially activated only in 3-NPA but not others (see Supplementary information spreadsheets, ‘mn_mpp_npa_vs_ref_difference’ tab). These can be seen as providence of metabolic substrates released and metabolized to promote autophagy, such as ubiquitination of proteins and long chain fatty acid oxidation, requiring peroxisomal, mitochondrial inositol metabolism (Fig. 3F,G).

The FBA analysis of the GeMMs comparing the fluxes in the 3 models in conjunction with the different co-sets led to interesting observations. Independent of any direct regulatory effects, the flux through CII is predicted to be lowered in the 3-NPA model, but increased in Mn and MPP^+^ models (Fig. 3D). Further, heme co-set (Fig. 3G and Supplementary information spreadsheets) was identified as being a principal pathway accounting for the altered flux states. Thus, the decreased CII flux may be in large part accounted for by increased heme metabolism in autophagy through succinyl-CoA metabolism. Additionally, glutathione synthesis is increased in 3-NPA but decreased in the other 2 models, potentially implying autophagy-specific mechanism.

### 3-NPA induces incomplete autophagy in N27 cells

We noted ultrastructural evidences of incomplete autophagy^45^ only in the 3-NPA and not in Mn or MPP^+^ models (Fig. 4). Autophagy genes revealed differential regulation (Table 2) both at the mRNA and protein level in the 3-NPA model. For example, GABARAPL1 which typically associates with autophagic vesicles^46^ was up-regulated by 2.5-fold. LC3 I to II conversion and LAMP1 was higher in 3-NPA treated cells (Fig. 4). However, p62 mRNA and protein levels were consistently higher (Figs. 1G, 4). p62, a multifunctional protein that interacts with LC3 is down-regulated with progression of autophagy^47^ probably indicating incomplete autophagy^48^. Astrocytes exposed to 3-NPA demonstrated incomplete autophagy with increased accumulation of p62^49^.

Proteins BCL2 and BECN1 maintain a delicate balance between autophagy and apoptosis^34,35^. Dissociation of BECN1 from BCL2 promotes autophagy. We observed up-regulation of BECN1 with a corresponding down-regulation of BCL2 in 3-NPA treated cells (Fig. 6)^50^. BECN1 is regulated by ubiquitination of TRAF6, which disrupts its interaction with BCL2, thereby inducing autophagy^51^. Our microarray data revealed twofold up-regulation of TRAF6 (Table 2), which could indirectly induce autophagy.

Death associated protein kinases (DAPKs) govern cell death by interacting with apoptotic or autophagic proteins. DAPK1, which was up-regulated in 3-NPA treated cells (2.7-fold), participates in autophagy and interacts with MAP1B which was up-regulated (3.2-fold). LC3 belongs to the family of MAP1 proteins and interacts directly with phosphorylated MAP1B^52^. Apart from activated AMPK^53^, DAPK can also phosphorylate BECN1 and weaken its interaction with BCL2, thereby facilitating autophagy^54,55^.

### mTORC2 is the chosen pathway in 3-NPA mediated autophagy: role of RICTOR and not RAPTOR

mTOR and AMPK are metabolic checkpoints in autophagy^56,57^. mTOR exists as mTORC1 and mTORC2; although mTORC2 is relatively less explored. mTORC1 has 4 subunits: DEPTOR, mLST8, RAPTOR and PRAS40. Pathways coping with metabolic stress impinge on mTORC1, whose inhibition promotes autophagy^32^. mTORC1 activates the downstream targets eIF4eBP and S6K1 via phosphorylation. DEPTOR, RAPTOR and PRAS40, when activated, negatively regulate mTORC1, thereby promoting autophagy. We noted evidences of mTORC1 inhibition in the 3-NPA model. While mTOR was up-regulated, RAPTOR was unchanged (Fig. 5). PRAS40 was hypo-phosphorylated, which could negatively regulate mTORC1. mTORC1 interacting protein PIH1D1, that positively regulates its assembly and S6K phosphorylation^58^ was down-regulated (0.4-fold). However, phosphorylation of S6K1 was slightly higher, despite unchanged expression, which could be due to phosphorylation via AKT^59^.

Inhibition of mTORC1 could happen via AMPK. During cellular energy crisis, AMPK [activated in 3-NPA model (Fig. 5)], can regulate mTORC1 by phosphorylating TSC2 and RAPTOR^32,57^. Proteins including KSR1-2 and Sestrins (SESN1-3) can activate AMPK. For instance, SESN2 (up-regulated by ~ 3.1-fold) which is stress inducible and regulated by p53 inhibits mTORC1 by activating AMPK via forming AMPK-TSC1:TSC2 complex^60–62^. Transcriptomics data showed up-regulation of other p53 associated proteins, PERP (~ twofold), Tp53I11 (1.8-fold) and Tp53RK (1.82-fold). Similarly, KSR1 and KSR2 can physically interact with and activate AMPK^63,64^. KSR in turn is activated by CNK1 (up-regulated by ~ 2.2-fold), which acts upstream to RAF in RAS pathway connecting it with KSR^62^.

On the other hand, our study revealed activation of mTORC2 pathway leading to autophagy. mTORC2 has five subunits (DEPTOR, RICTOR, mLST8, mSIN1 and PROTOR) and its activation phosphorylates the downstream target AKT at ser473, via up-regulation of RICTOR^63^ (Fig. 5). This process is regulated by mSIN1^65^, whose phosphorylation at T86 dissociates if from RICTOR, impairing its catalytic activity and abolishing AKT (S473) phosphorylation^66^. DEPTOR also negatively regulates mTORC2, and its overexpression could promote cell survival by inhibition of mTORC1 pathway and activation of PI3K/AKT pathway^67^. Previous studies^29,33^ have clearly suggested that AKT regulates mTOR mediated autophagy and phosphorylation at Ser473 of AKT is a read out for mTORC2 activation. Further, our data also showed hyperphosphorylation of AKT at T308 (Fig. 5G–I), which indicates complete activation of AKT leading to autophagy^33,39^. Hence, 3-NPA treatment could induce autophagy via mTORC2 and AKT.

### BDNF offers neuroprotection against 3-NPA neurotoxicity

Both mTORC1 and mTORC2 are regulated by growth factors^32,33,65^. Growth factor regulates mTORC1 at the level of TSC complex via PI3K and AKT^68^ and activates TORC2 partly by PI3K^29^. We noted significant up-regulation of BDNF mRNA and protein (Figs. 1D, 7A,B). BDNF can activate mTORC1, which in turn induces p70S6K1 phosphorylation and expression^69^, but this can be over-ridden by AMPK at the level of TSC2 and Raptor^70^. BDNF can also regulate phosphorylation of AKT at S473^68,70,71^ probably via PI3K.

Growth factors can also activate Rictor and promote pAKT (S473)^33^. Co-treatment with 3-NPA and BDNF elevated pAKT (S473) in N27 cells (Fig. 7G,H). It is possible that growth factor increases phosphatidylinositol triphosphate [PtdIns (3, 4, 5) P3] content, which associates with the SIN1-PH domain and in turn recruit mTORC2 to the plasma membrane, enabling pAKT (S473)^65^. This could in-turn regulate mTORC1 activity and autophagy as evidenced by LAMP1 and p62 levels (Fig. 7C–E). mTORC2 can be regulated via syndecan proteins, which act as co-receptors for growth factors^72–74^ and these were up-regulated exclusively in the 3-NPA model (SDC2-1.9-fold; SDC3-4.4-fold). Upon growth factor stimulation, they target PKCa to the plasma membrane eventually recruiting mTORC2 and activate AKT^75^.

BDNF can confer neuroprotection by suppressing autophagy via activation of mTORC1 and regulating the activity of ULK2^27,76,77^. Apart from mTORC1, mTORC2 is also required to regulate autophagy^39,78^. Our study also speculates a similar scenario, wherein upon growth factor stimulation, there is increased activity of mTORC2 as depicted by higher pAKT (S473) and greater turnover of autophagosomes as depicted by p62 and LAMP1 (Fig. 7). BDNF treatment and subsequent increase in mTORC2 activity in 3-NPA treated cells could promote clearance of autophagosomes by increasing the rate of endocytosis. However, we would like to state that our data does not conclusively prove that BDNF induces autophagy rather promotes the ongoing autophagic process such that it leads to completion.

To summarize (Fig. 7I), the current study proposes that (i) different neurotoxins could potentially activate distinct downstream mechanisms, implicating that degenerative changes among neurodegenerative diseases may not be common (ii) the inter-relationship between apoptosis and autophagy could be one of the important regulatory mechanisms in 3-NPA neurotoxicity (iii) Autophagy in the 3-NPA model entails mTORC2 (iv) growth factors such as BDNF could promote ongoing autophagic process.

## Materials and methods

The chemicals, reagents and solvents used were of analytical grade and obtained from Sisco Research Laboratories (Mumbai, Maharashtra, India) and Merck (Whitehouse Station, NJ, USA). Fine chemicals, cell culture media, KiCQ start PCR primers and anti-β-actin antibody were obtained from Sigma-Aldrich (St. Louis, MO, USA). Fetal Bovine Serum was procured from PAN Biotech (GmbH, Germany). DNA extraction kit and cDNA conversion kit were procured from Qiagen (Venlo, Netherlands) and Applied Biosystems (Foster city, CA, USA) respectively. SyBR green was procured from Takara Bio (Kusatsu, Japan). Electrophoresis and Western blot reagents were from Bangalore Genie (Bangalore, Karnataka, India), Bio-Rad Laboratories (Hercules, CA, USA) and Millipore (Billerica, MA, USA). ATP: ADP assay kit was obtained from Abcam (Cambridge, MA, USA). All primary antibodies used for immunoblotting and immunocytochemistry (Supporting Table 1) were obtained from Abcam and Cell Signaling Technology (Danvers, MA, USA). HRP-conjugated secondary antibodies were obtained from Bangalore Genei. Reagent kits and other consumables for transcriptomics (cDNA microarray) were obtained from Agilent Technologies (Santa Clara, CA, USA). HPLC columns were obtained from Waters Corporation (Milford, MA, USA) and other consumables from Sigma-Aldrich and Sisco Research Laboratories.

### Cell culture experiments

#### Cell culture, cell viability and preparation of cell extract

Rat 1RB3AN27 (N27) dopaminergic neuronal cell line was used throughout the study. Cells were cultured and maintained as described^79,80^. Cell viability following treatment with toxins was assessed by 3-(4,5-dimethylthiazol-2-yl)-2,5-diphenyltetrazolium bromide (MTT) dye reduction method^18^.

For preparation of soluble extracts, cells after different treatments were harvested in 1× Phosphate buffered Saline (1× PBS) containing protease inhibitor cocktail and lysed in a sonicator (Sonics Vibra Cell, Sonics and Materials Inc., Newtown, CT, USA) on ice (10 s × 5 cycles) and centrifuged (13,000×*g* for 10 min at 4 °C). The supernatant was subjected to biochemical assays and SDS-PAGE following protein estimation^81^.

### Redox assays

Total Hydroperoxides in soluble cell extracts were estimated using Amplex Red assay kit (Thermo Fisher Scientific, Carlsbad, CA, USA), as per the the manufacturer’s instructions. GSH/GSSG ratio was determined by o-pthalaldehyde method^82^. Glutathione-S-Transferase (GST) assay was carried out as described^83^ and the activity was measured as the rate of enzyme catalyzed conjugation of GSH with 1-chloro-2,4-dinitrobenzene (CDNB).

### Mitochondrial assays

Mitochondrial complex II assay was assessed as described^84,85^. Mitochondrial membrane potential was estimated using JC1 fluorescent dye as described^21^ in control and 3-NPA treated N27 cells.

ADP/ATP ratio was determined in cells using a commercially available assay kit (Abcam), according to the instructions of the manufacturer.

NAD^+^/NADH ratio was determined by HPLC as described^86–88^ with minor modifications. Cells were sonicated, centrifuged (10,000*g* for 10 min at 4 °C) and the supernatant was treated with equal parts (vol/vol) of the mobile phase [50 mM Ammonium Acetate, pH 5.3 and Acetonitrile at 70: 30 (vol/vol)]. This mixture was centrifuged (10,000*g* for 10 min) to separate the precipitated proteins and the supernatant was directly injected with a Hamilton syringe into Waters 1525 binary HPLC system fitted with C18 column (5 µm; 4.6 × 250 mm; SunFire analytical columns; Waters) and run with a flow rate of 1 ml/min with a total run time of 10 min. Both NAD^+^ and NADH were detected by UV-detector (Waters 2489-UV/Visible detector) at 260 nm and 340 nm respectively at a retention time of 2 min.

### Ultrastructural analysis

Cell pellets from the control and treatment groups were fixed in 3% buffered glutaraldehyde and sectioned using Leica UC6 ultramicrotome. Staining of sections was carried out using uranyl acetate and lead citrate following which they were scanned under FEI Tecnai G^2^ Spirit Biotwin Transmission Electron Microscope^89^.

### Whole genome microarray

Whole genome cDNA microarray was performed on control and 3-NPA treated cells as described^21^. N27 cell pellets were harvested and preserved in RNA-later (Thermo Fischer Scientific). Total RNA from cells was extracted using Qiagen’s RNeasy minikit according to the manufacturer’s instructions and its purity was assessed by NanoDrop ND-1000 UV–Vis Spectrophotometer. RNA quality was determined by rRNA 28S/18S ratio and RNA integrity number (RIN)^90^.

Total RNA from different groups were labelled using Quick-Amp Labeling Kit, One Color (Agilent Technologies), reverse transcribed at 40 °C using oligo dT primer tagged to a T7 polymerase promoter and converted to double stranded cDNA. The double stranded cDNA thus synthesized was used subsequently as template for cRNA generation. Dye Cy3 CTP (Agilent) was incorporated following cRNA generated by in vitro transcription. The cDNA synthesis and in vitro transcription steps were carried out at 40 °C. Labelled cRNA was cleaned up using Qiagen RNeasy columns (Qiagen, Cat No: 74106) and assessed for yields and specific activity in Nanodrop ND-1000. The labelled cRNA sample fragmented at 60 °C was hybridized on to Agilent Rat whole genome Gene Expression Microarray (AMADID: 28279). Fragmentation of labelled cRNA and hybridization were done using the Gene Expression Hybridization kit of Agilent Technologies (In situ Hybridization kit, Part Number 5190-0404) in Agilent’s Surehyb Chambers at 65 °C for 16 h. The hybridized slides were washed using Agilent Gene Expression wash buffers (Agilent Technologies, Part Number 5188–5327) and scanned using the Agilent Microarray Scanner (Agilent Technologies, Part Number G2600D). Raw data extraction from Images was obtained and quantified using Agilent Feature Extraction software (v11.5, Agilent technologies)^90^. Signal data from the images were extracted, analysed, background corrected and normalized by Loess normalization method.

Normalization of the data was done in GeneSpring GX using the 75th percentile shift and fold expression values for test samples were obtained with respect to specific control samples. Significant genes up-regulated [> 0.8-fold (log base2) signal] and down regulated [< 0.8-fold (log base2) signal] in the 3-NPA test group were identified. Statistical methods including student t- test p-value and False Discovery Rate (FDR) were employed to identify significant differentially regulated genes. Differentially regulated genes were clustered using hierarchical clustering algorithm to identify significant gene expression patterns. Genes were classified based on functional category and pathways using GeneSpring GX and Genotypic Biointerpreter-Biological Analysis Software^91^. Shortlisted genes were further validated by qRT-PCR and/or western blotting from control and treated N27 cells.

### Quantitative real-time PCR

mRNA was extracted from cells of control and treated groups and converted to cDNA using a commercially available kit (Thermo Fisher Scientific) according to the manufacturer’s instructions. Pre-designed qPCR primers (KiCQ start primers, Sigma-Aldrich) were used along with SyBR green chemistry (Thermo Fisher Scientific) to analyze gene expression. Gene expression was normalized to GAPDH and expressed as fold change (2^−ΔΔCt^) with respect to control^21,92^.

### SDS-PAGE and western blotting

Total proteins in the soluble cell extract from different groups in equal quantity was resolved by SDS-PAGE using the electrophoresis apparatus from Biorad Laboratories Inc, followed by western blotting with antibodies of interest as described^93^. For most of the blots care was taken to retain the full length blot for hybridization of antibody and acquisiation of images. In experiments where the primary antibody availability was limited, the nitrocellulose membrane containing the transferred protein was cut based on the molecular weight so as to retain only the areas corresponding to the protiens of interest prior to the treatment with the desired primary antibody or to perform anti β-actin western. This information has been highlighted in the appropriate figure legend in the supplementary information file. β-actin served as loading control. Western signal on the blots was recorded in a gel documentation system (Biorad Laboratories Inc.) and the images were analyzed using ImageJ software^94^ and normalized to their respective loading controls. The corresponding complete blots have been provided in the supplementary information.

### Immunocytochemistry

Control/3-NPA treated N27 cells grown on poly-l-Lysine coated coverslips were fixed with 100% methanol for 10 min at 4 °C. The coverslips were washed thrice with 1× PBS and the cells were permeabilized for 15 min in room temperature with staining buffer [1× PBS with 0.1% Tween-20 (PBST)]. Non-specific staining was blocked with 1.5% bovine serum albumin (BSA) dissolved in PBST for 2 h and the cells were treated with primary antibody (prepared in 0.15% BSA in PBST) overnight at 4 °C with continuous shaking. Primary antibody was washed with 1× PBS and the cells were incubated with suitable secondary antibody (Cy3/Cy5/FITC conjugated), at room temperature for 2 h. The secondary antibody was washed thoroughly and all the steps commencing from blocking were repeated with a different set of primary/secondary antibody in case of co-labeling experiments. The washed coverslips were mounted and visualized using confocal laser scanning microscope (Leica TCS-SL; Leica, Wetzlar, Germany/Flow view 10i, Olympus Corporation, Japan).

### Generation of in silico models

#### Network interpretation of differentially expressed transcripts

Differentially expressed transcripts (based on pairwise comparisons between toxin treated and untreated controls) were used to define reaction sub-networks for each treatment condition. The gene-reaction-protein (GRP) associations were used to map the up- and down-regulated gene loci to the corresponding reactions. The metabolites involved in each reaction were then selected and grouped by sub-cellular organelles, providing an organelle-enrichment assessment for each toxin treatment condition.

#### Context-specific genome scale metabolic model (GeMM) analysis

Transcriptomic data from four different conditions (untreated, Mn, MPP^+^, and 3-NPA treated) was used to generate context specific genome scale metabolic networks for functional analysis of the cellular metabolic capabilities of the treated and untreated N27 cells. As with the organelle analysis, the NCBI Homologene database (Release 68, https://www.ncbi.nlm.nih.gov/homologene) was used to generate a global RatCon model using Recon1^95^.

The gene inactivity moderated by metabolism and expression (GIMME)^96^ approach was used to construct context specific models of metabolism using the biomass pseudo-reaction at 50% of the maximal value of RatCon model. The benefit of this approach is that presence/absence calls are used for the incorporation of transcriptomic data, since in general, gene expression is not linearly, quantitatively correlated with flux levels for the corresponding transcribed protein. However, transcripts and the corresponding enzymes that they encode are highly associated, qualitatively, i.e. through presence/absence calls; a cut-off of 11 for the RNA-seq data was used (based upon largest incremental change in number of transcripts). Three different iterations of GIMME were performed (by randomly re-sorting the order of reactions) for each experimental condition. Since condition specific uptake measurements were not available, ‘rich media’ conditions were used to specify the uptake bounds (Supporting information spreadsheets). The same uptake conditions were used for each of the experimental conditions (Untreated, Mn treated, MPP^+^ treated, and 3-NPA treated). The solution spaces of each model were uniformly sampled for 5000 points using the artificially centered hit and run (ACHR) algorithm described by Kaufman and Smith (Operations Research, v46, 1998)^97^. A BDNF demand reaction was constructed by obtaining the FASTA sequence (https://www.ncbi.nlm.nih.gov/protein/XP_011518582.1?report=fasta) with the appropriate stoichiometry for the composite amino acids. In order to force the production of BDNF, a lower bound of 0.000001 was specified for all of the models prior to running GIMME; this bound was reset to 0 during all subsequent simulations (e.g. sampling the steady state solution space, etc.).

Since context-specific uptake conditions were not available, there was not a need to normalize the sampled points prior to merging the points from the different GIMME iterations. Only the reactions that were present in all three models (for each of the four conditions) were used for subsequent analysis. A numerical cut-off of 1 × 10^–7^ was used for the sampled points.

The size of the null space of the 9 models ranged from 537 to 557. Each of the 12 models (three different iterations for each set of data, as above) was sampled for 5,000 points. Only reactions that were included in all three GIMME models for each data set were retained, resulting in 4 different models (untreated, Mn, MPP^+^, and 3-NPA), each with 15,000 sampled points. Correlated reaction sets were calculated for correlation coefficients greater than or equal to 0.975 (or R-squared > 0.95).

Co-set analysis focused on the 23 largest co-sets (i.e. co-sets that involved at least 6 reactions), as the smaller reaction co-sets involved 5 or fewer reactions, in which generally two or more reactions were transport reactions. Since the focus of the differences was on intracellular metabolism and enzymatic conversions, these smaller co-sets were not of significant interest.

Hierarchical clustering of the reaction co-sets was performed by selecting the same reaction flux from each co-set under the different conditions and clustering the maximum value of the flux as a percentage of the sum of the flux across all conditions (in order to provide an assessment of the relative increase or decrease of the corresponding co-set between the different conditions).

Differentially expressed reactions between the untreated and three different drug treatment conditions, as well as pairwise comparisons between the different drug treatment conditions were identified based on p < 0.05 following the Bonferroni correction, in addition to at least twofold difference in mean flux between the two conditions and absolute mean flux < 1000 arbitrary flux units. Flux balance analysis (FBA) simulations were carried out using Gurobi optimization software (v7.0.2, Beaverton, Oregon), Matlab (v2015b, The MathWorks, Inc., Natick, Massachusetts), and the Cobra Toolbox^98^. Venn diagram of the differentially expressed genes in each neurotoxic model vs. control was generated using Python (Matplotlib library; URL—https://matplotlib.org/). Clustering of reaction co-sets for GeMMs for control, Mn, MPP^+^ and 3-NPA treated cells were generated using MATLAB (version 2015b). Pathway maps of the co-sets and reaction pathways were generated using Escher; URL—https://escher.github.io).

### Statistical analysis

All the experiments were carried out with replicates and have been highlighted against each experiment. With respect to the transcriptomics experiment blinding was followed and the experimenter was blinded during sample preparation from cell pellets obtained following 3-NPA treatment.

Cumulative data from three independent experiments (excluding transcriptomics, which was in duplicates) have been expressed as mean ± SD for the results from all in vitro experiments. Multiple comparisons were analyzed using ANOVA or student’s t-test in GraphPad Prism, Version 6.00 for Windows (GraphPad Software, La Jolla California USA, http://www.graphpad.com) and p < 0.05 was considered significant. For the microarray experiment, the data was checked for normal distribution. The data for both the up-regulated and the down-regulated data was analysed using non-parametric test, as the fold change was not normally distributed. Kruskal–Wallis H test was employed to test the fold change differences between different toxins. The fold change was kept as a dependent variable and the type of toxin as an independent variable.

## Supplementary Information

Supplementary Information 1.

Supplementary Information 2.

## Data Availability

The transcriptomics data included in the current submission is available at Gene Expression Omnibus (GEO) and can be accessed through the URL https://www.ncbi.nlm.nih.gov/geo/query/acc.cgi?acc=GSE92963.

## Abbreviations

CII: Mitochondrial complex II
3-NPA: 3-Nitropropionic acid
ROS: Reactive oxygen species
GSH: Glutathione (reduced)
GSSG: Glutathione (GSSG)
EM: Electron microscopy
H&E: Haematoxylin and eosin
CI: Mitochondrial complex I
MPP^+^: 1-Methyl-4-phenylpyridinium
Mn: Manganese
mTORC2: Mechanistic target of rapamycin complex
BDNF: Brain derived neurotrophic factor
GST: Glutathione-S-transferase
GeMM: Genome scale metabolic model
GIMME: Gene inactivity moderated by metabolism and expression
AMPK: AMP-regulated protein kinase
AKT: Protein kinase B
Bcl-2: B-cell lymphoma 2
