# Supplementary material for: Inhibition of mitochondrial complex II in neuronal cells triggers unique pathways culminating in autophagy with implications for neurodegeneration
Source: Sci Rep. 2021 Jan 15;11:1483. doi: 10.1038/s41598-020-79339-2 (PMC7810707; doi:10.1038/s41598-020-79339-2)

SUPPLEMENTARY INFORMATION

Inhibition of mitochondrial complex II in neuronal cells triggers unique pathways culminating in autophagy with implications for neurodegeneration

Sathyanarayanan Ranganayaki[*], Neema Jamshidi[+], Mohamad Aiyaz[&], Santhosh-Kumar Rashmi[%], Narayanappa Gayathri[%], Pulleri Kandi Harsha[$], Balasundaram Padmanabhan[^] and Muchukunte Mukunda Srinivas Bharath[#@]

From the [*]Department of Neurochemistry, National Institute of Mental Health and Neurosciences (NIMHANS), No. 2900, Hosur Road, Bangalore-560029, Karnataka, INDIA; [+]Department of Radiological Sciences, Ronald Reagan UCLA Medical Center, Los Angeles, California 90095, USA; [&]Genotypic Technology Pvt. Ltd., 2/13, Balaji Complex, 80 feet Road, RMV 2nd Stage, Bangalore-560094, Karnataka, INDIA; [%]Department of Neuropathology, NIMHANS, No. 2900, Hosur Road, Bangalore-560029, Karnataka, INDIA; [$]Department of Neurovirology, NIMHANS, No. 2900, Hosur Road, Bangalore-560029, Karnataka, INDIA; [^]Department of Biophysics, NIMHANS, No. 2900, Hosur Road, Bangalore-560029, Karnataka, INDIA; [#]Department of Clinical Psychopharmacology and Neurotoxicology, NIMHANS, No. 2900, Hosur Road, Bangalore-560029, Karnataka, INDIA.

Running title: *Complex II inhibition elicits unique cascade in neurons*

Supplementary table 1. List of antibodies used for immunoblotting and immunocytochemistry in the current study.

Sl. No.	Protein detected/ Primary antibody	RRID	Dilution	Secondary antibody
1.	LC3B (CST)	AB_915950	1:1000	Goat Anti- mouse
2.	SQSTM1 (Abcam)	AB_945626	1:1000	Goat Anti- mouse
3.	LAMP1 (Abcam)	AB_775978	1:1000	Goat Anti- rabbit
4.	mTOR (Abcam)	AB_303257	1:500	Goat Anti- rabbit
5.	RPTOR (Abcam)	AB_304901	1:500	Goat Anti- rabbit
6.	S6K1 (CST)	AB_331676	1:1000	Goat Anti- rabbit
7.	pS6K1(T389) (CST)	AB_330944	1:500	Goat Anti- rabbit
8.	EIF4eBP (CST)	AB_331692	1:1000	Goat Anti- rabbit
9.	pEIF4eBP (T46) (CST)	AB_560835	1:500	Goat Anti- rabbit
10.	PRAS40 (CST) (CST)	AB_916206	1:1000	Goat Anti- rabbit
11.	pPRAS40 (T246) (CST)	AB_916141	1:500	Goat Anti- rabbit
12.	AMPK (CST)	AB_330331	1:1000	Goat Anti- rabbit
13.	pAMPK (T172) (CST)	AB_331250	1:500	Goat Anti- rabbit
14.	RICTOR (CST)	AB_2179961	1:500	Goat Anti- rabbit
15.	mSIN1 (CST)	AB_2798048	1:1000	Goat Anti- rabbit
16.	pmSIN1 (T86) (CST)	AB_2798584	1:500	Goat Anti- rabbit
17.	AKT (CST)	AB_915783	1:1000	Goat Anti- rabbit
18.	pAKT (S473) (CST)	AB_329825	1:500	Goat Anti- rabbit
19.	pAKT (T308) (Abcam)	AB_722678	1:500	Goat Anti- rabbit
20.	BECN1 (Abcam)	AB_955699	1:1000	Goat Anti- rabbit
21.	BCL2 (Abcam)	AB_2064155	1:2000	Goat Anti- rabbit
22.	CASP3 (Abcam)	AB_443014	1:1000	Goat Anti- rabbit
23.	CASP9 (Abcam)	AB_448813	1:1000	Goat Anti- mouse
24.	BDNF (Abcam)	AB_10862052	1:1000	Goat Anti- rabbit
25.	Beta-actin (Sigma)	AB_476744	1:3000	Goat Anti- mouse

Supplementary table 2: List of up-regulated genes upon 3-NPA treatment. The Gene symbol, Gene name and fold change in expression (3-NPA/Control) are indicated.

Gene Symbol	Gene Name	Fold change
Abca4	ATP-binding cassette, subfamily A (ABC1), member 4	3.51
Abca8	ATP-binding cassette, subfamily A (ABC1), member 8	2.98
Abcb1b	ATP-binding cassette, subfamily B (MDR/TAP), member 1B	4.51
Abcc12	ATP-binding cassette, subfamily C (CFTR/MRP), member 12	1.82
Abcc2	ATP-binding cassette, subfamily C (CFTR/MRP), member 2	5.51
Abcc5	ATP-binding cassette, subfamily C (CFTR/MRP), member 5	1.89
Abcg3l1	ATP-binding cassette, subfamily G (WHITE), member 3-like 1	2.04
Abhd12b	abhydrolase domain containing 12B	3.13
Ablim3	actin binding LIM protein family, member 3	3.84
Abo	ABO blood group (transferase A, alpha 1-3-N-acetylgalactosaminyltransferase; transferase B, alpha 1-3-galactosyltransferase)	1.88
Acacb	acetyl-CoA carboxylase beta	2.48
Acad11	acyl-CoA dehydrogenase family, member 11	1.75
Acap1	ArfGAP with coiled-coil, ankyrin repeat and PH domains 1	4.15
Acap2	ArfGAP with coiled-coil, ankyrin repeat and PH domains 2	2.55
Ace	angiotensin I converting enzyme (peptidyl-dipeptidase A) 1	5.26
Acer2	alkaline ceramidase 2	3.60
Acot13	acyl-CoA thioesterase 13	1.80
Acot2	acyl-CoA thioesterase 2	1.86
Acot4	acyl-CoA thioesterase 4	1.88
Acoxl	acyl-CoA oxidase-like	2.06
Acp5	acid phosphatase 5, tartrate resistant	2.70
Acpt	acid phosphatase, testicular	1.97
Acr	acrosin	2.05
Acss1	acyl-CoA synthetase short-chain family member 1	2.22
Actg2	actin, gamma 2, smooth muscle, enteric	2.35
Actn3	actinin alpha 3	2.29
Actr3b	ARP3 actin-related protein 3 homolog B (yeast)	5.77
Actrt3	actin-related protein T3	2.15
Adam19	ADAM metallopeptidase domain 19	2.80
Adam8	ADAM metallopeptidase domain 8	3.10
Adamts15	ADAM metallopeptidase with thrombospondin type 1 motif, 15	1.82
Adamts2	ADAM metallopeptidase with thrombospondin type 1 motif, 2	3.28
Adamts7	ADAM metallopeptidase with thrombospondin type 1 motif, 7	2.46
Adap2	ArfGAP with dual PH domains 2	2.47
Adcy8	adenylate cyclase 8 (brain)	2.44
Add2	adducin 2 (beta)	5.53
Adh1c	alcohol dehydrogenase 1C (class I), gamma polypeptide	2.53
Adh7	alcohol dehydrogenase 7 (class IV), mu or sigma polypeptide	9.87

Adipoq	adiponectin, C1Q and collagen domain containing	3.14
Adora2a	adenosine A2a receptor	3.53
Adprhl1	ADP-ribosylhydrolase like 1	1.88
Aen	apoptosis enhancing nuclease	3.21
Afap1l1	actin filament associated protein 1-like 1	2.17
Afmid	arylformamidase	4.45
Afp	alpha-fetoprotein	11.96
Agap1	ArfGAP with GTPase domain, ankyrin repeat and PH domain 1	1.95
Agl	amylo-alpha-1, 6-glucosidase, 4-alpha-glucanotransferase	1.76
Ahnak2	AHNAK nucleoprotein 2	13.71
Ahr	aryl hydrocarbon receptor	2.06
Ahrr	aryl-hydrocarbon receptor repressor	2.32
Aifm3	apoptosis-inducing factor, mitochondrion-associated 3	2.21
Aipl1	aryl hydrocarbon receptor-interacting protein-like 1	1.89
Ak8	adenylate kinase 8	2.72
Akap12	A kinase (PRKA) anchor protein 12	3.29
Akap17b	A kinase (PRKA) anchor protein 17B	2.11
Akap7	A kinase (PRKA) anchor protein 7	1.81
Akr1b8	aldo-keto reductase family 1, member B8	2.60
Akr1c12	aldo-keto reductase family 1, member C12	5.01
Akr1c12l1	aldo-keto reductase family 1, member C12-like 1	1.80
Akr1c19	aldo-keto reductase family 1, member C19	3.36
Akr1c3	aldo-keto reductase family 1, member C3	2.18
Akr1e2	aldo-keto reductase family 1, member E2	2.29
Alcam	activated leukocyte cell adhesion molecule	6.46
Aldh18a1	aldehyde dehydrogenase 18 family, member A1	2.13
Aldh3a1	aldehyde dehydrogenase 3 family, member A1	2.69
Aldh3a2	aldehyde dehydrogenase 3 family, member A2	2.06
Aloxe3	arachidonate lipoxygenase 3	3.52
Alpk1	alpha-kinase 1	2.08
Ambp	alpha-1-microglobulin/bikunin precursor	2.76
Amigo2	adhesion molecule with Ig like domain 2	2.15
Ampd3	adenosine monophosphate deaminase 3	2.73
Amph	amphiphysin	2.59
Amt	aminomethyltransferase	2.23
Amz1	archaelysin family metallopeptidase 1	1.77
Ang	angiogenin, ribonuclease, RNase A family, 5	5.81
Ang1	angiogenin, ribonuclease A family, member 1	5.69
Angel2	angel homolog 2 (Drosophila)	1.95
Angpt1	angiopoietin 1	2.34
Ank2	ankyrin 2, neuronal	2.03
Ank3	ankyrin 3, node of Ranvier	1.76
Ankrd27	ankyrin repeat domain 27 (VPS9 domain)	2.49
Ankrd28	ankyrin repeat domain 28	2.58
Ankrd33b	ankyrin repeat domain 33B	1.86

Ano3	anoctamin 3	2.69
Anxa9	annexin A9	2.74
Aoc3	amine oxidase, copper containing 3 (vascular adhesion protein 1)	1.93
Aox1	aldehyde oxidase 1	2.90
Ap1ar	adaptor-related protein complex 1 associated regulatory protein	1.80
Ap3b2	adaptor-related protein complex 3, beta 2 subunit	7.36
Ap5s1	adaptor-related protein complex 5, sigma 1 subunit	2.85
Apba2	amyloid beta (A4) precursor protein-binding, family A, member 2	2.07
Apbb3	amyloid beta (A4) precursor protein-binding, family B, member 3	1.91
Apc	adenomatous polyposis coli	2.18
Apc2	adenomatosis polyposis coli 2	1.79
Apln	apelin	4.69
Apoa5	apolipoprotein A-V	1.84
Apobec1	apolipoprotein B mRNA editing enzyme, catalytic polypeptide 1	2.95
Apobec2	apolipoprotein B mRNA editing enzyme, catalytic polypeptide-like 2	2.07
Apoc2	apolipoprotein C-II	3.61
Apoe	apolipoprotein E	2.04
Apol9a	apolipoprotein L 9a	2.13
Apold1	apolipoprotein L domain containing 1	3.22
Aqp11	aquaporin 11	1.93
Arap3-ps1	ArfGAP with RhoGAP domain, ankyrin repeat and PH domain 3, pseudogene 1	2.68
Arc	activity-regulated cytoskeleton-associated protein	3.81
Areg	amphiregulin	5.81
Arg1	arginase, liver	3.52
Arg2	arginase type II	3.37
Arhgap20	Rho GTPase activating protein 20	4.08
Arhgap22	Rho GTPase activating protein 22	3.80
Arhgap32	Rho GTPase activating protein 32	2.49
Arhgap39	Rho GTPase activating protein 39	1.88
Arhgef11	Rho guanine nucleotide exchange factor (GEF) 11	1.99
Arhgef6	Rac/Cdc42 guanine nucleotide exchange factor (GEF) 6	2.97
Arhgef9	Cdc42 guanine nucleotide exchange factor (GEF) 9	2.02
Arl4c-ps1	ADP-ribosylation factor-like 4C, pseudogene 1	3.71
Arl5c	ADP-ribosylation factor-like 5C	2.67
Armc12	armadillo repeat containing 12	2.17
Armcx2	armadillo repeat containing, X-linked 2	2.38
Arnt2	aryl hydrocarbon receptor nuclear translocator 2	1.95
Arntl2	aryl hydrocarbon receptor nuclear translocator-like 2	2.03
Art3	ADP-ribosyltransferase 3	3.73
Asah1	N-acylsphingosine amidohydrolase (acid ceramidase) 1	2.24
Asgr1	asialoglycoprotein receptor 1	6.35
Asic1	acid-sensing (proton-gated) ion channel 1	5.06
Asip	agouti signaling protein	2.00
Asns	asparagine synthetase (glutamine-hydrolyzing)	2.35
Aspa	aspartoacylase	1.91

Astl	astacin-like metalloendopeptidase (M12 family)	6.14
Atcay	ataxia, cerebellar, Cayman type	1.79
Atf5	activating transcription factor 5	1.98
Atg16l1	autophagy related 16-like 1 (S. cerevisiae)	3.03
Atmin	ATM interactor	2.38
Atp10d	ATPase, class V, type 10D	2.15
Atp1b2	ATPase, Na+/K+ transporting, beta 2 polypeptide	3.09
Atp6ap1l	ATPase, H+ transporting, lysosomal accessory protein 1-like	4.21
Atp6v1a	ATPase, H+ transporting, lysosomal V1 subunit A	2.25
Atp6v1c1	ATPase, H+ transporting, lysosomal V1 subunit C1	1.89
Atp7b	ATPase, Cu++ transporting, beta polypeptide	1.85
Atp8b3	ATPase, aminophospholipid transporter, class I, type 8B, member 3	1.76
Atxn1	ataxin 1	1.98
Axin2	axin 2	3.04
B3gnt1	UDP-GlcNAc:betaGal beta-1,3-N-acetylglucosaminyltransferase 1	1.83
B4galnt1	beta-1,4-N-acetyl-galactosaminyl transferase 1	1.94
B4galt4	UDP-Gal:betaGlcNAc beta 1,4-galactosyltransferase, polypeptide 4	2.52
Bach1	BTB and CNC homology 1, basic leucine zipper transcription factor 1	2.56
Bai2	brain-specific angiogenesis inhibitor 2	3.12
Batf	basic leucine zipper transcription factor, ATF-like	2.12
Bbc3	Bcl-2 binding component 3	1.82
Bbs4	Bardet-Biedl syndrome 4	1.83
Bbx	bobby sox homolog (Drosophila)	1.97
Bcam	basal cell adhesion molecule (Lutheran blood group)	1.91
Bcan	brevican	7.82
Bcas1	breast carcinoma amplified sequence 1	4.83
Bcl11a	B-cell CLL/lymphoma 11A (zinc finger protein)	1.94
Bcl2	B-cell CLL/lymphoma 2	2.05
Bcl2l10	BCL2-like 10 (apoptosis facilitator)	1.95
Bcl6b	B-cell CLL/lymphoma 6, member B	1.96
Bdh2	3-hydroxybutyrate dehydrogenase, type 2	2.72
Bdkrb2	bradykinin receptor B2	2.54
Bdnf	brain-derived neurotrophic factor	1.80
Bex2	brain expressed X-linked 2	3.11
Bex4	brain expressed, X-linked 4	2.74
Bgn	biglycan	1.93
Bhlha15	basic helix-loop-helix family, member a15	2.41
Bhmt2	betaine-homocysteine S-methyltransferase 2	2.31
Bik	BCL2-interacting killer (apoptosis-inducing)	2.23
Bin2	bridging integrator 2	2.63
Birc7	baculoviral IAP repeat-containing 7	5.04
Blnk	B-cell linker	8.16
Bloc1s3	biogenesis of lysosomal organelles complex-1, subunit 3	1.82
Bmp7	bone morphogenetic protein 7	1.83
Bmyc	brain expressed myelocytomatosis oncogene	3.68

Bnc2	basonuclin 2	1.78
Bpifb6	BPI fold containing family B, member 6	21.63
Brsk2	BR serine/threonine kinase 2	1.94
Bsn	bassoon (presynaptic cytomatrix protein)	4.42
Btbd10	BTB (POZ) domain containing 10	1.75
Btbd17	BTB (POZ) domain containing 17	1.84
Btg3	BTG family, member 3	1.88
Bucs1	butyryl Coenzyme A synthetase 1	1.98
C1galt1	core 1 synthase, glycoprotein-N-acetylgalactosamine 3-beta-galactosyltransferase, 1	1.79
C1qtnf1	C1q and tumor necrosis factor related protein 1	6.70
C1qtnf4	C1q and tumor necrosis factor related protein 4	4.38
C1qtnf6	C1q and tumor necrosis factor related protein 6	2.86
C4bpb	complement component 4 binding protein, beta	3.16
Ca2	carbonic anhydrase 2	2.09
Cables2	Cdk5 and Abl enzyme substrate 2	2.49
Cabp1	calcium binding protein 1	2.19
Cacfd1	calcium channel flower domain containing 1	2.80
Cacna1h	calcium channel, voltage-dependent, T type, alpha 1H subunit	2.16
Cacna2d1	calcium channel, voltage-dependent, alpha2/delta subunit 1	5.59
Cacnb4	calcium channel, voltage-dependent, beta 4 subunit	2.37
Calb2	calbindin 2	5.82
Caly	calcyon neuron-specific vesicular protein	3.48
Camkk1	calcium/calmodulin-dependent protein kinase kinase 1, alpha	3.00
Camp	cathelicidin antimicrobial peptide	1.89
Camsap3	calmodulin regulated spectrin-associated protein family, member 3	2.66
Camta2	calmodulin binding transcription activator 2	2.87
Capn8	calpain 8	11.25
Capns2	calpain, small subunit 2	2.59
Car15	carbonic anhydrase 15	2.00
Car6	carbonic anhydrase 6	1.82
Car6	carbonic anhydrase 6	1.85
Caskin1	CASK interacting protein 1	2.01
Casp4	caspase 4, apoptosis-related cysteine peptidase	2.23
Catsper4	cation channel, sperm associated 4	2.27
Cblc	Cbl proto-oncogene, E3 ubiquitin protein ligase C	2.19
Cbp	Csk binding protein	1.94
Cbr3	carbonyl reductase 3	2.02
Cby3	chibby homolog 3 (Drosophila)	1.84
Ccdc116	coiled-coil domain containing 116	2.50
Ccdc126	coiled-coil domain containing 126	1.88
Ccdc15	coiled-coil domain containing 15	3.79
Ccdc162	coiled-coil domain containing 162	2.07
Ccdc175	coiled-coil domain containing 175	2.03
Ccdc63	coiled-coil domain containing 63	2.24

Ccdc79	coiled-coil domain containing 79	2.05
Ccdc88b	coiled-coil domain containing 88B	2.25
Ccdc92	coiled-coil domain containing 92	1.88
Cck	cholecystokinin	9.50
Ccl17	chemokine (C-C motif) ligand 17	1.92
Ccl19	chemokine (C-C motif) ligand 19	3.63
Ccl5	chemokine (C-C motif) ligand 5	4.27
Ccnb1ip1	cyclin B1 interacting protein 1, E3 ubiquitin protein ligase	4.59
Ccnb3	cyclin B3	2.42
Ccnd2	cyclin D2	3.87
Ccr1l1	chemokine (C-C motif) receptor 1-like 1	1.79
Ccrl2	chemokine (C-C motif) receptor-like 2	1.88
Ccrn4l	CCR4 carbon catabolite repression 4-like (S. cerevisiae)	2.43
Cd101	CD101 molecule	1.74
Cd177	CD177 molecule	2.10
Cd247	Cd247 molecule	3.91
Cd248	CD248 molecule, endosialin	1.81
Cd300a	Cd300a molecule	2.23
Cd40	CD40 molecule, TNF receptor superfamily member 5	3.77
Cd55	Cd55 molecule	2.11
Cd68	Cd68 molecule	2.41
Cd74	Cd74 molecule, major histocompatibility complex, class II invariant chain	9.49
Cd83	CD83 molecule	1.77
Cdc14a	CDC14 cell division cycle 14 homolog A (S. cerevisiae)	2.13
Cdc14b	CDC14 cell division cycle 14 homolog B (S. cerevisiae)	3.03
Cdc42bpa	CDC42 binding protein kinase alpha	1.78
Cdc42bpg	CDC42 binding protein kinase gamma (DMPK-like)	2.20
Cdca7l	cell division cycle associated 7 like	5.14
Cdh17	cadherin 17	3.66
Cdh23	cadherin-related 23	2.41
Cdhr5	cadherin-related family member 5	1.95
Cdk12	cyclin-dependent kinase 12	2.01
Cdk19	cyclin-dependent kinase 19	2.02
Cdkn1a	cyclin-dependent kinase inhibitor 1A	7.46
Cdnf	cerebral dopamine neurotrophic factor	1.97
Cds1	CDP-diacylglycerol synthase 1	3.12
Cdyl2	chromodomain protein, Y-like 2	2.36
Cebpa	CCAAT/enhancer binding protein (C/EBP), alpha	2.64
Celf4	CUGBP, Elav-like family member 4	5.21
Celf5	CUGBP, Elav-like family member 5	2.02
Cep97	centrosomal protein 97	1.76
Cers1	ceramide synthase 1	2.28
Cers1	ceramide synthase 1	2.64
Cers6	ceramide synthase 6	2.37
Cers6	ceramide synthase 6	2.44

Ces1a	carboxylesterase 1A	3.74
Cfd	complement factor D (adipsin)	3.52
Cgref1	cell growth regulator with EF hand domain 1	3.00
Ch25h	cholesterol 25-hydroxylase	2.42
Chac1	ChaC, cation transport regulator homolog 1 (E. coli)	2.05
Chd2	chromodomain helicase DNA binding protein 2	2.23
Chgb	chromogranin B (secretogranin 1)	2.14
Chit1	chitinase 1 (chitotriosidase)	3.87
Chml	choroideremia-like (Rab escort protein 2)	2.80
Chmp2b	charged multivesicular body protein 2B	1.89
Chrd	chordin	2.07
Chrna5	cholinergic receptor, nicotinic, alpha 5 (neuronal)	1.97
Chrnd	cholinergic receptor, nicotinic, delta (muscle)	2.44
Chst1	carbohydrate (keratan sulfate Gal-6) sulfotransferase 1	2.94
Chst10	carbohydrate sulfotransferase 10	12.78
Chst13	carbohydrate (chondroitin 4) sulfotransferase 13	3.47
Chst5	carbohydrate (N-acetylglucosamine 6-O) sulfotransferase 5	2.83
Chst8	carbohydrate (N-acetylgalactosamine 4-0) sulfotransferase 8	3.16
Chsy1	chondroitin sulfate synthase 1	1.78
Chuk	conserved helix-loop-helix ubiquitous kinase	1.82
Cidea	cell death-inducing DFFA-like effector a	2.28
Ciita	class II, major histocompatibility complex, transactivator	4.85
Ckm	creatine kinase, muscle	2.51
Ckmt2	creatine kinase, mitochondrial 2, sarcomeric	2.32
Clcn7	chloride channel, voltage-sensitive 7	1.89
Clcnkb	chloride channel, voltage-sensitive Kb	1.80
Cldn4	claudin 4	11.68
Clec12a	C-type lectin domain family 12, member A	3.09
Clec4a1	C-type lectin domain family 4, member A1	2.01
Clgn	calmegin	2.23
Clic5	chloride intracellular channel 5	5.93
Cln3	ceroid-lipofuscinosis, neuronal 3	1.81
Cln5	ceroid-lipofuscinosis, neuronal 5	2.46
Clock	clock circadian regulator	2.16
Clpsl2	colipase-like 2	1.83
Clu	clusterin	2.64
Cmbl	carboxymethylenebutenolidase homolog (Pseudomonas)	2.49
Cmpk2	cytidine monophosphate (UMP-CMP) kinase 2, mitochondrial	5.60
Cmtm2a	CKLF-like MARVEL transmembrane domain containing 2A	3.13
Cmtm4	CKLF-like MARVEL transmembrane domain containing 4	1.97
Cndp1	carnosine dipeptidase 1 (metallopeptidase M20 family)	3.25
Cnksr1	connector enhancer of kinase suppressor of Ras 1	2.23
Cnnm2	cyclin M2	1.91
Cnst	consortin, connexin sorting protein	1.95
Cntn2	contactin 2 (axonal)	2.00

Col11a2	collagen, type XI, alpha 2	3.41
Col12a1	collagen, type XII, alpha 1	5.27
Col14a1	collagen, type XIV, alpha 1	1.88
Col15a1	collagen, type XV, alpha 1	3.63
Col17a1	collagen, type XVII, alpha 1	3.13
Col1a1	collagen, type I, alpha 1	2.06
Col20a1	collagen, type XX, alpha 1	11.56
Col27a1	collagen, type XXVII, alpha 1	7.39
Col5a2	collagen, type V, alpha 2	1.92
Col5a3	collagen, type V, alpha 3	2.12
Col6a1	collagen, type VI, alpha 1	8.01
Col6a2	collagen, type VI, alpha 2	2.35
Col7a1	collagen, type VII, alpha 1	2.43
Col8a1	collagen, type VIII, alpha 1	2.35
Copg2	coatomer protein complex, subunit gamma 2	1.88
Coro6	coronin 6	2.98
Cort	cortistatin	3.04
Cox6a2	cytochrome c oxidase subunit VIa polypeptide 2	11.15
Cpa1	carboxypeptidase A1 (pancreatic)	1.87
Cpamd8	C3 and PZP-like, alpha-2-macroglobulin domain containing 8	5.29
Cpe	carboxypeptidase E	2.68
Cpeb1	cytoplasmic polyadenylation element binding protein 1	2.97
Cpeb4	cytoplasmic polyadenylation element binding protein 4	2.00
Cplx2	complexin 2	2.04
Cpm	carboxypeptidase M	3.35
Cpne6	copine VI (neuronal)	2.06
Cpne7	copine VII	4.91
Cpne9	copine family member IX	2.20
Cpt1b	carnitine palmitoyltransferase 1b, muscle	2.06
Cpz	carboxypeptidase Z	3.09
Crabp1	cellular retinoic acid binding protein 1	1.91
Crb3	crumbs homolog 3 (Drosophila)	2.24
Crcp	CGRP receptor component	2.30
Creb3l3	cAMP responsive element binding protein 3-like 3	7.03
Creb5	cAMP responsive element binding protein 5	1.93
Crebl2	cAMP responsive element binding protein-like 2	2.63
Creg1	cellular repressor of E1A-stimulated genes 1	3.25
Crip1	cysteine-rich protein 1 (intestinal)	2.10
Crlf1	cytokine receptor-like factor 1	1.95
Crtc3	CREB regulated transcription coactivator 3	1.91
Cryba4	crystallin, beta A4	3.43
Crybb2	crystallin, beta B2	10.79
Crybg3	beta-gamma crystallin domain containing 3	4.14
Cryl1	crystallin, lambda 1	2.00
Crym	crystallin, mu	2.88

Cryz	crystallin, zeta (quinone reductase)	1.82
Csdc2	cold shock domain containing C2, RNA binding	2.78
Cspg4	chondroitin sulfate proteoglycan 4	3.07
Csrnp1	cysteine-serine-rich nuclear protein 1	1.93
Csrnp2	cysteine-serine-rich nuclear protein 2	1.78
Csrp2	cysteine and glycine-rich protein 2	1.80
Csrp3	cysteine and glycine-rich protein 3 (cardiac LIM protein)	3.74
Cst6	cystatin E/M	2.50
Cst9l	cystatin 9-like	3.92
Ctdp1	CTD (carboxy-terminal domain, RNA polymerase II, polypeptide A) phosphatase, subunit 1	1.78
Ctgf	connective tissue growth factor	2.04
Ctla2a	cytotoxic T lymphocyte-associated protein 2 alpha	2.90
Ctnna2	catenin (cadherin associated protein), alpha 2	3.12
Ctrb1	chymotrypsinogen B1	2.47
Ctss	cathepsin S	2.43
Ctsw	cathepsin W	5.41
Ctxn3	cortexin 3	2.78
Cul9	cullin 9	2.05
Cxcl10	chemokine (C-X-C motif) ligand 10	2.74
Cxcl14	chemokine (C-X-C motif) ligand 14	6.38
Cxcr3	chemokine (C-X-C motif) receptor 3	2.32
Cyfip2	cytoplasmic FMR1 interacting protein 2	2.19
Cyfip2	cytoplasmic FMR1 interacting protein 2	2.48
Cyp11a1	cytochrome P450, family 11, subfamily a, polypeptide 1	3.61
Cyp27a1	cytochrome P450, family 27, subfamily a, polypeptide 1	2.23
Cyp27b1	cytochrome P450, family 27, subfamily b, polypeptide 1	2.15
Cyp2b3	cytochrome P450, family 2, subfamily b, polypeptide 3	2.92
Cyp2b3	cytochrome P450, family 2, subfamily b, polypeptide 3	1.96
Cyp2c7	cytochrome P450, family 2, subfamily c, polypeptide 7	2.23
Cyp2d1	cytochrome P450, family 2, subfamily d, polypeptide 1	3.22
Cyp2d5	cytochrome P450, family 2, subfamily d, polypeptide 5	2.59
Cyp2f4	cytochrome P450, family 2, subfamily f, polypeptide 4	3.25
Cyp2j4	cytochrome P450, family 2, subfamily j, polypeptide 4	4.45
Cyp2j4	cytochrome P450, family 2, subfamily j, polypeptide 4	2.73
Cyp2s1	cytochrome P450, family 2, subfamily s, polypeptide 1	2.61
Cyp3a9	cytochrome P450, family 3, subfamily a, polypeptide 9	2.92
Cyp4f1	cytochrome P450, family 4, subfamily f, polypeptide 1	7.59
Cyp4f5	cytochrome P450, family 4, subfamily f, polypeptide 5	4.13
Dapk1	death associated protein kinase 1	2.69
Dcaf5	DDB1 and CUL4 associated factor 5	1.94
Dclk2	doublecortin-like kinase 2	1.97
Dclk3	doublecortin-like kinase 3	2.65
Dcp1b	DCP1 decapping enzyme homolog b (S. cerevisiae)	3.65
Ddc	dopa decarboxylase (aromatic L-amino acid decarboxylase)	3.73

Ddit3	DNA-damage inducible transcript 3	3.35
Ddx60	DEAD (Asp-Glu-Ala-Asp) box polypeptide 60	4.57
Defb29	defensin beta 29	3.64
Defb36	defensin beta 36	2.19
Defb42	defensin beta 42	1.97
Dennd2d	DENN/MADD domain containing 2D	3.91
Deptor	DEP domain containing MTOR-interacting protein	2.41
Dfnb31	deafness, autosomal recessive 31	1.88
Dgkd	diacylglycerol kinase, delta	1.81
Dhh	desert hedgehog	2.42
Dhh	desert hedgehog	3.47
Dhrs2	dehydrogenase/reductase (SDR family) member 2	3.69
Dlg2	discs, large homolog 2 (Drosophila)	2.28
Dlg4	discs, large homolog 4 (Drosophila)	1.83
Dll1	delta-like 1 (Drosophila)	3.94
Dll3	delta-like 3 (Drosophila)	2.67
Dll3	delta-like 3 (Drosophila)	2.26
Dlx3	distal-less homeobox 3	3.28
Dmbx1	diencephalon/mesencephalon homeobox 1	8.10
Dmd	dystrophin	2.18
Dmkn	dermokine	4.07
Dna2	DNA replication helicase 2 homolog (yeast)	2.01
Dnah12	dynein, axonemal, heavy chain 12	11.35
Dnajb9	DnaJ (Hsp40) homolog, subfamily B, member 9	1.94
Dnajc12	DnaJ (Hsp40) homolog, subfamily C, member 12	5.54
Dnajc6	DnaJ (Hsp40) homolog, subfamily C, member 6	3.75
Dnm3	dynamin 3	2.34
Dnm3	dynamin 3	2.20
Dnmbp	dynamin binding protein	2.24
Doc2a	double C2-like domains, alpha	6.31
Dock9	dedicator of cytokinesis 9	1.82
Dos	downstream of Stk11	1.86
Dpep2	dipeptidase 2	3.72
Dpysl3	dihydropyrimidinase-like 3	2.32
Dqx1	DEAQ box RNA-dependent ATPase 1	2.27
Dsp	desmoplakin	1.91
Dtx3	deltex homolog 3 (Drosophila)	1.80
Duox1	dual oxidase 1	3.66
Duox2	dual oxidase 2	4.86
Dusp10	dual specificity phosphatase 10	2.21
Dusp13	dual specificity phosphatase 13	6.01
Dusp2	dual specificity phosphatase 2	2.36
Dusp26	dual specificity phosphatase 26 (putative)	1.97
Dusp4	dual specificity phosphatase 4	2.17
Dusp5	dual specificity phosphatase 5	2.65

Dusp6	dual specificity phosphatase 6	1.96
Dusp9	dual specificity phosphatase 9	2.14
Dydc1	DPY30 domain containing 1	1.82
Dynll2	dynein light chain LC8-type 2	1.87
Dyrk2	dual-specificity tyrosine-(Y)-phosphorylation regulated kinase 2	1.89
Dyrk3	dual-specificity tyrosine-(Y)-phosphorylation regulated kinase 3	2.29
Dyx1c1	dyslexia susceptibility 1 candidate 1 homolog (human)	1.78
Eaf2	ELL associated factor 2	7.06
Ear11	eosinophil-associated, ribonuclease A family, member 11	3.49
Ece1	endothelin converting enzyme 1	1.78
Ecm1	extracellular matrix protein 1	3.09
Eddm3b	epididymal protein 3B	3.10
Eef2k	eukaryotic elongation factor-2 kinase	2.19
Eef2k	eukaryotic elongation factor-2 kinase	2.32
Efcab6	EF-hand calcium binding domain 6	3.34
Efcab9	EF-hand calcium binding domain 9	2.01
Efha2	EF hand domain family, member A2	1.82
Efhd1	EF-hand domain family, member D1	3.84
Efnb1	ephrin B1	1.93
Efr3b	EFR3 homolog B (S. cerevisiae)	1.91
Egr3	early growth response 3	1.97
Ei24	etoposide induced 2.4	1.95
Eid2	EP300 interacting inhibitor of differentiation 2	1.86
Eif4a2	eukaryotic translation initiation factor 4A2	1.96
Eif4e3	eukaryotic translation initiation factor 4E family member 3	1.93
Eif4e3	eukaryotic translation initiation factor 4E family member 3	1.79
Eif4ebp1	eukaryotic translation initiation factor 4E binding protein 1	1.81
Elmsan1	ELM2 and Myb/SANT-like domain containing 1	2.83
Eln	elastin	3.26
Elovl2	ELOVL fatty acid elongase 2	3.02
Emid1	EMI domain containing 1	2.92
Emilin3	elastin microfibril interfacer 3	1.86
Eml5	echinoderm microtubule associated protein like 5	2.80
Eml5	echinoderm microtubule associated protein like 5	3.18
Eno3	enolase 3, beta, muscle	1.81
Enpp1	ectonucleotide pyrophosphatase/phosphodiesterase 1	4.46
Enpp2	ectonucleotide pyrophosphatase/phosphodiesterase 2	5.37
Enpp3	ectonucleotide pyrophosphatase/phosphodiesterase 3	2.69
Enthd1	ENTH domain containing 1	6.06
Entpd1	ectonucleoside triphosphate diphosphohydrolase 1	3.93
Entpd2	ectonucleoside triphosphate diphosphohydrolase 2	2.25
Entpd2	ectonucleoside triphosphate diphosphohydrolase 2	2.06
Entpd2	ectonucleoside triphosphate diphosphohydrolase 2	2.00
Entpd3	ectonucleoside triphosphate diphosphohydrolase 3	2.41
Entpd6	ectonucleoside triphosphate diphosphohydrolase 6	2.03

Epb41l1	erythrocyte membrane protein band 4.1-like 1	1.79
Epb49	erythrocyte membrane protein band 4.9 (dematin)	2.00
Epdr1	ependymin related protein 1 (zebrafish)	3.14
Epha2	Eph receptor A2	2.42
Epha8	Eph receptor A8	6.02
Ephb2	Eph receptor B2	2.05
Ephb6	Eph receptor B6	2.47
Eps8l1	EPS8-like 1	4.22
Ero1lb	ERO1-like beta (S. cerevisiae)	1.75
Espn	espin	5.45
Espn	espin	4.04
Etl4	enhancer trap locus 4	2.07
Ets1	v-ets erythroblastosis virus E26 oncogene homolog 1 (avian)	2.00
Ets2	v-ets erythroblastosis virus E26 oncogene homolog 2 (avian)	1.82
Ets2	v-ets erythroblastosis virus E26 oncogene homolog 2 (avian)	2.28
Etv2	ets variant 2	4.27
Etv3	ets variant 3	3.32
Etv4	ets variant 4	2.54
Etv5	ets variant 5	4.34
Etv5	ets variant 5	3.83
Eva1c	eva-1 homolog C	3.14
Evpl	envoplakin	2.14
Exd1	exonuclease 3'-5' domain containing 1	3.30
Exoc3l4	exocyst complex component 3-like 4	1.81
Exoc6b	exocyst complex component 6B	2.45
Extl1	exostoses (multiple)-like 1	2.20
Ezh1	enhancer of zeste homolog 1 (Drosophila)	1.78
F10	coagulation factor X	3.30
F12	coagulation factor XII (Hageman factor)	4.18
F2rl1	coagulation factor II (thrombin) receptor-like 1	2.02
F3	coagulation factor III (thromboplastin, tissue factor)	2.43
Faah	fatty acid amide hydrolase	1.98
Faim2	Fas apoptotic inhibitory molecule 2	1.83
Fam101a	family with sequence similarity 101, member A	5.48
Fam102b	family with sequence similarity 102, member B	3.86
Fam105a	family with sequence similarity 105, member A	3.15
Fam110b	family with sequence similarity 110, member B	1.93
Fam118a	family with sequence similarity 118, member A	1.88
Fam124a	family with sequence similarity 124A	3.19
Fam126b	family with sequence similarity 126, member B	2.76
Fam131b	family with sequence similarity 131, member B	1.87
Fam134b	family with sequence similarity 134, member B	1.85
Fam134b	family with sequence similarity 134, member B	2.39
Fam135a	family with sequence similarity 135, member A	1.86
Fam13c	family with sequence similarity 13, member C	2.34

Fam151a	family with sequence similarity 151, member A	4.42
Fam160b2	family with sequence similarity 160, member B2	2.26
Fam161b	family with sequence similarity 161, member B	1.78
Fam167a	family with sequence similarity 167, member A	3.36
Fam171b	family with sequence similarity 171, member B	6.12
Fam186b	family with sequence similarity 186, member B	2.09
Fam186b	family with sequence similarity 186, member B	1.84
Fam210b	family with sequence similarity 210, member B	2.04
Fam214a	family with sequence similarity 214, member A	2.14
Fam219a	family with sequence similarity 219, member A	3.19
Fam219a	family with sequence similarity 219, member A	2.98
Fam220a	family with sequence similarity 220, member A	3.20
Fam221a	family with sequence similarity 221, member A	2.75
Fam227a	family with sequence similarity 227, member A	6.41
Fam227b	family with sequence similarity 227, member B	2.16
Fam26e	family with sequence similarity 26, member E	3.81
Fam26f	family with sequence similarity 26, member F	4.74
Fam46b	family with sequence similarity 46, member B	1.99
Fam46c	family with sequence similarity 46, member C	2.28
Fam49a	family with sequence similarity 49, member A	1.80
Fam57b	family with sequence similarity 57, member B	5.02
Fam60a	family with sequence similarity 60, member A	2.12
Fam65b	family with sequence similarity 65, member B	2.22
Fam69b	family with sequence similarity 69, member B	2.09
Fam71e1	family with sequence similarity 71, member E1	1.78
Fam83h	family with sequence similarity 83, member H	2.32
Fam90a1-ps2	family with sequence similarity 90, member A1-pseudogene 2	2.02
Fam90a1-ps2	family with sequence similarity 90, member A1-pseudogene 2	1.74
Fastkd1	FAST kinase domains 1	2.23
Fbln1	fibulin 1	1.93
Fbln2	fibulin 2	2.31
Fbn1	fibrillin 1	1.85
Fbp2	fructose-1,6-bisphosphatase 2	9.47
Fbxl20	F-box and leucine-rich repeat protein 20	1.94
Fbxl4	F-box and leucine-rich repeat protein 4	3.21
Fbxo23	F-box only protein 23	2.09
Fbxo32	F-box protein 32	2.27
Fbxo43	F-box protein 43	8.06
Fcer1g	Fc fragment of IgE, high affinity I, receptor for; gamma polypeptide	2.02
Fcgr2b	Fc fragment of IgG, low affinity IIb, receptor (CD32)	2.62
Fcgr3a	Fc fragment of IgG, low affinity IIIa, receptor	7.12
Fcho1	FCH domain only 1	2.22
Fcrl6	Fc receptor-like 6	1.92

Fech	ferrochelatase	2.15
Fem1b	fem-1 homolog b (C. elegans)	2.23
Fer1l4	fer-1-like 4 (C. elegans)	3.72
Fermt1	fermitin family member 1	2.38
Fetub	fetuin B	2.58
Fez1	fasciculation and elongation protein zeta 1 (zygin I)	4.86
Fgf21	fibroblast growth factor 21	2.60
Fgfr4	fibroblast growth factor receptor 4	2.04
Fgg	fibrinogen gamma chain	6.08
Fibcd1	fibrinogen C domain containing 1	10.07
Flcn	folliculin	2.53
Flrt1	fibronectin leucine rich transmembrane protein 1	4.20
Flrt2	fibronectin leucine rich transmembrane protein 2	2.84
Flt3	fms-related tyrosine kinase 3	2.57
Fmnl1	formin-like 1	1.92
Fmo2	flavin containing monooxygenase 2	3.89
Fmo2	flavin containing monooxygenase 2	3.00
Fmo9p	flavin containing monooxygenase 9 pseudogene	2.27
Fmod	fibromodulin	2.98
Fn3k	fructosamine 3 kinase	4.71
Fnbp1	formin binding protein 1	2.24
Fnbp1l	formin binding protein 1-like	2.02
Fntb	farnesyltransferase, CAAX box, beta	2.04
Fosl1	fos-like antigen 1	6.77
Foxa1	forkhead box A1	2.00
Foxe1	forkhead box E1 (thyroid transcription factor 2)	2.76
Foxj2	forkhead box J2	1.79
Foxk1	forkhead box K1	2.07
Foxo6	forkhead box O6	2.27
Fras1	Fraser syndrome 1	2.08
Frem3	FRAS1 related extracellular matrix 3	5.04
Frmd5	FERM domain containing 5	3.22
Frmpd1	FERM and PDZ domain containing 1	3.66
Fry	furry homolog (Drosophila)	3.91
Fst	follistatin	2.45
Fstl3	follistatin-like 3 (secreted glycoprotein)	2.23
Fstl3	follistatin-like 3 (secreted glycoprotein)	2.27
Ftcd	formiminotransferase cyclodeaminase	3.65
Fut1	fucosyltransferase 1	5.97
Fut7	fucosyltransferase 7 (alpha (1,3) fucosyltransferase)	1.82
Fxyd5	FXYD domain-containing ion transport regulator 5	2.04
Fxyd6	FXYD domain-containing ion transport regulator 6	16.54
Fxyd7	FXYD domain-containing ion transport regulator 7	2.57
Fzd4	frizzled family receptor 4	2.23
Fzd9	frizzled family receptor 9	2.84

G0s2	G0/G1switch 2	3.22
Gaa	glucosidase, alpha, acid	2.14
Gabarapl1	GABA(A) receptor-associated protein like 1	2.52
Gabbr1	gamma-aminobutyric acid (GABA) B receptor 1	2.15
Gabbr1	gamma-aminobutyric acid (GABA) B receptor 1	2.29
Gabbr2	gamma-aminobutyric acid (GABA) B receptor 2	2.80
Gal3st4	galactose-3-O-sulfotransferase 4	2.28
Galm	galactose mutarotase (aldose 1-epimerase)	3.14
Galntl1	UDP-N-acetyl-alpha-D-galactosamine:polypeptide N-acetylgalactosaminyltransferase-like 1	3.00
Galp	galanin-like peptide	1.96
Gas2l3	growth arrest-specific 2 like 3	1.85
Gas5	growth arrest specific 5	2.12
Gas6	growth arrest specific 6	1.89
Gata3	GATA binding protein 3	2.20
Gata4	GATA binding protein 4	1.98
Gbp5	guanylate binding protein 5	3.64
Gbp5	guanylate binding protein 5	2.82
Gchfr	GTP cyclohydrolase I feedback regulator	1.99
Gcnt2	glucosaminyl (N-acetyl) transferase 2, I-branching enzyme	2.32
Gdf15	growth differentiation factor 15	2.18
Ggh	gamma-glutamyl hydrolase (conjugase, folylpolygammaglutamyl hydrolase)	2.31
Ggt5	gamma-glutamyltransferase 5	3.12
Ghrh	growth hormone releasing hormone	2.13
Gigyf1	GRB10 interacting GYF protein 1	1.97
Gimap5	GTPase, IMAP family member 5	3.07
Gja4	gap junction protein, alpha 4	3.30
Gja8	gap junction membrane channel protein alpha 8	2.46
Gjb5	gap junction protein, beta 5	5.59
Gkap1	G kinase anchoring protein 1	2.11
Gli1	GLI family zinc finger 1	3.46
Glipr1	GLI pathogenesis-related 1	5.46
Glp1r	glucagon-like peptide 1 receptor	1.96
Glt8d2	glycosyltransferase 8 domain containing 2	2.53
Gna13	guanine nucleotide binding protein (G protein), alpha 13	1.96
Gnao1	guanine nucleotide binding protein (G protein), alpha activating activity polypeptide O	2.17
Gnao1	guanine nucleotide binding protein (G protein), alpha activating activity polypeptide O	2.10
Gnaq	guanine nucleotide binding protein (G protein), q polypeptide	2.20
Gnat1	guanine nucleotide binding protein (G protein), alpha transducing activity polypeptide 1	3.96
Gnat2	guanine nucleotide binding protein (G protein), alpha transducing activity polypeptide 2	1.80
Gnaz	guanine nucleotide binding protein (G protein), alpha z polypeptide	2.52
Gng3	guanine nucleotide binding protein (G protein), gamma 3	1.90
Gng8	guanine nucleotide binding protein (G protein), gamma 8	2.76

Gngt2	guanine nucleotide binding protein (G protein), gamma transducing activity polypeptide 2	1.87
Gnpda1	glucosamine-6-phosphate deaminase 1	1.95
Golm1	golgi membrane protein 1	1.97
Gpc3	glypican 3	1.98
Gpha2	glycoprotein hormone alpha 2	2.65
Gpld1	glycosylphosphatidylinositol specific phospholipase D1	3.05
Gpm6a	glycoprotein m6a	3.72
Gpnmb	glycoprotein (transmembrane) nmb	14.07
Gpr146	G protein-coupled receptor 146	1.75
Gpr4	G protein-coupled receptor 4	2.38
Gpr75	G protein-coupled receptor 75	1.76
Gpr77	G protein-coupled receptor 77	1.86
Gpr83	G protein-coupled receptor 83	3.25
Gpr85	G protein-coupled receptor 85	3.97
Gprc5b	G protein-coupled receptor, family C, group 5, member B	5.03
Gprc5d	G protein-coupled receptor, family C, group 5, member D	1.77
Gprin3	GPRIN family member 3	2.19
Gpx2	glutathione peroxidase 2	1.87
Gramd1a	GRAM domain containing 1A	1.82
Grb7	growth factor receptor bound protein 7	2.22
Grid2	glutamate receptor, ionotropic, delta 2	1.78
Grid2ip	glutamate receptor, ionotropic, delta 2 (Grid2) interacting protein	2.67
Grifin	galectin-related inter-fiber protein	2.21
Grm4	glutamate receptor, metabotropic 4	6.16
Grm4	glutamate receptor, metabotropic 4	3.85
Grp	gastrin releasing peptide	4.61
Gsta2	glutathione S-transferase alpha 2	3.84
Gsta3	glutathione S-transferase A3	15.70
Gsta4	glutathione S-transferase alpha 4	3.29
Gsta5	glutathione S-transferase Yc2 subunit	5.13
Gsta5	glutathione S-transferase alpha 5	6.00
Gstm1	glutathione S-transferase mu 1	1.84
Gstm7	glutathione S-transferase, mu 7	2.17
Gstp1	glutathione S-transferase pi 1	2.03
Gulp1	GULP, engulfment adaptor PTB domain containing 1	2.15
Gyg1	glycogenin 1	2.21
Gzmc	granzyme C	2.73
Gzmc	granzyme C	1.84
H19	H19, imprinted maternally expressed transcript (non-protein coding)	4.42
Hadh	hydroxyacyl-CoA dehydrogenase	1.90
Hal	histidine ammonia lyase	2.79
Hap1	huntingtin-associated protein 1	2.04
Hapln2	hyaluronan and proteoglycan link protein 2	3.61
Hapln3	hyaluronan and proteoglycan link protein 3	5.94

Hbegf	heparin-binding EGF-like growth factor	1.92
Hbz	hemoglobin, zeta	1.98
Hck	hemopoietic cell kinase	5.44
Hcst	hematopoietic cell signal transducer	2.34
Hdgfrp3	hepatoma-derived growth factor, related protein 3	1.78
Helb	helicase (DNA) B	2.11
Herc6	HECT and RLD domain containing E3 ubiquitin protein ligase family member 6	3.70
Herpud1	homocysteine-inducible, endoplasmic reticulum stress-inducible, ubiquitin-like domain member 1	2.28
Hey1	hairy/enhancer-of-split related with YRPW motif 1	2.26
Hey2	hairy/enhancer-of-split related with YRPW motif 2	2.07
Hhex	hematopoietically expressed homeobox	1.94
Hk3	hexokinase 3 (white cell)	2.13
Hlxb9	homeobox gene HB9	3.06
Hmga1	high mobility group AT-hook 1	2.44
Hmgb1-ps2	high mobility group box 1, pseudogene 2	2.20
Hmgcs2	3-hydroxy-3-methylglutaryl-CoA synthase 2 (mitochondrial)	1.92
Hnrpll	heterogeneous nuclear ribonucleoprotein L-like	2.26
Hook1	hook homolog 1 (Drosophila)	1.89
Hook2	hook homolog 2 (Drosophila)	1.90
Hopx	HOP homeobox	4.17
Hoxb4	homeo box B4	2.03
Hoxc6	homeo box C6	2.41
Hoxd4	homeo box D4	1.94
Hp	haptoglobin	1.86
Hpcal1	hippocalcin-like 1	2.02
Hps4	Hermansky-Pudlak syndrome 4	1.86
Hrc	histidine rich calcium binding protein	3.30
Hrk	harakiri, BCL2 interacting protein (contains only BH3 domain)	13.02
Hs1bp3	HCLS1 binding protein 3	1.84
Hs2st1	heparan sulfate 2-O-sulfotransferase 1	1.83
Hs3st3a1	heparan sulfate (glucosamine) 3-O-sulfotransferase 3A1	3.80
Hs3st6	heparan sulfate (glucosamine) 3-O-sulfotransferase 6	3.58
Hs3st6	heparan sulfate (glucosamine) 3-O-sulfotransferase 6	3.62
Hsd11b1	hydroxysteroid 11-beta dehydrogenase 1	4.96
Hsd11b2	hydroxysteroid 11-beta dehydrogenase 2	2.36
Hsd17b1	hydroxysteroid (17-beta) dehydrogenase 1	2.52
Hsd3b1	hydroxy-delta-5-steroid dehydrogenase, 3 beta- and steroid delta-isomerase 1	2.71
Hsd3b6	hydroxy-delta-5-steroid dehydrogenase, 3 beta- and steroid delta-isomerase 6	2.27
Hspa1b	heat shock 70kD protein 1B (mapped)	1.78
Hspa4l	heat shock protein 4-like	2.83
Htatip2	HIV-1 Tat interactive protein 2	2.35
Ick	intestinal cell kinase	2.33
Ifi204	interferon activated gene 204	3.71
Ifi27l2b	interferon, alpha-inducible protein 27 like 2B	4.06

Ifi27l2b	interferon, alpha-inducible protein 27 like 2B	3.63
Ifit3	interferon-induced protein with tetratricopeptide repeats 3	7.66
Igbp1b	immunoglobulin (CD79A) binding protein 1b	2.13
Igf2	insulin-like growth factor 2	4.42
Igf2bp2	insulin-like growth factor 2 mRNA binding protein 2	2.24
Igf2r	insulin-like growth factor 2 receptor	1.91
Igfals	insulin-like growth factor binding protein, acid labile subunit	2.08
Igfbp2	insulin-like growth factor binding protein 2	2.49
Igfbp5	insulin-like growth factor binding protein 5	3.71
Igfbpl1	insulin-like growth factor binding protein-like 1	5.99
Igll1	immunoglobulin lambda-like polypeptide 1	2.73
Ihh	Indian hedgehog	2.65
Ikbip	IKBKB interacting protein	1.74
Ikzf5	IKAROS family zinc finger 5	2.12
Ikzf5	IKAROS family zinc finger 5	1.76
Il18	interleukin 18	2.80
Il18r1	interleukin 18 receptor 1	1.85
Il1r1	interleukin 1 receptor, type I	3.64
Il1r2	interleukin 1 receptor, type II	2.52
Il1rn	interleukin 1 receptor antagonist	3.10
Il34	interleukin 34	4.17
Il36b	interleukin 36, beta	2.72
Il36rn	interleukin 36 receptor antagonist	2.42
Il6r	interleukin 6 receptor	3.75
Impact	imprinted and ancient	2.44
Inhba	inhibin beta-A	2.39
Inhbb	inhibin beta-B	2.42
Insig2	insulin induced gene 2	1.86
Insr	insulin receptor	2.70
Insr	insulin receptor	2.80
Ints12	integrator complex subunit 12	1.77
Iqcb1	IQ motif containing B1	1.81
Irak2	interleukin-1 receptor-associated kinase 2	1.85
Irf4	interferon regulatory factor 4	2.03
Irf6	interferon regulatory factor 6	2.20
Irf8	interferon regulatory factor 8	2.03
Irgm	immunity-related GTPase family, M	3.04
Isg20	interferon stimulated exonuclease gene 20	3.74
Ism1	isthmin 1 homolog (zebrafish)	4.15
Isoc1	isochorismatase domain containing 1	2.18
Itga1	integrin, alpha 1	2.10
Itga10	integrin, alpha 10	1.82
Itga2b	integrin, alpha 2B	1.92
Itga6	integrin, alpha 6	2.12
Itga8	integrin, alpha 8	17.41

Itgb1bp2	integrin beta 1 binding protein 2	2.74
Itgb4	integrin, beta 4	1.82
Itgb7	integrin, beta 7	2.55
Itih4	inter-alpha-trypsin inhibitor heavy chain family, member 4	3.08
Itln1	intelectin 1 (galactofuranose binding)	7.35
Itpka	inositol-trisphosphate 3-kinase A	1.85
Jakmip1	janus kinase and microtubule interacting protein 1	5.75
Jakmip2	janus kinase and microtubule interacting protein 2	4.05
Kank3	KN motif and ankyrin repeat domains 3	2.17
Kat6a	K(lysine) acetyltransferase 6A	2.36
Kazald1	Kazal-type serine peptidase inhibitor domain 1	2.29
Kazn	kazrin, periplakin interacting protein	2.50
Kazn	kazrin, periplakin interacting protein	3.71
Kcna6	potassium voltage gated channel, shaker related subfamily, member 6	1.98
Kcnb1	potassium voltage gated channel, Shab-related subfamily, member 1	2.15
Kcnc3	potassium voltage gated channel, Shaw-related subfamily, member 3	2.72
Kcne2	potassium voltage-gated channel, Isk-related family, member 2	2.03
Kcng1	potassium voltage-gated channel, subfamily G, member 1	7.01
Kcng1	potassium voltage-gated channel, subfamily G, member 1	1.79
Kcnh1	potassium voltage-gated channel, subfamily H (eag-related), member 1	4.82
Kcnh2	potassium voltage-gated channel, subfamily H (eag-related), member 2	3.64
Kcnh3	potassium voltage-gated channel, subfamily H (eag-related), member 3	2.38
Kcnh4	potassium voltage-gated channel, subfamily H (eag-related), member 4	1.81
Kcnh6	potassium voltage-gated channel, subfamily H (eag-related), member 6	1.92
Kcnj12	potassium inwardly-rectifying channel, subfamily J, member 12	1.91
Kcnj4	potassium inwardly-rectifying channel, subfamily J, member 4	8.87
Kcnq1	potassium voltage-gated channel, KQT-like subfamily, member 1	1.82
Kcns1	potassium voltage-gated channel, delayed-rectifier, subfamily S, member 1	3.05
Kcnt1	potassium channel, subfamily T, member 1	2.60
Kctd13	potassium channel tetramerisation domain containing 13	2.31
Kctd19	potassium channel tetramerisation domain containing 19	1.84
Kctd3	potassium channel tetramerisation domain containing 3	1.90
Kdr	kinase insert domain receptor	4.30
Kdsr	3-ketodihydrosphingosine reductase	2.44
Kif13a	kinesin family member 13A	2.02
Kif26a	kinesin family member 26A	1.78
KIFC2	kinesin family member C2	2.32
Kit	v-kit Hardy-Zuckerman 4 feline sarcoma viral oncogene homolog	1.91
Kitlg	KIT ligand	6.12
Klf1	Kruppel-like factor 1 (erythroid)	2.26
Klf11	Kruppel-like factor 11	2.10
Klf14	Kruppel-like factor 14	7.24
Klf15	Kruppel-like factor 15	2.83
Klf2	Kruppel-like factor 2 (lung)	3.26
Klf3	Kruppel-like factor 3 (basic)	1.78

Klf4	Kruppel-like factor 4 (gut)	1.84
Klf5	Kruppel-like factor 5	2.00
Klf5	Kruppel-like factor 5	2.21
Klhl15	kelch-like 15 (Drosophila)	2.05
Klhl21	kelch-like 21 (Drosophila)	1.76
Klhl35	kelch-like 35 (Drosophila)	1.92
Klhl38	kelch-like 38 (Drosophila)	1.88
Klhl41	kelch-like 41 (Drosophila)	1.85
Klhl8	kelch-like 8 (Drosophila)	1.77
Klk13	kallikrein related-peptidase 13	5.54
Klk8	kallikrein related-peptidase 8	2.86
Klre1	killer cell lectin-like receptor, family E, member 1	3.06
Kptn	kaptin (actin binding protein)	1.79
Krt15	keratin 15	2.49
Krt23	keratin 23 (histone deacetylase inducible)	3.61
Krt25	keratin 25	2.42
Krt76	keratin 76	5.46
Krt81	keratin 81	2.44
Krt83	keratin 83	2.21
Krtap1-3	keratin associated protein 1-3	2.67
Krtap16-5	keratin associated protein 16-5	1.75
L1cam	L1 cell adhesion molecule	2.19
L3mbtl3	l(3)mbt-like 3 (Drosophila)	2.68
Lacc1	laccase (multicopper oxidoreductase) domain containing 1	3.77
Lamc2	laminin, gamma 2	3.62
Lats1	LATS, large tumor suppressor, homolog 1 (Drosophila)	2.88
Lcat	lecithin cholesterol acyltransferase	2.05
Lcor	ligand dependent nuclear receptor corepressor	2.18
Lcorl	ligand dependent nuclear receptor corepressor-like	1.76
Lcp2	lymphocyte cytosolic protein 2	3.05
Lct	lactase	1.88
Ldhb	lactate dehydrogenase B	45.99
Ldhc	lactate dehydrogenase C	2.71
Lgals3	lectin, galactoside-binding, soluble, 3	4.58
Lgals3bp	lectin, galactoside-binding, soluble, 3 binding protein	1.88
Lgals5	lectin, galactose binding, soluble 5	6.77
Lgals7	lectin, galactoside-binding, soluble, 7	3.63
Lgals9	lectin, galactoside-binding, soluble, 9	9.26
Lgi4	leucine-rich repeat LGI family, member 4	2.27
Lhb	luteinizing hormone beta	1.95
Lhx2	LIM homeobox 2	1.88
Lhx4	LIM homeobox 4	4.78
Lif	leukemia inhibitory factor	1.78
Lime1	Lck interacting transmembrane adaptor 1	1.77
Lims2	LIM and senescent cell antigen like domains 2	2.25

Lin28a	lin-28 homolog A (C. elegans)	2.09
Lin7b	lin-7 homolog b (C. elegans)	2.05
Lipa	lipase A, lysosomal acid, cholesterol esterase	1.86
Liph	lipase, member H	5.25
Lix1	Lix1 homolog (chicken)	3.32
Lmo1	LIM domain only 1	2.01
Lmo4	LIM domain only 4	2.35
Lmo7	LIM domain 7	1.90
Lmo7	LIM domain 7	2.03
Lmod1	leiomodin 1 (smooth muscle)	1.84
Lonrf1	LON peptidase N-terminal domain and ring finger 1	2.03
Lpar2	lysophosphatidic acid receptor 2	2.67
Lpar3	lysophosphatidic acid receptor 3	3.31
Lpgat1	lysophosphatidylglycerol acyltransferase 1	1.95
Lppr3	lipid phosphate phosphatase-related protein type 3	6.03
Lrch1	leucine-rich repeats and calponin homology (CH) domain containing 1	1.81
Lrfn3	leucine rich repeat and fibronectin type III domain containing 3	1.99
Lrp3	low density lipoprotein receptor-related protein 3	2.89
Lrp4	low density lipoprotein receptor-related protein 4	3.56
Lrrc1	leucine rich repeat containing 1	2.11
Lrrc15	leucine rich repeat containing 15	7.10
Lrrc16b	leucine rich repeat containing 16B	2.82
Lrrc23	leucine rich repeat containing 23	3.53
Lrrc37a	leucine rich repeat containing 37A	1.74
Lrrc58	leucine rich repeat containing 58	1.90
Lrrc8b	leucine rich repeat containing 8 family, member B	2.65
Lrrk1	leucine-rich repeat kinase 1	2.36
Lrrn4cl	LRRN4 C-terminal like	2.77
Lrrn4cl	LRRN4 C-terminal like	2.03
Ltb	lymphotoxin beta (TNF superfamily, member 3)	1.98
Ly6d	lymphocyte antigen 6 complex, locus D	2.61
Ly6g5b	lymphocyte antigen 6 complex, locus G5B	1.94
Ly6i	lymphocyte antigen 6 complex, locus I	4.92
Ly96	lymphocyte antigen 96	1.95
Ly96	lymphocyte antigen 96	2.10
Lyl1	lymphoblastic leukemia derived sequence 1	1.90
Lynx1	Ly6/neurotoxin 1	4.06
Lypd3	Ly6/Plaur domain containing 3	3.12
Lyrm9	LYR motif containing 9	1.84
Lyz2	lysozyme 2	3.38
Lyzl4	lysozyme-like 4	2.54
Lzts1	leucine zipper, putative tumor suppressor 1	1.91
Macrod1	MACRO domain containing 1	2.78
Mafa	v-maf musculoaponeurotic fibrosarcoma oncogene homolog A (avian)	2.19
Maff	v-maf musculoaponeurotic fibrosarcoma oncogene homolog F (avian)	2.62

Mafg	v-maf musculoaponeurotic fibrosarcoma oncogene homolog G (avian)	1.86
Mafk	v-maf musculoaponeurotic fibrosarcoma oncogene homolog K (avian)	2.05
Mageb18	melanoma antigen family B, 18	7.23
Mageb7	melanoma antigen, family B, 7	2.99
Magix	MAGI family member, X-linked	2.27
Mak	male germ cell-associated kinase	3.39
Maml1	mastermind like 1 (Drosophila)	2.79
Man2b2	mannosidase, alpha, class 2B, member 2	1.94
Manba	mannosidase, beta A, lysosomal	4.55
Map1b	microtubule-associated protein 1B	3.24
Map3k13	mitogen-activated protein kinase kinase kinase 13	2.68
Map3k2	mitogen activated protein kinase kinase kinase 2	2.42
Map7	microtubule-associated protein 7	2.82
Mapk1ip1	mitogen-activated protein kinase 1 interacting protein 1	1.75
Mapk8ip3	mitogen-activated protein kinase 8 interacting protein 3	2.54
Mapkbp1	mitogen activated protein kinase binding protein 1	2.06
Mapre3	microtubule-associated protein, RP/EB family, member 3	1.88
Mapt	microtubule-associated protein tau	2.77
Mapt	microtubule-associated protein tau	2.36
Mars2	methionyl-tRNA synthetase 2, mitochondrial	2.05
Mas1	MAS1 oncogene	2.16
MAST1	microtubule associated serine/threonine kinase 1	4.65
Mat2b	methionine adenosyltransferase II, beta	1.85
Mb21d2	Mab-21 domain containing 2	4.33
Mbnl1	muscleblind-like 1 (Drosophila)	2.09
Mbp	myelin basic protein	3.44
Mcpt8	mast cell protease 8	2.15
Mdfic	MyoD family inhibitor domain containing	1.90
Mdm2	p53 E3 ubiquitin protein ligase	2.94
Mdm2	p53 E3 ubiquitin protein ligase	3.11
Mdm4	Mdm4 p53 binding protein homolog (mouse)	2.25
Mecp2	methyl CpG binding protein 2	3.21
Med15	mediator complex subunit 15	2.70
Medag	mesenteric estrogen-dependent adipogenesis	2.88
Mef2b	myocyte enhancer factor 2B	3.52
Meig1	meiosis expressed gene 1	1.95
Meox1	mesenchyme homeobox 1	2.97
Mep1b	meprin 1 beta	5.48
Mepe	matrix extracellular phosphoglycoprotein	5.12
Mepe	matrix extracellular phosphoglycoprotein	4.16
Mertk	c-mer proto-oncogene tyrosine kinase	3.65
Mest	mesoderm specific transcript homolog (mouse)	2.55
Mettl8	methyltransferase like 8	2.14
Mfap5	microfibrillar associated protein 5	11.42
Mfng	MFNG O-fucosylpeptide 3-beta-N-acetylglucosaminyltransferase	2.98

Mfsd2a	major facilitator superfamily domain containing 2A	1.87
Mfsd6	major facilitator superfamily domain containing 6	2.87
Mfsd7	major facilitator superfamily domain containing 7	2.10
Mfsd7	major facilitator superfamily domain containing 7	2.49
Mgarp	mitochondria-localized glutamic acid-rich protein	2.22
Mgarp	mitochondria-localized glutamic acid-rich protein	1.88
Mgat5b	mannosyl (alpha-1,6-)-glycoprotein beta-1,6-N-acetyl-glucosaminyltransferase, isozyme B	5.55
Mgea5	meningioma expressed antigen 5 (hyaluronidase)	1.99
Mgmt	O-6-methylguanine-DNA methyltransferase	13.31
Mgst2	microsomal glutathione S-transferase 2	2.11
Mgst3	microsomal glutathione S-transferase 3	7.55
Mia	melanoma inhibitory activity	6.26
Micalcl	MICAL C-terminal like	2.40
Micb	MHC class I polypeptide-related sequence B	1.95
Mink1	misshapen-like kinase 1	1.76
Miox	myo-inositol oxygenase	2.91
Mlana	melan-A	3.83
Mmp17	matrix metallopeptidase 17	10.06
Mmp2	matrix metallopeptidase 2	4.03
Mmp28	matrix metallopeptidase 28	2.05
Mmp7	matrix metallopeptidase 7	6.10
Mocos	molybdenum cofactor sulfurase	2.12
Mogat1	monoacylglycerol O-acyltransferase 1	2.48
Morn5	MORN repeat containing 5	1.77
Mpa2l	macrophage activation 2 like	3.05
Mpp3	membrane protein, palmitoylated 3 (MAGUK p55 subfamily member 3)	2.57
Mpp3	membrane protein, palmitoylated 3 (MAGUK p55 subfamily member 3)	2.85
Mpp4	membrane protein, palmitoylated 4 (MAGUK p55 subfamily member 4)	2.66
Mpped1	metallophosphoesterase domain containing 1	1.90
Mpzl2	myelin protein zero-like 2	5.18
Mr1	major histocompatibility complex, class I-related	2.56
Mrap2	melanocortin 2 receptor accessory protein 2	3.16
Mreg	melanoregulin	1.84
Mro	maestro	32.58
Mroh7	maestro heat-like repeat family member 7	6.68
Mrps27	mitochondrial ribosomal protein S27	1.90
Ms4a11	membrane-spanning 4-domains, subfamily A, member 11	4.52
Msantd1	Myb/SANT-like DNA-binding domain containing 1	3.19
Msantd3	Myb/SANT-like DNA-binding domain containing 3	2.63
Msh5	mutS homolog 5 (E. coli)	4.91
Msl2	male-specific lethal 2 homolog (Drosophila)	1.86
Mt1a	metallothionein 1a	3.92
Mt2A	metallothionein 2A	1.98
Mt3	metallothionein 3	14.42

Mterfd3	MTERF domain containing 3	1.94
Mthfd2	methylenetetrahydrofolate dehydrogenase (NADP+ dependent) 2, methenyltetrahydrofolate cyclohydrolase	2.22
Mthfd2	methylenetetrahydrofolate dehydrogenase (NADP+ dependent) 2, methenyltetrahydrofolate cyclohydrolase	2.04
Mtmr10	myotubularin related protein 10	3.28
Mtmr3	myotubularin related protein 3	1.82
Mtss1	metastasis suppressor 1	2.08
Mtss1l	metastasis suppressor 1-like	2.11
Mtus1	microtubule associated tumor suppressor 1	2.77
Mtus1	microtubule associated tumor suppressor 1	4.28
Mxi1	MAX interactor 1	1.99
Mybl1	myeloblastosis oncogene-like 1	3.01
Mycbpap	Mycbp associated protein	6.59
Myh13	myosin, heavy chain 13, skeletal muscle	3.02
Myh3	myosin, heavy chain 3, skeletal muscle, embryonic	2.71
Myh3	myosin, heavy chain 3, skeletal muscle, embryonic	2.22
Myh6	myosin, heavy chain 6, cardiac muscle, alpha	2.04
Myh7	myosin, heavy chain 7, cardiac muscle, beta	1.92
Myh8	myosin, heavy chain 8, skeletal muscle, perinatal	2.36
Mylip	myosin regulatory light chain interacting protein	2.65
Mylk	myosin light chain kinase	2.67
Mylk	myosin light chain kinase	3.28
Mylk2	myosin light chain kinase 2	2.41
Mylk3	myosin light chain kinase 3	2.43
Myo1e	myosin IE	1.87
Myo1g	myosin IG	3.26
Myo5a	myosin VA	2.30
Myo7a	myosin VIIA	2.14
Myoc	myocilin	2.23
Myoz1	myozenin 1	2.17
N4bp2l1	NEDD4 binding protein 2-like 1	2.50
Naaladl1	N-acetylated alpha-linked acidic dipeptidase-like 1	1.89
Naf1	nuclear assembly factor 1 homolog (S. cerevisiae)	2.10
Naglu	N-acetylglucosaminidase, alpha	1.95
Naprt1	nicotinate phosphoribosyltransferase domain containing 1	2.21
Napsa	napsin A aspartic peptidase	4.84
Nat8b	N-acetyltransferase 8B	1.97
Nat8l	N-acetyltransferase 8-like	6.43
Ncam1	neural cell adhesion molecule 1	1.89
Ncf1	neutrophil cytosolic factor 1	1.91
Ncf4	neutrophil cytosolic factor 4	3.33
Ndn	necdin, melanoma antigen (MAGE) family member	3.12
Ndrg4	N-myc downstream regulated gene 4	1.88
Necab3	N-terminal EF-hand calcium binding protein 3	2.96

Nek5	NIMA-related kinase 5	1.94
Neurl	neuralized homolog (Drosophila)	1.83
Nexn	nexilin (F actin binding protein)	4.46
Nf1	neurofibromin 1	2.06
Nfat5	nuclear factor of activated T-cells 5	2.60
Nfatc2	nuclear factor of activated T-cells, cytoplasmic, calcineurin-dependent 2	2.52
Nfe2	nuclear factor, erythroid derived 2	3.62
Nfkbid	nuclear factor of kappa light polypeptide gene enhancer in B-cells inhibitor, delta	1.89
Nfxl1	nuclear transcription factor, X-box binding-like 1	1.75
Ngfr	nerve growth factor receptor	12.66
Nhej1	nonhomologous end-joining factor 1	2.10
Nhlrc1	NHL repeat containing 1	1.99
Nkain1	Na+/K+ transporting ATPase interacting 1	2.10
Nkd2	naked cuticle homolog 2 (Drosophila)	3.55
Nkd2	naked cuticle homolog 2 (Drosophila)	3.68
Nkpd1	NTPase, KAP family P-loop domain containing 1	2.44
Nkx2-5	NK2 homeobox 5	2.33
Nlrp4	NLR family, pyrin domain containing 4	1.77
Nlrp5	NLR family, pyrin domain containing 5	4.44
Nnmt	nicotinamide N-methyltransferase	1.84
Nol4	nucleolar protein 4	1.76
Nos2	nitric oxide synthase 2, inducible	2.97
Npas1	neuronal PAS domain protein 1	1.98
Npc1	Niemann-Pick disease, type C1	2.03
Nphs1	nephrosis 1, congenital, Finnish type (nephrin)	2.52
Npm2	nucleophosmin/nucleoplasmin 2	2.21
Nptxr	neuronal pentraxin receptor	2.78
Nptxr	neuronal pentraxin receptor	3.72
Npw	neuropeptide W	2.65
Npy	neuropeptide Y	7.90
Npy	neuropeptide Y	7.70
Nqo1	NAD(P)H dehydrogenase, quinone 1	6.22
Nr1d1	nuclear receptor subfamily 1, group D, member 1	6.22
Nr1d2	nuclear receptor subfamily 1, group D, member 2	2.66
Nr1h3	nuclear receptor subfamily 1, group H, member 3	2.17
Nr1h4	nuclear receptor subfamily 1, group H, member 4	4.39
Nr2f1	nuclear receptor subfamily 2, group F, member 1	1.75
Nr3c2	nuclear receptor subfamily 3, group C, member 2	5.79
Nr4a3	nuclear receptor subfamily 4, group A, member 3	3.96
Nr6a1	nuclear receptor subfamily 6, group A, member 1	2.97
Nrap	nebulin-related anchoring protein	3.16
Nrarp	Notch-regulated ankyrin repeat protein	3.40
Nrg1	neuregulin 1	4.92
Nrgn	neurogranin	2.99
Nrip1	nuclear receptor interacting protein 1	5.11

Nrip3	nuclear receptor interacting protein 3	1.91
Nrn1	neuritin 1	2.44
Nrn1l	neuritin 1-like	1.93
Nrp2	neuropilin 2	2.04
Nrtn	neurturin	1.86
Nsd1	nuclear receptor binding SET domain protein 1	2.03
Nsg2	neuron specific gene family member 2	4.19
Nsmaf	neutral sphingomyelinase (N-SMase) activation associated factor	1.88
Nt5dc3	5'-nucleotidase domain containing 3	2.59
Ntrk1	neurotrophic tyrosine kinase, receptor, type 1	4.41
Ntsr1	neurotensin receptor 1	2.50
Nudt7	nudix (nucleoside diphosphate linked moiety X)-type motif 7	3.53
Nup210	nucleoporin 210	2.01
Nupr1	nuclear protein, transcriptional regulator, 1	2.47
Nxf7	nuclear RNA export factor 7	11.89
Nxnl2	nucleoredoxin-like 2	2.34
Nxnl2	nucleoredoxin-like 2	2.37
Nxph3	neurexophilin 3	3.29
Nxph4	neurexophilin 4	4.28
Oas1a	2'-5' oligoadenylate synthetase 1A	2.04
Oas1e	2'-5' oligoadenylate synthetase 1E	2.05
Oas1h	2 ' -5 ' oligoadenylate synthetase 1H	5.49
Oas1k	2 ' -5 ' oligoadenylate synthetase 1K	2.02
Oas2	2'-5' oligoadenylate synthetase 2	2.08
Oasl	2'-5'-oligoadenylate synthetase-like	5.69
Obox5	oocyte specific homeobox 5	3.14
Ocm	oncomodulin	4.66
Ocm	oncomodulin	3.96
Ocrl	oculocerebrorenal syndrome of Lowe	2.35
Odc1	ornithine decarboxylase 1	1.78
Odf3b	outer dense fiber of sperm tails 3B	1.78
Olfml2a	olfactomedin-like 2A	6.08
Olr1	oxidized low density lipoprotein (lectin-like) receptor 1	2.54
Olr1555	olfactory receptor 1555	1.84
Olr165	olfactory receptor 165	2.01
Olr237	olfactory receptor 237	1.98
Olr402	olfactory receptor 402	2.22
Olr403	olfactory receptor 403	2.15
Olr425	olfactory receptor 425	1.88
Olr472	olfactory receptor 472	1.90
Olr714	olfactory receptor 714	1.75
Olr752	olfactory receptor 752	1.84
Olr791	olfactory receptor 791	1.97
Olr922	olfactory receptor 922	1.92
Onecut1	one cut homeobox 1	5.80

Onecut2	one cut homeobox 2	2.68
Osbpl10	oxysterol binding protein-like 10	2.26
Osgin1	oxidative stress induced growth inhibitor 1	5.33
Osr2	odd-skipped related 2 (Drosophila)	2.96
Ostm1	osteopetrosis associated transmembrane protein 1	1.86
Otos	otospiralin	1.85
Otud1	OTU domain containing 1	2.82
Ovol2	ovo-like 2 (Drosophila)	3.33
Pacsin1	protein kinase C and casein kinase substrate in neurons 1	2.04
Padi2	peptidyl arginine deiminase, type II	5.06
Pak3	p21 protein (Cdc42/Rac)-activated kinase 3	2.90
Papolb	poly (A) polymerase beta (testis specific)	1.94
Pard3b	par-3 partitioning defective 3 homolog B (C. elegans)	3.09
Pard6g	par-6 partitioning defective 6 homolog gamma (C. elegans)	2.93
Parp11	poly (ADP-ribose) polymerase family, member 11	3.94
Paxbp1	PAX3 and PAX7 binding protein 1	1.76
Pcdh10	protocadherin 10	4.47
Pcdh19	protocadherin 19	4.32
Pcdha1	protocadherin alpha 1	2.42
Pcdha3	protocadherin alpha 3	2.04
Pcdhb15	protocadherin beta 15	2.71
Pcdhb19	protocadherin beta 19	3.39
Pcdhb22	protocadherin beta 22	2.67
Pcdhg@	protocadherin gamma cluster	2.48
Pcdhga1	protocadherin gamma subfamily A, 1	1.98
Pcdhga11	protocadherin gamma subfamily A, 11	2.49
Pcdhga3	protocadherin gamma subfamily A, 3	2.46
Pcdhgb2	protocadherin gamma subfamily B, 2	2.08
Pcdhgb5	protocadherin gamma subfamily B, 5	2.72
Pced1b	PC-esterase domain containing 1B	3.44
Pcnxl2	pecanex-like 2 (Drosophila)	1.95
Pdcd1	programmed cell death 1	1.84
Pde2a	phosphodiesterase 2A, cGMP-stimulated	6.98
Pde4c	phosphodiesterase 4C, cAMP-specific	2.24
Pde5a	phosphodiesterase 5A, cGMP-specific	2.41
Pde6b	phosphodiesterase 6B, cGMP-specific, rod, beta	2.81
Pdgfrb	platelet derived growth factor receptor, beta polypeptide	2.19
Pdgfrl	platelet-derived growth factor receptor-like	1.82
Pdia5	protein disulfide isomerase family A, member 5	2.04
Pdx1	pancreatic and duodenal homeobox 1	2.35
Pdzd4	PDZ domain containing 4	1.93
Pecam1	platelet/endothelial cell adhesion molecule 1	1.98
Perp	PERP, TP53 apoptosis effector	1.99
Pfkfb2	6-phosphofructo-2-kinase/fructose-2,6-biphosphatase 2	2.70
Pgbd5	piggyBac transposable element derived 5	2.17

Pgf	placental growth factor	1.95
Pgf	placental growth factor	2.25
Pgm2l1	phosphoglucomutase 2-like 1	2.27
Pgm2l1	phosphoglucomutase 2-like 1	1.81
Phactr1	phosphatase and actin regulator 1	2.34
Phc3	polyhomeotic homolog 3 (Drosophila)	1.84
Phf11	PHD finger protein 11	2.74
Phf17	PHD finger protein 17	1.97
Phgr1	proline/histidine/glycine-rich 1	3.27
Phlda2	pleckstrin homology-like domain, family A, member 2	2.50
Phlda2	pleckstrin homology-like domain, family A, member 2	2.42
Phlda3	pleckstrin homology-like domain, family A, member 3	2.11
Pianp	PILR alpha associated neural protein	3.63
Pik3r1	phosphoinositide-3-kinase, regulatory subunit 1 (alpha)	1.79
Pikfyve	phosphoinositide kinase, FYVE finger containing	2.12
Pinlyp	phospholipase A2 inhibitor and LY6/PLAUR domain containing	2.47
Pip4k2a	phosphatidylinositol-5-phosphate 4-kinase, type II, alpha	2.11
Pip5k1b	phosphatidylinositol-4-phosphate 5-kinase, type I, beta	1.97
Pitx1	paired-like homeodomain 1	1.75
Pitx3	paired-like homeodomain 3	3.56
Pkd1	polycystic kidney disease 1 homolog (human)	1.82
Pkd2l1	polycystic kidney disease 2-like 1	2.75
Pla2g10	phospholipase A2, group X	2.08
Pla2g12a	phospholipase A2, group XIIA	1.84
Pla2g15	phospholipase A2, group XV	1.95
Pla2g16	phospholipase A2, group XVI	2.43
Pla2g2a	phospholipase A2, group IIA (platelets, synovial fluid)	1.81
Pla2g2c	phospholipase A2, group IIC	3.23
Pla2g4d	phospholipase A2, group IVD (cytosolic)	3.86
Plac1	placenta-specific 1	2.49
Plac8	placenta-specific 8	3.58
Plagl1	pleiomorphic adenoma gene-like 1	4.10
Plagl1	pleiomorphic adenoma gene-like 1	2.72
Plat	plasminogen activator, tissue	2.33
Plbd1	phospholipase B domain containing 1	1.98
Plcd3	phospholipase C, delta 3	1.84
Plcd4	phospholipase C, delta 4	5.51
Plcd4	phospholipase C, delta 4	4.17
Plcxd2	phosphatidylinositol-specific phospholipase C, X domain containing 2	2.13
Plcxd2	phosphatidylinositol-specific phospholipase C, X domain containing 2	1.76
Plek2	pleckstrin 2	4.50
Plekhb1	pleckstrin homology domain containing, family B (evectins) member 1	2.29
Plekhg1	pleckstrin homology domain containing, family G (with RhoGef domain) member 1	4.77
Plekhg1	pleckstrin homology domain containing, family G (with RhoGef domain)	2.76

	member 1	
Plekhh1	pleckstrin homology domain containing, family H (with MyTH4 domain) member 1	4.10
Plekhh1	pleckstrin homology domain containing, family H (with MyTH4 domain) member 1	3.25
Plk5	polo-like kinase 5	3.83
Pllp	plasmolipin	11.65
Pln	phospholamban	3.72
Pm20d2	peptidase M20 domain containing 2	1.80
Pmel	premelanosome protein	5.20
Pmepa1	prostate transmembrane protein, androgen induced 1	1.78
Pnkd	paroxysmal nonkinesigenic dyskinesia	1.90
Pnmal2	paraneoplastic Ma antigen family-like 2	2.57
Pnpla1	patatin-like phospholipase domain containing 1	2.18
Pnpla7	patatin-like phospholipase domain containing 7	3.42
Podnl1	podocan-like 1	2.21
Podnl1	podocan-like 1	2.19
Podxl	podocalyxin-like	2.30
Pogz	pogo transposable element with ZNF domain	1.86
Pou2af1	POU class 2 associating factor 1	1.96
Pou2f1	POU class 2 homeobox 1	2.03
Ppara	peroxisome proliferator activated receptor alpha	1.85
Ppargc1b	peroxisome proliferator-activated receptor gamma, coactivator 1 beta	2.01
Ppip5k1	diphosphoinositol pentakisphosphate kinase 1	2.79
Ppip5k1	diphosphoinositol pentakisphosphate kinase 1	2.04
Ppm1j	protein phosphatase, Mg2+/Mn2+ dependent, 1J	1.93
Ppm1l	protein phosphatase, Mg2+/Mn2+ dependent, 1L	1.84
Ppm1n	protein phosphatase, Mg2+/Mn2+ dependent, 1N	2.14
Ppp1r14c	protein phosphatase 1, regulatory (inhibitor) subunit 14c	1.87
Ppp1r1a	protein phosphatase 1, regulatory (inhibitor) subunit 1A	3.56
Ppp1r1a	protein phosphatase 1, regulatory (inhibitor) subunit 1A	3.66
Ppp1r1b	protein phosphatase 1, regulatory (inhibitor) subunit 1B	4.73
Ppp1r1b	protein phosphatase 1, regulatory (inhibitor) subunit 1B	2.07
Ppp1r32	protein phosphatase 1, regulatory subunit 32	1.92
Ppp1r3c	protein phosphatase 1, regulatory subunit 3C	3.95
Ppp2r2c	protein phosphatase 2, regulatory subunit B, gamma	2.15
Ppp2r3a	protein phosphatase 2, regulatory subunit B", alpha	2.25
Pragmin	pragma of Rnd2	2.04
Pramef12	PRAME family member 12	1.87
Prap1	proline-rich acidic protein 1	1.86
Prdm1	PR domain containing 1, with ZNF domain	4.04
Prelid2	PRELI domain containing 2	2.16
Prima1	proline rich membrane anchor 1	3.68
Prkaa1	protein kinase, AMP-activated, alpha 1 catalytic subunit	1.89
Prkaa2	protein kinase, AMP-activated, alpha 2 catalytic subunit	2.17

Prkab2	protein kinase, AMP-activated, beta 2 non-catalytic subunit	3.48
Prkag2	protein kinase, AMP-activated, gamma 2 non-catalytic subunit	10.25
Prkar1b	protein kinase, cAMP dependent regulatory, type I, beta	2.53
Prkch	protein kinase C, eta	1.79
Prl8a9	prolactin family 8, subfamily a, member 9	3.65
Prnd	prion protein 2 (dublet)	2.72
Prodh	proline dehydrogenase (oxidase) 1	2.38
Prom2	prominin 2	3.38
Pros1	protein S (alpha)	1.97
Prph	peripherin	2.70
Prss22	protease, serine, 22	2.96
Prss30	protease, serine, 30	2.75
Prss39	protease, serine, 39	2.71
Prss53	protease, serine, 53	1.75
Prtg	protogenin	2.31
Psma8	proteasome (prosome, macropain) subunit, alpha type, 8	2.43
Psors1c2	psoriasis susceptibility 1 candidate 2 (human)	1.83
Pspc1	paraspeckle component 1	1.81
Ptgr1	prostaglandin reductase 1	4.17
Pthlh	parathyroid hormone-like hormone	5.51
Ptk2b	PTK2B protein tyrosine kinase 2 beta	1.87
Ptpdc1	protein tyrosine phosphatase domain containing 1	1.97
Ptpn18	protein tyrosine phosphatase, non-receptor type 18	2.04
Ptpn3	protein tyrosine phosphatase, non-receptor type 3	1.80
Ptpn3	protein tyrosine phosphatase, non-receptor type 3	1.75
Ptpn5	protein tyrosine phosphatase, non-receptor type 5	5.06
Ptprh	protein tyrosine phosphatase, receptor type, H	3.07
Ptprn	protein tyrosine phosphatase, receptor type, N	3.26
Ptprv	protein tyrosine phosphatase, receptor type, V	2.01
Pus10	pseudouridylate synthase 10	3.81
Pvalb	parvalbumin	11.10
Pvrl4	poliovirus receptor-related 4	1.99
Pycr1	pyrroline-5-carboxylate reductase 1	1.95
Pygo1	pygopus 1	2.20
Qrich2	glutamine rich 2	2.05
Qsox1	quiescin Q6 sulfhydryl oxidase 1	1.83
R3hcc1l	R3H domain and coiled-coil containing 1-like	2.58
R3hdm2	R3H domain containing 2	1.98
Rab11fip1	RAB11 family interacting protein 1 (class I)	2.85
Rab20	RAB20, member RAS oncogene family	2.09
Rab30	RAB30, member RAS oncogene family	2.15
Rab3a	RAB3A, member RAS oncogene family	3.10
Rab3il1	RAB3A interacting protein (rabin3)-like 1	3.11
Rab6b	RAB6B, member RAS oncogene family	1.98
Rab7b	Rab7b, member RAS oncogene family	2.02

Rab9b	RAB9B, member RAS oncogene family	2.53
Rabggtb	Rab geranylgeranyltransferase, beta subunit	2.37
Rad51d	RAD51 homolog D (S. cerevisiae)	1.94
Rad52	RAD52 homolog (S. cerevisiae)	2.29
Radil	Ras association and DIL domains	2.80
Raet1e	retinoic acid early transcript 1E	2.65
Raet1l	retinoic acid early transcript 1L	3.18
Ralgds	ral guanine nucleotide dissociation stimulator	2.37
Ramp3	receptor (G protein-coupled) activity modifying protein 3	2.87
Ranbp3l	RAN binding protein 3-like	3.24
Rap1gap	Rap1 GTPase-activating protein	2.19
Rap1gap	Rap1 GTPase-activating protein	1.91
Rap1gap2	RAP1 GTPase activating protein 2	2.53
Rasa3	RAS p21 protein activator 3	1.81
Rasa4	RAS p21 protein activator 4	2.16
Rasa4	RAS p21 protein activator 4	2.20
Rasal1	RAS protein activator like 1 (GAP1 like)	2.42
Rasal3	RAS protein activator like 3	2.93
Rasgrp2	RAS guanyl releasing protein 2 (calcium and DAG-regulated)	2.15
Rasl10a	RAS-like, family 10, member A	2.13
Rassf5	Ras association (RalGDS/AF-6) domain family member 5	4.39
Rassf9	Ras association (RalGDS/AF-6) domain family (N-terminal) member 9	2.38
Rbm20	RNA binding motif protein 20	2.86
Rbm20	RNA binding motif protein 20	2.26
Rbm38	RNA binding motif protein 38	1.95
Rbpjl	recombination signal binding protein for immunoglobulin kappa J region-like	5.92
Rcbtb1	regulator of chromosome condensation (RCC1) and BTB (POZ) domain containing protein 1	2.76
Rcbtb1	regulator of chromosome condensation (RCC1) and BTB (POZ) domain containing protein 1	2.38
Rcor2	REST corepressor 2	1.81
Reep1	receptor accessory protein 1	1.92
Rem2	RAS (RAD and GEM) like GTP binding 2	2.37
Renbp	renin binding protein	1.75
Reps1	RALBP1 associated Eps domain containing 1	2.09
Rerg	RAS-like, estrogen-regulated, growth-inhibitor	1.77
Rftn1	raftlin lipid raft linker 1	2.16
Rftn2	raftlin family member 2	4.21
Rgl3	ral guanine nucleotide dissociation stimulator-like 3	1.82
Rgma	RGM domain family, member A	9.84
Rgs17	regulator of G-protein signaling 17	2.21
Rgs9	regulator of G-protein signaling 9	5.99
Rhebl1	Ras homolog enriched in brain like 1	2.06
Rhov	ras homolog family member V	3.85
Rhox10	reproductive homeobox 10	1.89

Rhox2	reproductive homeobox on X chromosome 2	1.96
Rhox4g	reproductive homeobox 4G	6.35
Rhox9	reproductive homeobox 9	2.08
Rhpn2	rhophilin, Rho GTPase binding protein 2	2.84
Rhpn2	rhophilin, Rho GTPase binding protein 2	2.17
Ribc2	RIB43A domain with coiled-coils 2	3.11
Rictor	RPTOR independent companion of MTOR, complex 2	2.01
Riiad1	regulatory subunit of type II PKA R-subunit (RIIa) domain containing 1	2.38
Rimkla	ribosomal modification protein rimK-like family member A	3.20
Rnase1	ribonuclease, RNase A family, 1 (pancreatic)	4.93
Rnase4	ribonuclease, RNase A family 4	4.77
Rnf112	ring finger protein 112	2.23
Rnf128	ring finger protein 128, E3 ubiquitin protein ligase	4.06
Rnf14	ring finger protein 14	1.83
Rnf144b	ring finger protein 144B	3.35
Rnf208	ring finger protein 208	3.83
Rnf217	ring finger protein 217	2.28
Rnf32	ring finger protein 32	1.75
Robo1	roundabout homolog 1 (Drosophila)	2.75
Robo3	roundabout homolog 3 (Drosophila)	3.47
Ror2	receptor tyrosine kinase-like orphan receptor 2	4.60
Rpgr	retinitis pigmentosa GTPase regulator	2.71
Rph3al	rabphilin 3A-like (without C2 domains)	3.18
Rprd1a	regulation of nuclear pre-mRNA domain containing 1A	2.23
Rprd1b	regulation of nuclear pre-mRNA domain containing 1B	1.92
Rps4y2	ribosomal protein S4, Y-linked 2	3.20
RragB	Ras-related GTP binding B	2.78
Rragd	Ras-related GTP binding D	4.35
Rsad2	radical S-adenosyl methionine domain containing 2	14.76
Rsph10b	radial spoke head 10 homolog B (Chlamydomonas)	4.37
Rsph6a	radial spoke head 6 homolog A (Chlamydomonas)	2.88
RT1-Bb	RT1 class II, locus Bb	10.66
RT1-Da	RT1 class II, locus Da	2.90
RT1-Db1	RT1 class II, locus Db1	12.26
RT1-Db1	RT1 class II, locus Db1	5.48
RT1-DMa	RT1 class II, locus DMa	2.06
RT1-DMb	RT1 class II, locus DMb	2.06
Rtbdn	retbindin	1.97
Rtn4r	reticulon 4 receptor	3.52
Rxfp1	relaxin/insulin-like family peptide receptor 1	1.78
Rybp	RING1 and YY1 binding protein	2.13
Rybp	RING1 and YY1 binding protein	2.19
S100a1	S100 calcium binding protein A1	2.39
S100a5	S100 calcium binding protein A5	2.89
S100a7a	S100 calcium binding protein A7A	1.86

S100a8	S100 calcium binding protein A8	2.75
S100g	S100 calcium binding protein G	2.85
S100pbp	S100P binding protein	2.11
S1pr1	sphingosine-1-phosphate receptor 1	6.49
Sall2	sal-like 2 (Drosophila)	2.46
Samd9l	sterile alpha motif domain containing 9-like	2.95
Samd9l	sterile alpha motif domain containing 9-like	4.08
Sarm1	sterile alpha and TIR motif containing 1	1.84
Scaper	S-phase cyclin A-associated protein in the ER	2.12
Scgb1c1	secretoglobin, family 1C, member 1	3.87
Scn11a	sodium channel, voltage-gated, type XI, alpha subunit	2.51
Scn1b	sodium channel, voltage-gated, type I, beta	2.35
Scn4b	sodium channel, voltage-gated, type IV, beta	2.71
Scn5a	sodium channel, voltage-gated, type V, alpha subunit	5.67
Scn8a	sodium channel, voltage gated, type VIII, alpha subunit	2.16
Scpep1	serine carboxypeptidase 1	1.91
Scyl3	SCY1-like 3 (S. cerevisiae)	1.78
Sdc2	syndecan 2	1.98
Sdc3	syndecan 3	4.40
Sdccag8	serologically defined colon cancer antigen 8	1.83
Sds	serine dehydratase	4.48
Sec16a	SEC16 homolog A (S. cerevisiae)	4.12
Sectm1b	secreted and transmembrane 1B	2.10
Selenbp1	selenium binding protein 1	2.02
Selp	selectin P	7.08
Sema3b	sema domain, immunoglobulin domain (Ig), short basic domain, secreted, (semaphorin) 3B	1.76
Sema6b	sema domain, transmembrane domain (TM), and cytoplasmic domain, (semaphorin) 6B	4.60
Serf1	small EDRK-rich factor 1	2.14
Serf1	small EDRK-rich factor 1	2.44
Serinc2	serine incorporator 2	1.81
Serpinb1a	serine (or cysteine) proteinase inhibitor, clade B, member 1a	5.38
Serpinb1a	serine (or cysteine) proteinase inhibitor, clade B, member 1a	5.28
Serpinb1b	serine (or cysteine) peptidase inhibitor, clade B, member 1b	1.92
Serpinb3a	serine (or cysteine) peptidase inhibitor, clade B (ovalbumin), member 3A	2.36
Serpine1	serpin peptidase inhibitor, clade E (nexin, plasminogen activator inhibitor type 1), member 1	4.00
Serpine2	serpin peptidase inhibitor, clade E (nexin, plasminogen activator inhibitor type 1), member 2	4.53
Serpinf1	serpin peptidase inhibitor, clade F (alpha-2 antiplasmin, pigment epithelium derived factor), member 1	1.75
Sesn2	sestrin 2	3.14
Sez6	seizure related 6 homolog (mouse)	3.07
Sez6l2	seizure related 6 homolog (mouse)-like 2	1.84
Sfrp2	secreted frizzled-related protein 2	1.81
Sftpc	surfactant protein C	1.94

Sftpd	surfactant protein D	2.13
Sfxn2	sideroflexin 2	2.00
Sgca	sarcoglycan, alpha (dystrophin-associated glycoprotein)	4.01
Sgce	sarcoglycan, epsilon	1.80
Sgk2	serum/glucocorticoid regulated kinase 2	2.88
Sgms2	sphingomyelin synthase 2	3.19
Sgpl1	sphingosine-1-phosphate lyase 1	1.86
Sh2b1	SH2B adaptor protein 1	1.96
Sh2d3c	SH2 domain containing 3C	2.30
Sh3bgrl2	SH3 domain binding glutamic acid-rich protein like 2	2.91
Sh3glb2	SH3-domain GRB2-like endophilin B2	1.80
Sh3kbp1	SH3-domain kinase binding protein 1	1.87
Sh3tc1	SH3 domain and tetratricopeptide repeats 1	1.81
Sh3yl1	SH3 domain containing, Ysc84-like 1 (S. cerevisiae)	1.95
Shc2	SHC (Src homology 2 domain containing) transforming protein 2	3.17
Shcbp1l	SHC SH2-domain binding protein 1-like	2.17
Shisa2	shisa homolog 2 (Xenopus laevis)	3.23
Shisa7	shisa homolog 7 (Xenopus laevis)	2.95
Shmt1	serine hydroxymethyltransferase 1 (soluble)	1.91
Shox2	short stature homeobox 2	2.27
Siah2	seven in absentia 2	2.99
Sigirr	single immunoglobulin and toll-interleukin 1 receptor (TIR) domain	1.85
Siglec10	sialic acid binding Ig-like lectin 10	4.83
Sipa1l2	signal-induced proliferation-associated 1 like 2	3.61
Sirt2	sirtuin 2	1.89
Sirt4	sirtuin 4	2.37
Slc12a3	solute carrier family 12 (sodium/chloride transporters), member 3	2.50
Slc12a5	solute carrier family 12 (potassium-chloride transporter), member 5	2.23
Slc13a3	solute carrier family 13 (sodium-dependent dicarboxylate transporter), member 3	5.45
Slc13a5	solute carrier family 13 (sodium-dependent citrate transporter), member 5	2.78
Slc15a1	solute carrier family 15 (oligopeptide transporter), member 1	3.84
Slc16a11	solute carrier family 16, member 11 (monocarboxylic acid transporter 11)	2.07
Slc16a14	solute carrier family 16, member 14 (monocarboxylic acid transporter 14)	1.93
Slc16a6	solute carrier family 16, member 6 (monocarboxylic acid transporter 7)	4.39
Slc17a5	solute carrier family 17 (anion/sugar transporter), member 5	2.94
Slc17a6	solute carrier family 17 (sodium-dependent inorganic phosphate cotransporter), member 6	3.94
Slc1a1	solute carrier family 1 (neuronal/epithelial high affinity glutamate transporter, system Xag), member 1	2.68
Slc22a3	solute carrier family 22 (extraneuronal monoamine transporter), member 3	2.22
Slc25a19	solute carrier family 25 (mitochondrial thiamine pyrophosphate carrier), member 19	1.85
Slc25a30	solute carrier family 25, member 30	3.92
Slc25a33	solute carrier family 25 (pyrimidine nucleotide carrier), member 33	1.79
Slc25a37	solute carrier family 25 (mitochondrial iron transporter), member 37	3.18
Slc25a40	solute carrier family 25, member 40	2.74

Slc25a47	solute carrier family 25, member 47	2.16
Slc27a5	solute carrier family 27 (fatty acid transporter), member 5	4.70
Slc2a4	solute carrier family 2 (facilitated glucose transporter), member 4	2.00
Slc2a5	solute carrier family 2 (facilitated glucose/fructose transporter), member 5	3.17
Slc30a1	solute carrier family 30 (zinc transporter), member 1	1.79
Slc30a3	solute carrier family 30 (zinc transporter), member 3	1.92
Slc35f2	solute carrier family 35, member F2	2.07
Slc35g1	solute carrier family 35, member G1	2.52
Slc36a2	solute carrier family 36 (proton/amino acid symporter), member 2	3.57
Slc38a5	solute carrier family 38, member 5	5.00
Slc38a7	solute carrier family 38, member 7	1.97
Slc3a2	solute carrier family 3 (activators of dibasic and neutral amino acid transport), member 2	2.22
Slc40a1	solute carrier family 40 (iron-regulated transporter), member 1	2.04
Slc40a1	solute carrier family 40 (iron-regulated transporter), member 1	2.76
Slc44a2	solute carrier family 44, member 2	2.80
Slc48a1	solute carrier family 48 (heme transporter), member 1	1.98
Slc4a11	solute carrier family 4, sodium borate transporter, member 11	1.87
Slc52a3	solute carrier family 52, riboflavin transporter, member 3	3.26
Slc6a2	solute carrier family 6 (neurotransmitter transporter, noradrenalin), member 2	1.96
Slc6a8	solute carrier family 6 (neurotransmitter transporter, creatine), member 8	1.80
Slc6a9	solute carrier family 6 (neurotransmitter transporter, glycine), member 9	1.77
Slc7a1	solute carrier family 7 (cationic amino acid transporter, y+ system), member 1	2.21
Slc7a11	solute carrier family 7 (anionic amino acid transporter light chain, xc- system), member 11	14.57
Slc7a3	solute carrier family 7 (cationic amino acid transporter, y+ system), member 3	5.69
Slc7a8	solute carrier family 7 (amino acid transporter light chain, L system), member 8	3.85
Slc9a2	solute carrier family 9, subfamily A (NHE2, cation proton antiporter 2), member 2	2.12
Slc9a7	solute carrier family 9 (sodium/hydrogen exchanger), member 7	1.99
Slc9b2	solute carrier family 9, subfamily B (NHA2, cation proton antiporter 2), member 2	2.00
Slco4a1	solute carrier organic anion transporter family, member 4a1	2.33
Slfn3	schlafen 3	3.10
Slit1	slit homolog 1 (Drosophila)	3.28
Slit3	slit homolog 3 (Drosophila)	2.21
Slmo1	slowmo homolog 1 (Drosophila)	3.08
Slmo1	slowmo homolog 1 (Drosophila)	2.07
Smarca1	SWI/SNF related, matrix associated, actin dependent regulator of chromatin, subfamily a, member 1	2.06
Smarca2	SWI/SNF related, matrix associated, actin dependent regulator of chromatin, subfamily a, member 2	2.41
Smg5	Smg-5 homolog, nonsense mediated mRNA decay factor (C. elegans)	1.77
Smim5	small integral membrane protein 5	2.01
Smpd3	sphingomyelin phosphodiesterase 3, neutral membrane	3.21
Smtnl2	smoothelin-like 2	2.87
Snai2	snail homolog 2 (Drosophila)	2.30

Snhg11	small nucleolar RNA host gene 11 (non-protein coding)	2.28
Snph	syntaphilin	3.91
Snrpn	small nuclear ribonucleoprotein polypeptide N	4.52
Snurf	SNRPN upstream reading frame	3.31
Snx20	sorting nexin 20	13.31
Snx30	sorting nexin family member 30	2.49
Socs1	suppressor of cytokine signaling 1	2.95
Sod3	superoxide dismutase 3, extracellular	1.78
Sorbs2	sorbin and SH3 domain containing 2	3.17
Sorbs2	sorbin and SH3 domain containing 2	2.22
Sorcs2	sortilin-related VPS10 domain containing receptor 2	4.88
Sox11	SRY (sex determining region Y)-box 11	1.87
Sox13	SRY (sex determining region Y)-box 13	2.18
Sox18	SRY (sex determining region Y)-box 18	1.97
Sox8	SRY (sex determining region Y)-box 8	12.80
Sp100	SP100 nuclear antigen	1.79
Spag1	sperm associated antigen 1	1.96
Spata19	spermatogenesis associated 19	2.54
Spata31d1d	spermatogenesis associated 31 subfamily D, member 1D	2.66
Spats1	spermatogenesis associated, serine-rich 1	7.21
Spdef	SAM pointed domain containing ets transcription factor	1.79
Spdya	speedy homolog A (Xenopus laevis)	2.57
Speg	SPEG complex locus	2.07
Spetex-2A	Spetex-2A protein	8.76
Spetex-2F	Spetex-2F protein	3.42
Spetex-2G	Spetex-2G protein	1.77
Sphk1	sphingosine kinase 1	1.91
Spib	Spi-B transcription factor (Spi-1/PU.1 related)	3.19
Spink4	serine peptidase inhibitor, Kazal type 4	2.67
Spink8	serine peptidase inhibitor, Kazal type 8	8.22
Spint1	serine peptidase inhibitor, Kunitz type 1	1.88
Spns2	spinster homolog 2	4.42
Spocd1-ps1	SPOC domain containing 1, pseudogene 1	3.23
Spp1	secreted phosphoprotein 1	2.60
Spry3	sprouty homolog 3 (Drosophila)	2.07
Sptb	spectrin, beta, erythrocytic	2.54
Sqrdl	sulfide quinone reductase-like (yeast)	2.27
Sqstm1	sequestosome 1	1.98
Srgn	serglycin	4.28
Srpx2	sushi-repeat-containing protein, X-linked 2	4.17
Srxn1	sulfiredoxin 1	3.14
Ss18l1	synovial sarcoma translocation gene on chromosome 18-like 1	1.85
Ssc5d	scavenger receptor cysteine rich domain containing (5 domains)	1.88
Sspo	SCO-spondin	5.32
Sstr3	somatostatin receptor 3	2.98

Stag3	stromal antigen 3	2.30
Stard13	StAR-related lipid transfer (START) domain containing 13	2.17
Stard13	StAR-related lipid transfer (START) domain containing 13	3.20
Stbd1	starch binding domain 1	2.55
Stc2	stanniocalcin 2	6.11
Steap3	STEAP family member 3, metalloreductase	2.04
Stim1	stromal interaction molecule 1	2.06
Stox1	storkhead box 1	2.25
Stx16	syntaxin 16	2.73
Stx1a	syntaxin 1A (brain)	1.75
Stx3	syntaxin 3	1.85
Stxbp1	syntaxin binding protein 1	2.28
Stxbp5	syntaxin binding protein 5 (tomosyn)	2.84
Stxbp6	syntaxin binding protein 6 (amisyn)	2.23
Sulf2	sulfatase 2	3.30
Sult5a1	sulfotransferase family 5A, member 1	2.67
Sun2	Sad1 and UNC84 domain containing 2	2.22
Sun2	Sad1 and UNC84 domain containing 2	2.19
Susd4	sushi domain containing 4	2.47
Susd4	sushi domain containing 4	2.75
Svip	small VCP/p97-interacting protein	3.28
Syce3	synaptonemal complex central element protein 3	2.91
Syn1	synapsin I	2.55
Syn2	synapsin II	2.37
Synm	synemin, intermediate filament protein	2.83
Synm	synemin, intermediate filament protein	2.13
Synpo2l	synaptopodin 2-like	3.52
Syt11	synaptotagmin XI	1.77
Syt17	synaptotagmin XVII	2.00
Syt3	synaptotagmin III	2.01
Syt5	synaptotagmin V	2.35
Syt7	synaptotagmin VII	3.05
T2	brachyury 2	3.48
Tab1	TGF-beta activated kinase 1/MAP3K7 binding protein 1	1.91
Tac2	tachykinin 2	6.10
Tacr2	tachykinin receptor 2	6.36
Taf7l	TAF7-like RNA polymerase II, TATA box binding protein (TBP)-associated factor	22.07
Tanc2	tetratricopeptide repeat, ankyrin repeat and coiled-coil containing 2	4.39
Tanc2	tetratricopeptide repeat, ankyrin repeat and coiled-coil containing 2	2.81
Tarsl2	threonyl-tRNA synthetase-like 2	1.77
Tbc1d2b	TBC1 domain family, member 2B	1.83
Tbc1d5	TBC1 domain family, member 5	1.89
Tbc1d9	TBC1 domain family, member 9 (with GRAM domain)	1.89
Tbc1d9	TBC1 domain family, member 9 (with GRAM domain)	1.91

Tbkbp1	TBK1 binding protein 1	2.43
Tbx3	T-box 3	2.75
Tbxas1	thromboxane A synthase 1, platelet	3.43
Tcap	titin-cap	2.03
Tceal7	transcription elongation factor A (SII)-like 7	2.20
Tcf24	transcription factor 24	3.22
Tcp11	t-complex protein 11	10.11
Tcte1	t-complex-associated testis expressed 1	2.42
Tekt2	tektin 2 (testicular)	1.92
Tekt4	tektin 4	4.17
Tex264	testis expressed 264	1.78
Tfap4	transcription factor AP-4	2.16
Tfcp2l1	transcription factor CP2-like 1	1.80
Tff2	trefoil factor 2	8.21
Tff3	trefoil factor 3, intestinal	8.77
Tg	thyroglobulin	2.64
Tgfa	transforming growth factor alpha	2.27
Tgfbr2	transforming growth factor, beta receptor II	2.08
Theg	testicular haploid expressed gene	7.21
Themis2	thymocyte selection associated family member 2	2.37
Timm8a2	translocase of inner mitochondrial membrane 8A2	2.32
Tinagl1	tubulointerstitial nephritis antigen-like 1	1.99
Tiparp	TCDD-inducible poly(ADP-ribose) polymerase	1.90
Tlr4	toll-like receptor 4	3.86
Tmcc2	transmembrane and coiled-coil domain family 2	1.91
Tmem108	transmembrane protein 108	2.14
Tmem131	transmembrane protein 131	1.74
Tmem145	transmembrane protein 145	4.37
Tmem14a	transmembrane protein 14A	3.17
Tmem150a	transmembrane protein 150A	1.76
Tmem170a	transmembrane protein 170A	1.91
Tmem184a	transmembrane protein 184A	1.74
Tmem2	transmembrane protein 2	2.07
Tmem220	transmembrane protein 220	2.89
Tmem229b	transmembrane protein 229B	2.78
Tmem229b	transmembrane protein 229B	1.87
Tmem231	transmembrane protein 231	2.14
Tmem25	transmembrane protein 25	1.86
Tmem38b	transmembrane protein 38B	3.27
Tmem59l	transmembrane protein 59-like	3.63
Tmem63c	transmembrane protein 63c	2.10
Tmem74	transmembrane protein 74	10.31
Tmem86a	transmembrane protein 86A	3.09
Tmem86a	transmembrane protein 86A	2.56
Tmem8a	transmembrane protein 8A	2.38

Tmem8b	transmembrane protein 8B	2.18
Tmod1	tropomodulin 1	2.11
Tmod4	tropomodulin 4	2.03
Tmx4	thioredoxin-related transmembrane protein 4	2.19
Tnfrsf17	tumor necrosis factor receptor superfamily, member 17	2.29
Tnfrsf21	tumor necrosis factor receptor superfamily, member 21	1.78
Tnfrsf9	tumor necrosis factor receptor superfamily, member 9	3.17
Tnfsf9	tumor necrosis factor (ligand) superfamily, member 9	1.95
Tnik	TRAF2 and NCK interacting kinase	3.62
Tnk2	tyrosine kinase, non-receptor, 2	2.34
Tnk2	tyrosine kinase, non-receptor, 2	1.92
Tnk2	tyrosine kinase, non-receptor, 2	2.05
Tnnt1	troponin T type 1 (skeletal, slow)	4.16
Tnnt1	troponin T type 1 (skeletal, slow)	4.64
Tns4	tensin 4	1.94
Tollip	toll interacting protein	1.81
Tor1aip2	torsin A interacting protein 2	1.77
Tp53i11	tumor protein p53 inducible protein 11	1.80
Tp53rk	TP53 regulating kinase	1.82
Tpd52l1	tumor protein D52-like 1	2.76
Tph1	tryptophan hydroxylase 1	1.95
Tpp1	tripeptidyl peptidase I	2.20
Traf3ip3	TRAF3 interacting protein 3	2.26
Traf6	TNF receptor-associated factor 6, E3 ubiquitin protein ligase	2.10
Trap1a	tumor rejection antigen P1A	3.25
Trem3	triggering receptor expressed on myeloid cells 3	5.37
Trex2	three prime repair exonuclease 2	2.14
Trhr2	thyrotropin releasing hormone receptor 2	7.55
Trib3	tribbles homolog 3 (Drosophila)	4.80
Trim29	tripartite motif-containing 29	7.53
Trim9	tripartite motif-containing 9	2.14
Triqk	triple QxxK/R motif containing	4.74
Trnp1	TMF1-regulated nuclear protein 1	3.15
Trove2	TROVE domain family, member 2	2.16
Trpv2	transient receptor potential cation channel, subfamily V, member 2	3.62
Tsga13	testis specific, 13	2.72
Tshz1	teashirt zinc finger homeobox 1	2.89
Tsks	testis-specific serine kinase substrate	2.19
Tsnaxip1	translin-associated factor X interacting protein 1	1.95
Tspan13	tetraspanin 13	1.75
Tspan18	tetraspanin 18	1.80
Tspan7	tetraspanin 7	2.67
Tspan8	tetraspanin 8	1.87
Tspan9	tetraspanin 9	1.78
Ttc13	tetratricopeptide repeat domain 13	1.96

Ttc3	tetratricopeptide repeat domain 3	1.99
Ttc7	tetratricopeptide repeat domain 7	1.76
Ttc7b	tetratricopeptide repeat domain 7B	1.92
Ttc8	tetratricopeptide repeat domain 8	3.80
Ttc9b	tetratricopeptide repeat domain 9B	2.30
Ttll13	tubulin tyrosine ligase-like family, member 13	1.83
Ttll3	tubulin tyrosine ligase-like family, member 3	2.85
Ttll7	tubulin tyrosine ligase-like family, member 7	1.91
Ttll7	tubulin tyrosine ligase-like family, member 7	2.41
Tubb2a	tubulin, beta 2A class IIa	1.79
Tubb3	tubulin, beta 3 class III	3.43
Tulp1	tubby like protein 1	2.15
Txndc16	thioredoxin domain containing 16	2.98
Txnrd1	thioredoxin reductase 1	1.86
Txnrd3	thioredoxin reductase 3	2.94
Uap1	UDP-N-acteylglucosamine pyrophosphorylase 1	1.93
Uap1l1	UDP-N-acteylglucosamine pyrophosphorylase 1-like 1	2.83
Ubald1	UBA-like domain containing 1	1.79
Ubap1	ubiquitin-associated protein 1	1.90
Ubd	ubiquitin D	5.71
Ube2d1	ubiquitin-conjugating enzyme E2D 1	2.19
Ubp1	upstream binding protein 1 (LBP-1a)	1.84
Uchl1	ubiquitin carboxyl-terminal esterase L1 (ubiquitin thiolesterase)	5.58
Ugt1a6	UDP glucuronosyltransferase 1 family, polypeptide A6	2.49
Ugt2b37	UDP-glucuronosyltransferase 2 family, member 37	2.47
Unc45b	unc-45 homolog B (C. elegans)	3.08
Unc5c	unc-5 homolog C (C. elegans)	1.94
Unc5cl	unc-5 homolog C (C. elegans)-like	7.62
Uncx	UNC homeobox	2.48
Upb1	ureidopropionase, beta	2.36
Upk2	uroplakin 2	6.57
Upp1	uridine phosphorylase 1	2.03
Ush1c	Usher syndrome 1C homolog (human)	2.03
Usp18	ubiquitin specific peptidase 18	6.92
Usp18	ubiquitin specific peptidase 18	5.75
Usp2	ubiquitin specific peptidase 2	1.79
Usp31	ubiquitin specific peptidase 31	1.82
Usp43	ubiquitin specific peptidase 43	3.18
Usp44	ubiquitin specific peptidase 44	2.09
Usp46	ubiquitin specific peptidase 46	2.43
Usp49	ubiquitin specific peptidase 49	1.79
Ust	uronyl-2-sulfotransferase	2.34
Utrn	utrophin	1.74
Utrn	utrophin	1.78
Vamp1	vesicle-associated membrane protein 1	2.03

Vamp4	vesicle-associated membrane protein 4	2.02
Vat1	vesicle amine transport protein 1 homolog (T californica)	2.02
Vegfa	vascular endothelial growth factor A	3.47
Vegfa	vascular endothelial growth factor A	2.71
Vegfa	vascular endothelial growth factor A	3.05
Vegfa	vascular endothelial growth factor A	2.36
Vegfc	vascular endothelial growth factor C	2.16
Vegp2	von Ebners gland protein 2	2.19
Vgf	VGF nerve growth factor inducible	3.98
Vill	villin-like	2.04
Vmo1	vitelline membrane outer layer 1 homolog (chicken)	2.93
Vof16	ischemia related factor vof-16	2.44
Vom1r30	vomeronasal 1 receptor 30	1.99
Vom2r46	vomeronasal 2 receptor, 46	2.20
Vps37d	vacuolar protein sorting 37 homolog D (S. cerevisiae)	2.12
Vstm4	V-set and transmembrane domain containing 4	3.69
Vtcn1	V-set domain containing T cell activation inhibitor 1	2.59
Vwa1	von Willebrand factor A domain containing 1	1.96
Vwa7	von Willebrand factor A domain containing 7	3.12
Wars2	tryptophanyl tRNA synthetase 2 (mitochondrial)	2.07
Wars2	tryptophanyl tRNA synthetase 2 (mitochondrial)	2.18
Wdr86	WD repeat domain 86	3.15
Wfdc1	WAP four-disulfide core domain 1	4.37
Wfdc10	WAP four-disulfide core domain 10	4.06
Wfdc10	WAP four-disulfide core domain 10	5.64
Wfdc11	WAP four-disulfide core domain 11	4.36
Wfdc12	WAP four-disulfide core domain 12	3.59
Wfdc15a	WAP four-disulfide core domain 15A	3.31
Wfdc18	WAP four-disulfide core domain 18	6.26
Wfdc18	WAP four-disulfide core domain 18	9.06
Wfdc3	WAP four-disulfide core domain 3	6.10
Wif1	Wnt inhibitory factor 1	4.34
Wisp2	WNT1 inducible signaling pathway protein 2	1.81
Wnk2	WNK lysine deficient protein kinase 2	2.14
Wnk4	WNK lysine deficient protein kinase 4	1.98
Wnt11	wingless-type MMTV integration site family, member 11	4.54
Wnt11	wingless-type MMTV integration site family, member 11	3.42
Wnt3	wingless-type MMTV integration site family, member 3	1.91
Wnt4	wingless-type MMTV integration site family, member 4	2.71
Wnt7a	wingless-type MMTV integration site family, member 7A	4.17
Wnt7b	wingless-type MMTV integration site family, member 7B	3.88
Wwox	WW domain-containing oxidoreductase	3.40
Xcl1	chemokine (C motif) ligand 1	1.85
Xdh	xanthine dehydrogenase	4.88
Xpo5	exportin 5	1.90

Xylb	xylulokinase homolog (H. influenzae)	3.22
Ybx2	Y box binding protein 2	2.21
Zadh2	zinc binding alcohol dehydrogenase, domain containing 2	3.01
Zadh2	zinc binding alcohol dehydrogenase, domain containing 2	3.24
Zar1l	zygote arrest 1-like	2.65
Zar1l	zygote arrest 1-like	3.45
Zbtb10	zinc finger and BTB domain containing 10	2.11
Zbtb26	zinc finger and BTB domain containing 26	2.07
Zbtb34	zinc finger and BTB domain containing 34	1.76
Zbtb38	zinc finger and BTB domain containing 38	3.11
Zbtb39	zinc finger and BTB domain containing 39	2.08
Zbtb43	zinc finger and BTB domain containing 43	2.31
Zbtb46	zinc finger and BTB domain containing 46	2.30
Zbtb7b	zinc finger and BTB domain containing 7B	2.30
Zbtb7c	zinc finger and BTB domain containing 7C	4.35
Zbtb8b	zinc finger and BTB domain containing 8b	3.70
Zc3h8	zinc finger CCCH type containing 8	2.28
Zcchc2	zinc finger, CCHC domain containing 2	2.11
Zcchc2	zinc finger, CCHC domain containing 2	2.57
Zdhhc2	zinc finger, DHHC-type containing 2	2.63
Zeb1	zinc finger E-box binding homeobox 1	1.90
Zeb2	zinc finger E-box binding homeobox 2	2.97
Zfp12	zinc finger protein 12	1.79
Zfp133	zinc finger protein 133	2.49
Zfp141	zinc finger protein 141	2.16
Zfp169	zinc finger protein 169	2.75
Zfp187	zinc finger protein 187	1.77
Zfp2	zinc finger protein 2	2.91
Zfp219	zinc finger protein 219	1.97
Zfp275	zinc finger protein 275	1.82
Zfp280c	zinc finger protein 280C	2.14
Zfp295	zinc finger protein 295	2.60
Zfp296	zinc finger protein 296	2.47
Zfp394	zinc finger protein 394	2.26
Zfp40	zinc finger protein 40	1.82
Zfp418	zinc finger protein 418	2.08
Zfp438	zinc finger protein 438	2.05
Zfp496	zinc finger protein 496	2.02
Zfp496	zinc finger protein 496	2.23
Zfp503	zinc finger protein 503	6.53
Zfp53	zinc finger protein 53	1.81
Zfp536	zinc finger protein 536	3.46
Zfp579	zinc finger protein 579	2.87
Zfp597	zinc finger protein 597	2.43
Zfp692	zinc finger protein 692	1.81

Zfp777	zinc finger protein 777	1.98
Zfp780b	zinc finger protein 780B	1.83
Zfp786	zinc finger protein 786	3.09
Zfp799	zinc finger protein 799	1.86
Zfp846	zinc finger protein 846	1.83
Zfp870	zinc finger protein 870	1.78
Zfp94	zinc finger protein 94	2.05
Zfp956	zinc finger protein 956	3.15
Zfr2	zinc finger RNA binding protein 2	2.47
Zfyve20	zinc finger, FYVE domain containing 20	2.13
Zfyve28	zinc finger, FYVE domain containing 28	2.39
Zhx2	zinc fingers and homeoboxes 2	2.29
Zmat3	zinc finger, matrin type 3	2.39
Zmynd10	zinc finger, MYND-type containing 10	1.88
Zp3	zona pellucida glycoprotein 3 (sperm receptor)	2.10
Zscan20	zinc finger and SCAN domain containing 20	2.42
Zwilch	zwilch kinetochore protein	1.76

Supplementary table 3: List of down-regulated genes upon 3-NPA treatment. The Gene symbol, Gene name and fold change in expression (3-NPA/Control) are indicated.

Gene Symbol	Gene Name	Fold change
A3galt2	alpha 1,3-galactosyltransferase 2	0.35
Abhd1	abhydrolase domain containing 1	0.55
Abhd11	abhydrolase domain containing 11	0.53
Acaa1a	acetyl-Coenzyme A acyltransferase 1A	0.56
Acat1	acetyl-CoA acetyltransferase 1	0.50
Acat3	acetyl-Coenzyme A acetyltransferase 3	0.52
Acsl3	acyl-CoA synthetase long-chain family member 3	0.46
Acss2	acyl-CoA synthetase short-chain family member 2	0.53
Actl6a	actin-like 6A	0.50
Actr1a	ARP1 actin-related protein 1 homolog A, centractin alpha (yeast)	0.56
Acy1	aminoacylase 1	0.51
Adamts1	ADAM metallopeptidase with thrombospondin type 1 motif, 1	0.12
Adamts10	ADAM metallopeptidase with thrombospondin type 1 motif, 10	0.50
Adm	adrenomedullin	0.55
Adra1d	adrenoceptor alpha 1D	0.01
Agrn	agrin	0.56
Akr1a1	aldo-keto reductase family 1, member A1 (aldehyde reductase)	0.57
Aldh16a1	aldehyde dehydrogenase 16 family, member A1	0.56
Aldoa	aldolase A, fructose-bisphosphate	0.51
Aldoart1	aldolase 1 A retrogene 1	0.48
Aldoc	aldolase C, fructose-bisphosphate	0.49
Alg8	asparagine-linked glycosylation 8, alpha-1,3-glucosyltransferase homolog (S. cerevisiae)	0.57
Anapc10	anaphase promoting complex subunit 10	0.51
Anapc15	anaphase promoting complex subunit 15	0.55
Angptl4	angiopoietin-like 4	0.55
Ankrd1	ankyrin repeat domain 1	0.53
Anks4b	ankyrin repeat and sterile alpha motif domain containing 4B	0.35
Ankzf1	ankyrin repeat and zinc finger domain containing 1	0.51
Anxa1	annexin A1	0.56
Apex1	APEX nuclease (multifunctional DNA repair enzyme) 1	0.53
Apoa2	apolipoprotein A-II	0.39
Arf4	ADP-ribosylation factor 4	0.50
Arfrp1	ADP-ribosylation factor related protein 1	0.41
Arhgap28	Rho GTPase activating protein 28	0.50
Arhgef4	Rho guanine nucleotide exchange factor (GEF) 4	0.18
Asb5	ankyrin repeat and SOCS box-containing 5	0.06
Atg3	autophagy related 3	0.45
Atoh8	atonal homolog 8 (Drosophila)	0.48

Atp5g3	ATP synthase, H+ transporting, mitochondrial Fo complex, subunit C3 (subunit 9)	0.51
Atp5s	ATP synthase, H+ transporting, mitochondrial Fo complex, subunit s (factor B)	0.39
Atp6v1e2	ATPase, H transporting, lysosomal V1 subunit E2	0.44
Avil	advillin	0.16
Axl	Axl receptor tyrosine kinase	0.52
B9d1	B9 protein domain 1	0.49
Bcl2l11	BCL2-like 11 (apoptosis facilitator)	0.51
Bcl2l12	BCL2-like 12 (proline rich)	0.46
Bcl3	B-cell CLL/lymphoma 3	0.51
Bnip2	BCL2/adenovirus E1B interacting protein 2	0.46
Btf3	basic transcription factor 3	0.53
Btnl5	butyrophilin-like 5	0.45
Btnl7	butyrophilin-like 7	0.27
Bzw2	basic leucine zipper and W2 domains 2	0.56
C4bpa	complement component 4 binding protein, alpha	0.32
C5	complement component 5	0.15
Cadm1	cell adhesion molecule 1	0.56
Calr	calreticulin	0.55
Cand2	cullin-associated and neddylation-dissociated 2 (putative)	0.51
Caprin1	cell cycle associated protein 1	0.49
Carkd	carbohydrate kinase domain containing	0.56
Casp9	caspase 9, apoptosis-related cysteine peptidase	0.42
Cav2	caveolin 2	0.04
Cbs	cystathionine beta synthase	0.17
Ccdc80	coiled-coil domain containing 80	0.51
Ccl2	chemokine (C-C motif) ligand 2	0.52
Ccl20	chemokine (C-C motif) ligand 20	0.10
Ccl20	chemokine (C-C motif) ligand 20	0.09
Ccna2	cyclin A2	0.55
Ccna2	cyclin A2	0.52
Ccnb1	cyclin B1	0.52
Ccnd3	cyclin D3	0.50
Ccnl2	cyclin L2	0.30
Cd82	Cd82 molecule	0.51
Cdc20	cell division cycle 20 homolog (S. cerevisiae)	0.49
Cdc42	cell division cycle 42 (GTP binding protein)	0.47
Cdc42se2	CDC42 small effector 2	0.54
Cdh6	cadherin 6	0.56
Cdk2ap1	cyclin-dependent kinase 2 associated protein 1	0.55
Cdk5	cyclin-dependent kinase 5	0.56
Cdkl3	cyclin-dependent kinase-like 3	0.43
Cdkn2c	cyclin-dependent kinase inhibitor 2C (p18, inhibits CDK4)	0.31
Cenpa	centromere protein A	0.55
Cetn2	centrin, EF-hand protein, 2	0.36
Cetn3	centrin, EF-hand protein, 3	0.50

Cfb	complement factor B	0.50
Cfl2	cofilin 2, muscle	0.48
Cfl2	cofilin 2, muscle	0.45
Chid1	chitinase domain containing 1	0.39
Chtf18	CTF18, chromosome transmission fidelity factor 18 homolog (S. cerevisiae)	0.44
Cinp	cyclin-dependent kinase 2-interacting protein	0.52
Cish	cytokine inducible SH2-containing protein	0.45
Cited1	Cbp/p300-interacting transactivator with Glu/Asp-rich carboxy-terminal domain 1	0.24
Clec2dl1	C-type lectin domain family 2 member D-like 1	0.42
Cmtm6	CKLF-like MARVEL transmembrane domain containing 6	0.41
Cnih2	cornichon homolog 2 (Drosophila)	0.52
Col3a1	collagen, type III, alpha 1	0.29
Col4a5	collagen, type IV, alpha 5	0.48
Colec10	collectin sub-family member 10 (C-type lectin)	0.01
Colec12	collectin sub-family member 12	0.54
Commd5	COMM domain containing 5	0.56
Cope	coatomer protein complex, subunit epsilon	0.52
Copz1	coatomer protein complex, subunit zeta 1	0.57
Coq4	coenzyme Q4 homolog (S. cerevisiae)	0.46
Coq7	coenzyme Q7 homolog, ubiquinone (yeast)	0.56
Cp	ceruloplasmin (ferroxidase)	0.43
Cpsf6	cleavage and polyadenylation specific factor 6, 68kDa	0.55
Csmd3	CUB and Sushi multiple domains 3	0.28
Ctnnal1	catenin (cadherin associated protein), alpha-like 1	0.34
Cwf19l2	CWF19-like 2, cell cycle control (S. pombe)	0.57
Cxcl12	chemokine (C-X-C motif) ligand 12	0.26
Cxcl12	chemokine (C-X-C motif) ligand 12	0.35
Cxcl2	chemokine (C-X-C motif) ligand 2	0.53
Cxcl3	chemokine (C-X-C motif) ligand 3	0.53
Cxcr7	chemokine (C-X-C motif) receptor 7	0.24
Cyb561d2	cytochrome b-561 domain containing 2	0.50
D2hgdh	D-2-hydroxyglutarate dehydrogenase	0.48
Dad1	Defender against cell death 1	0.56
Dapk3	death-associated protein kinase 3	0.54
Dck	deoxycytidine kinase	0.45
Ddx49	DEAD (Asp-Glu-Ala-Asp) box polypeptide 49	0.43
Ddx56	DEAD (Asp-Glu-Ala-Asp) box helicase 56	0.49
Derl3	derlin 3	0.33
Dfna5	deafness, autosomal dominant 5 (human)	0.50
Dfnb59	deafness, autosomal recessive 59	0.26
Dhcr7	7-dehydrocholesterol reductase	0.57
Dhfr	dihydrofolate reductase	0.44
Dhps	deoxyhypusine synthase	0.55
Dhrs3	dehydrogenase/reductase (SDR family) member 3	0.17
Dhrs4	dehydrogenase/reductase (SDR family) member 4	0.51

Dlx1	distal-less homeobox 1	0.55
Dnajc14	DnaJ (Hsp40) homolog, subfamily C, member 14	0.50
Dnajc5	DnaJ (Hsp40) homolog, subfamily C, member 5	0.53
Dnal1	dynein, axonemal, light chain 1	0.47
Dner	delta/notch-like EGF repeat containing	0.53
Dnm1l	dynamin 1-like	0.53
Dok4	docking protein 4	0.54
Dpep1	dipeptidase 1 (renal)	0.35
Dscc1	defective in sister chromatid cohesion 1 homolog (S. cerevisiae)	0.54
Dse	dermatan sulfate epimerase	0.53
Dusp1	dual specificity phosphatase 1	0.44
Dut	deoxyuridine triphosphatase	0.57
Dvl3	dishevelled, dsh homolog 3 (Drosophila)	0.57
Dynll1	dynein light chain LC8-type 1	0.53
Efcab11	EF-hand calcium binding domain 11	0.49
Efs	embryonal Fyn-associated substrate	0.54
Eif3e	eukaryotic translation initiation factor 3, subunit E	0.55
Eif3g	eukaryotic translation initiation factor 3, subunit G	0.48
Elovl6	ELOVL fatty acid elongase 6	0.37
Elovl6	ELOVL fatty acid elongase 6	0.26
Elp6	elongator acetyltransferase complex subunit 6	0.56
Emg1	EMG1 nucleolar protein homolog (S. cerevisiae)	0.39
Emg1	EMG1 nucleolar protein homolog (S. cerevisiae)	0.45
Emp3	epithelial membrane protein 3	0.53
Eno1	enolase 1, (alpha)	0.50
Epha4	Eph receptor A4	0.48
Epha4	Eph receptor A4	0.48
Etfa	electron-transfer-flavoprotein, alpha polypeptide	0.47
Exoc4	exocyst complex component 4	0.55
Exoc5	exocyst complex component 5	0.55
Exoc7	exocyst complex component 7	0.55
Fa2h	fatty acid 2-hydroxylase	0.39
Fads1	fatty acid desaturase 1	0.37
Fads2	fatty acid desaturase 2	0.25
Fam173a	family with sequence similarity 173, member A	0.49
Fam18b2	family with sequence similarity 18, member B2	0.53
Fam203a	family with sequence similarity 203, member A	0.47
Fam213a	family with sequence similarity 213, member A	0.38
Fam64a	family with sequence similarity 64, member A	0.45
Fam83e	family with sequence similarity 83, member E	0.38
Faslg	Fas ligand (TNF superfamily, member 6)	0.34
Fasn	fatty acid synthase	0.54
Fat2	FAT tumor suppressor homolog 2 (Drosophila)	0.03
Fat4	FAT tumor suppressor homolog 4 (Drosophila)	0.32
Fbxo3	F-box protein 3	0.49

Fbxw9	F-box and WD repeat domain containing 9	0.49
Fdps	farnesyl diphosphate synthase	0.21
Fdps	farnesyl diphosphate synthase	0.25
Fert2	fer (fms/fps related) protein kinase, testis specific 2	0.46
Filip1l	filamin A interacting protein 1-like	0.28
Fopnl	FGFR1OP N-terminal like	0.57
Fos	FBJ osteosarcoma oncogene	0.45
Foxc1	forkhead box C1	0.41
Foxq1	forkhead box Q1	0.33
Fscn2	fascin homolog 2, actin-bundling protein, retinal (Strongylocentrotus purpuratus)	0.44
Fstl1	follistatin-like 1	0.44
Fut2	fucosyltransferase 2 (secretor status included)	0.02
Fzd2	frizzled family receptor 2	0.46
Gabrb3	gamma-aminobutyric acid (GABA) A receptor, beta 3	0.02
Gadd45g	growth arrest and DNA-damage-inducible, gamma	0.41
Gale	UDP-galactose-4-epimerase	0.38
Gapdh-ps1	glyceraldehyde-3-phosphate dehydrogenase, pseudogene 1	0.55
Ggct	gamma-glutamyl cyclotransferase	0.47
Gja1	gap junction protein, alpha 1	0.54
Glb1l	galactosidase, beta 1-like	0.39
Glipr2	GLI pathogenesis-related 2	0.44
Glipr2	GLI pathogenesis-related 2	0.48
Glod4	glyoxalase domain containing 4	0.55
Gmcl1	germ cell-less, spermatogenesis associated 1	0.47
Gmfb	glia maturation factor, beta	0.37
Gmppb	GDP-mannose pyrophosphorylase B	0.34
Gmpr2	guanosine monophosphate reductase 2	0.48
Gng12	guanine nucleotide binding protein (G protein), gamma 12	0.47
Gpn1	GPN-loop GTPase 1	0.55
Gpn1	GPN-loop GTPase 1	0.57
Gpx7	glutathione peroxidase 7	0.43
Gramd1b	GRAM domain containing 1B	0.48
Grik3	glutamate receptor, ionotropic, kainate 3	0.01
Grwd1	glutamate-rich WD repeat containing 1	0.57
Gspt2	G1 to S phase transition 2	0.02
Gtse1	G-2 and S-phase expressed 1	0.41
H2afz	H2A histone family, member Z	0.48
H3f3b	H3 histone, family 3B	0.55
Haus1	HAUS augmin-like complex, subunit 1	0.48
Hccs	holocytochrome c synthase	0.47
Hcfc1	host cell factor C1	0.53
Hdac6	histone deacetylase 6	0.53
Hdgf	hepatoma-derived growth factor	0.56
Heatr2	HEAT repeat containing 2	0.37
Hif1a	hypoxia-inducible factor 1, alpha subunit (basic helix-loop-helix transcription factor)	0.51

Hint2	histidine triad nucleotide binding protein 2	0.57
Hist1h1a	histone cluster 1, H1a	0.18
Hist1h2ai	histone cluster 1, H2ai	0.43
Hist1h2ail	histone cluster 1, H2ai-like	0.50
Hist1h2an	histone cluster 1, H2an	0.16
Hist1h2an	histone cluster 1, H2an	0.39
Hist1h2bc	histone cluster 1, H2bc	0.26
Hist1h2bc	histone cluster 1, H2bc	0.30
Hist1h2bd	histone cluster 1, H2bd	0.30
Hist1h2bf	histone cluster 1, H2bf	0.13
Hist1h2bh	histone cluster 1, H2bh	0.15
Hist1h2bh	histone cluster 1, H2bh	0.13
Hist1h2bl	histone cluster 1, H2bl	0.32
Hist1h4b	histone cluster 1, H4b	0.51
Hist1h4m	histone cluster 1, H4m	0.52
Hist1h4m	histone cluster 1, H4m	0.49
Hist2h3c2	histone cluster 2, H3c2	0.48
Hist2h4	histone cluster 2, H4	0.55
Hist3h2a	histone cluster 3, H2a	0.33
Hist3h2ba	histone cluster 3, H2ba	0.42
Hist3h2bb	histone cluster 3, H2bb	0.35
Hm13	histocompatibility 13	0.48
Hmbox1	homeobox containing 1	0.04
Hmg1l1	high-mobility group (nonhistone chromosomal) protein 1-like 1	0.43
Hmgcr	3-hydroxy-3-methylglutaryl-CoA reductase	0.51
Hmgcs1	3-hydroxy-3-methylglutaryl-CoA synthase 1 (soluble)	0.44
Hnrnpa2b1	heterogeneous nuclear ribonucleoprotein A2/B1	0.56
Hnrnpa3	heterogeneous nuclear ribonucleoprotein A3	0.57
Hnrnpm	heterogeneous nuclear ribonucleoprotein M	0.53
Hnrpd	heterogeneous nuclear ribonucleoprotein D	0.55
Hnrph1	heterogeneous nuclear ribonucleoprotein H1	0.43
Hnrph1	heterogeneous nuclear ribonucleoprotein H1	0.53
Hpx	hemopexin	0.38
Hras	Harvey rat sarcoma virus oncogene	0.52
Hsd17b12	hydroxysteroid (17-beta) dehydrogenase 12	0.39
Hsd17b7	hydroxysteroid (17-beta) dehydrogenase 7	0.44
Hspa5	heat shock protein 5	0.57
Hspa8	heat shock 70kDa protein 8	0.44
Hspa8	heat shock 70kDa protein 8	0.47
Hspbp1	HSPA binding protein, cytoplasmic cochaperone 1	0.56
Hypk	Huntingtin interacting protein K	0.57
Id1	inhibitor of DNA binding 1	0.29
Id2	inhibitor of DNA binding 2	0.18
Id3	inhibitor of DNA binding 3	0.22
Idi1	isopentenyl-diphosphate delta isomerase 1	0.48

Ier3	immediate early response 3	0.51
Ier5l	immediate early response 5-like	0.51
Ifi35	interferon-induced protein 35	0.33
Ift122	intraflagellar transport 122 homolog (Chlamydomonas)	0.56
Igtp	interferon gamma induced GTPase	0.55
Ikbip	IKBKB interacting protein	0.42
Imp3	IMP3, U3 small nucleolar ribonucleoprotein, homolog (yeast)	0.54
Insig1	insulin induced gene 1	0.20
Ints2	integrator complex subunit 2	0.51
Irf2	interferon regulatory factor 2	0.06
Itfg2	integrin alpha FG-GAP repeat containing 2	0.45
Itgb6	integrin, beta 6	0.54
Ivd	isovaleryl-CoA dehydrogenase	0.56
Junb	jun B proto-oncogene	0.37
Kalrn	kalirin, RhoGEF kinase	0.56
Kcnk12	potassium channel, subfamily K, member 12	0.35
Kdelr2	KDEL (Lys-Asp-Glu-Leu) endoplasmic reticulum protein retention receptor 2	0.47
Khnyn-ps1	KH and NYN domain containing, pseudogene 1	0.55
Klc3	kinesin light chain 3	0.50
Klhl13	kelch-like 13 (Drosophila)	0.49
Kng1	kininogen 1	0.17
Kng1l1	kininogen 1-like 1	0.17
Kpna5	karyopherin alpha 5 (importin alpha 6)	0.51
Kptn	kaptin (actin binding protein)	0.55
L3hypdh	L-3-hydroxyproline dehydratase (trans-)	0.55
Laptm4a	lysosomal protein transmembrane 4 alpha	0.55
Lbp	lipopolysaccharide binding protein	0.26
Ldha	lactate dehydrogenase A	0.54
Ldlr	low density lipoprotein receptor	0.38
Lipe	lipase, hormone sensitive	0.36
Lmod2	leiomodin 2 (cardiac)	0.38
Lox	lysyl oxidase	0.32
Lpar6	lysophosphatidic acid receptor 6	0.39
Lrp2	low density lipoprotein receptor-related protein 2	0.41
Lrrc48	leucine rich repeat containing 48	0.44
Lsm4	LSM4 homolog, U6 small nuclear RNA associated (S. cerevisiae)	0.48
Lyrm2	LYR motif containing 2	0.53
Mad2l2	MAD2 mitotic arrest deficient-like 2 (yeast)	0.43
Magt1	magnesium transporter 1	0.40
Map4k3	mitogen-activated protein kinase kinase kinase kinase 3	0.47
Map7d2	MAP7 domain containing 2	0.14
Mat2a	methionine adenosyltransferase II, alpha	0.56
Mbd3	methyl-CpG binding domain protein 3	0.52
Mdh1	malate dehydrogenase 1, NAD (soluble)	0.54
Med29	mediator complex subunit 29	0.45

Mef2c	myocyte enhancer factor 2C	0.10
Mfap2	microfibrillar-associated protein 2	0.03
Mkks	McKusick-Kaufman syndrome	0.38
Mlec	malectin	0.49
Mmadhc	methylmalonic aciduria (cobalamin deficiency) cblD type, with homocystinuria	0.50
Mmp14	matrix metallopeptidase 14 (membrane-inserted)	0.11
Mnd1	meiotic nuclear divisions 1 homolog (S. cerevisiae)	0.51
Mon2	MON2 homolog (S. cerevisiae)	0.50
Morf4l1	mortality factor 4 like 1	0.41
Mpp6	membrane protein, palmitoylated 6 (MAGUK p55 subfamily member 6)	0.55
Mpst	mercaptopyruvate sulfurtransferase	0.56
Mrpl35	mitochondrial ribosomal protein L35	0.52
Mrpl49	mitochondrial ribosomal protein L49	0.44
Mrps10	mitochondrial ribosomal protein S10	0.11
Msmo1	methylsterol monooxygenase 1	0.37
Mst1	Macrophage stimulating 1 (hepatocyte growth factor-like)	0.41
Msx2	msh homeobox 2	0.49
Msx2	msh homeobox 2	0.46
Mvd	mevalonate (diphospho) decarboxylase	0.53
Mvk	mevalonate kinase	0.50
Mvp	major vault protein	0.45
Mxra8	matrix-remodelling associated 8	0.56
Myl12a	myosin, light chain 12A, regulatory, non-sarcomeric	0.57
Myl12a	myosin, light chain 12A, regulatory, non-sarcomeric	0.52
Myl12b	myosin, light chain 12B, regulatory	0.44
Myl4	myosin, light chain 4	0.24
Mylpf	myosin light chain, phosphorylatable, fast skeletal muscle	0.52
N6amt1	N-6 adenine-specific DNA methyltransferase 1 (putative)	0.44
Ncaph2	non-SMC condensin II complex, subunit H2	0.33
Nfkb1	nuclear factor of kappa light polypeptide gene enhancer in B-cells 1	0.55
Nfkbia	nuclear factor of kappa light polypeptide gene enhancer in B-cells inhibitor, alpha	0.51
Nhp2	NHP2 ribonucleoprotein	0.54
Nim1	serine/threonine-protein kinase NIM1	0.09
Nipa2	non imprinted in Prader-Willi/Angelman syndrome 2	0.56
Nme7	NME/NM23 family member 7	0.48
Nmnat1	nicotinamide nucleotide adenylyltransferase 1	0.55
Npepo	aminopeptidase O	0.51
Npm3	nucleophosmin/nucleoplasmin, 3	0.50
Nr4a3	nuclear receptor subfamily 4, group A, member 3	0.33
Nrep	neuronal regeneration related protein	0.44
Nrep	neuronal regeneration related protein	0.39
Nsdhl	NAD(P) dependent steroid dehydrogenase-like	0.46
Ntn4	netrin 4	0.31
Nudcd2	NudC domain containing 2	0.46
Nudcd2	NudC domain containing 2	0.42

Nudt14	nudix (nucleoside diphosphate linked moiety X)-type motif 14	0.47
Oaz2	ornithine decarboxylase antizyme 2	0.57
Obsl1	obscurin-like 1	0.37
Odf1	outer dense fiber of sperm tails 1	0.02
Ogdh	oxoglutarate (alpha-ketoglutarate) dehydrogenase (lipoamide)	0.56
Oip5	Opa interacting protein 5	0.31
Olr540	olfactory receptor 540	0.02
Olr691	olfactory receptor 691	0.02
Olr834	olfactory receptor 834	0.05
Oscp1	organic solute carrier partner 1	0.54
Pabpn1l	poly(A)binding protein nuclear 1-like	0.03
Pafah1b2	platelet-activating factor acetylhydrolase 1b, catalytic subunit 2	0.57
Paip2b	poly(A) binding protein interacting protein 2B	0.57
Parn	poly(A)-specific ribonuclease	0.42
Parva	parvin, alpha	0.55
Pbk	PDZ binding kinase	0.55
Pbx1	pre-B-cell leukemia homeobox 1	0.48
Pcbd1	pterin-4 alpha-carbinolamine dehydratase/dimerization cofactor of hepatocyte nuclear factor 1 alpha	0.44
Pcbd1	pterin-4 alpha-carbinolamine dehydratase/dimerization cofactor of hepatocyte nuclear factor 1 alpha	0.50
Pcca	propionyl-coenzyme A carboxylase, alpha polypeptide	0.42
Pcsk9	proprotein convertase subtilisin/kexin type 9	0.23
Pdgfc	platelet derived growth factor C	0.07
Pdhb	pyruvate dehydrogenase (lipoamide) beta	0.47
Pdk1	pyruvate dehydrogenase kinase, isozyme 1	0.55
Pdzk1ip1	PDZK1 interacting protein 1	0.50
Peg12	paternally expressed 12	0.34
Pex11g	peroxisomal biogenesis factor 11 gamma	0.43
Pgk1	phosphoglycerate kinase 1	0.57
Pgp	phosphoglycolate phosphatase	0.47
Phf19	PHD finger protein 19	0.56
Pias1	protein inhibitor of activated STAT, 1	0.32
Pibf1	progesterone immunomodulatory binding factor 1	0.57
Pigw	phosphatidylinositol glycan anchor biosynthesis, class W	0.44
Pigz	phosphatidylinositol glycan anchor biosynthesis, class Z	0.55
Pih1d1	PIH1 domain containing 1	0.46
Pih1d2	PIH1 domain containing 2	0.56
Pitpna	phosphatidylinositol transfer protein, alpha	0.53
Pkd2	polycystic kidney disease 2 (autosomal dominant)	0.40
Pkdcc	protein kinase domain containing, cytoplasmic	0.48
Plag1	pleiomorphic adenoma gene 1	0.55
Plgrkt	plasminogen receptor, C-terminal lysine transmembrane protein	0.57
Plscr1	phospholipid scramblase 1	0.41
Pmpca	peptidase (mitochondrial processing) alpha	0.54

Pnrc1	proline-rich nuclear receptor coactivator 1	0.47
Pnrc2	proline-rich nuclear receptor coactivator 2	0.50
Pof1b	premature ovarian failure 1B	0.22
Pofut1	protein O-fucosyltransferase 1	0.45
Pole3	polymerase (DNA directed), epsilon 3, accessory subunit	0.45
Polr3f	polymerase (RNA) III (DNA directed) polypeptide F	0.54
Polr3h	polymerase (RNA) III (DNA directed) polypeptide H	0.48
Pop5	processing of precursor 5, ribonuclease P/MRP subunit (S. cerevisiae)	0.49
Ppic	peptidylprolyl isomerase C	0.49
Ppih	peptidylprolyl isomerase H (cyclophilin H)	0.56
Ppp1r10	protein phosphatase 1, regulatory subunit 10	0.55
Ppp1r16a	protein phosphatase 1, regulatory subunit 16A	0.47
Ppp1r17	protein phosphatase 1, regulatory subunit 17	0.01
Prdm2	PR domain containing 2, with ZNF domain	0.54
Prpf31	PRP31 pre-mRNA processing factor 31 homolog (S. cerevisiae)	0.51
Prr11	proline rich 11	0.50
Prrx1	paired related homeobox 1	0.56
Prrx1	paired related homeobox 1	0.51
Prtfdc1	phosphoribosyl transferase domain containing 1	0.56
Psma2	proteasome (prosome, macropain) subunit, alpha type 2	0.51
Psmb1	proteasome (prosome, macropain) subunit, beta type 1	0.50
Psmb10	proteasome (prosome, macropain) subunit, beta type 10	0.33
Psmb6	proteasome (prosome, macropain) subunit, beta type 6	0.57
Psmb8	proteasome (prosome, macropain) subunit, beta type 8 (large multifunctional peptidase 7)	0.39
Psmd13	proteasome (prosome, macropain) 26S subunit, non-ATPase, 13	0.55
Psmd14	proteasome (prosome, macropain) 26S subunit, non-ATPase, 14	0.40
Psmd9	proteasome (prosome, macropain) 26S subunit, non-ATPase, 9	0.55
Psmd9	proteasome (prosome, macropain) 26S subunit, non-ATPase, 9	0.51
Psme2	proteasome (prosome, macropain) activator subunit 2	0.55
Ptgis	prostaglandin I2 (prostacyclin) synthase	0.40
Ptprd	protein tyrosine phosphatase, receptor type, D	0.54
Ptrhd1	peptidyl-tRNA hydrolase domain containing 1	0.57
Pycrl	pyrroline-5-carboxylate reductase-like	0.57
Rab25	RAB25, member RAS oncogene family	0.03
Rab2a	RAB2A, member RAS oncogene family	0.57
Rab32	RAB32, member RAS oncogene family	0.38
Rabl5	RAB, member RAS oncogene family-like 5	0.40
Ranbp1	RAN binding protein 1	0.36
Rap1b	RAP1B, member of RAS oncogene family	0.46
Raph1	Ras association (RalGDS/AF-6) and pleckstrin homology domains 1	0.50
Rasl11a	RAS-like family 11 member A	0.26
Rasl12	RAS-like, family 12	0.50
Rasl2-9	RAS-like, family 2, locus 9	0.52
Rbmx	RNA binding motif protein, X-linked	0.47

Rbx1	ring-box 1, E3 ubiquitin protein ligase	0.54
Rcc2	regulator of chromosome condensation 2	0.51
Rgs16	regulator of G-protein signaling 16	0.40
Rmdn2	regulator of microtubule dynamics 2	0.44
Rnf113a1	ring finger protein 113A1	0.52
Rnpep	arginyl aminopeptidase (aminopeptidase B)	0.56
Ropn1l	rhophilin associated tail protein 1-like	0.50
Rpa3	replication protein A3	0.42
Rpf2	ribosome production factor 2 homolog (S. cerevisiae)	0.56
Rps23	ribosomal protein S23	0.57
Rrad	Ras-related associated with diabetes	0.47
Rrm2	ribonucleotide reductase M2	0.39
Rtp4	receptor (chemosensory) transporter protein 4	0.57
Rwdd4	RWD domain containing 4A	0.44
Scd	stearoyl-CoA desaturase (delta-9-desaturase)	0.30
Scd	stearoyl-CoA desaturase (delta-9-desaturase)	0.38
Scd4	stearoyl-coenzyme A desaturase 4	0.26
Scfd2	sec1 family domain containing 2	0.32
Scnm1	sodium channel modifier 1	0.57
Scnn1g	sodium channel, nonvoltage-gated 1, gamma	0.03
Scrn1	secernin 1	0.50
Scrn2	secernin 2	0.44
Sec13	SEC13 homolog (S. cerevisiae)	0.52
Sec61a1	Sec61 alpha 1 subunit (S. cerevisiae)	0.37
Selo	selenoprotein O	0.54
Selt	selenoprotein T	0.35
Sema3c	sema domain, immunoglobulin domain (Ig), short basic domain, secreted, (semaphorin) 3C	0.57
Sema4a	sema domain, immunoglobulin domain (Ig), transmembrane domain (TM) and short cytoplasmic domain, (semaphorin) 4A	0.50
Senp7	SUMO1/sentrin specific peptidase 7	0.57
Sephs1	selenophosphate synthetase 1	0.52
Sfr1	SWI5-dependent recombination repair 1	0.47
Shbg	sex hormone binding globulin	0.28
Shc3	SHC (Src homology 2 domain containing) transforming protein 3	0.21
Shisa3	shisa homolog 3 (Xenopus laevis)	0.23
Siglec1	sialic acid binding Ig-like lectin 1, sialoadhesin	0.11
Skiv2l	superkiller viralicidic activity 2-like (S. cerevisiae)	0.51
Slc10a1	solute carrier family 10 (sodium/bile acid cotransporter family), member 1	0.02
Slc10a7	solute carrier family 10 (sodium/bile acid cotransporter family), member 7	0.45
Slc11a2	solute carrier family 11 (proton-coupled divalent metal ion transporters), member 2	0.51
Slc11a2	solute carrier family 11 (proton-coupled divalent metal ion transporters), member 2	0.42
Slc16a13	solute carrier family 16, member 13 (monocarboxylic acid transporter 13)	0.56
Slc16a3	solute carrier family 16, member 3 (monocarboxylic acid transporter 4)	0.49
Slc24a3	solute carrier family 24 (sodium/potassium/calcium exchanger), member 3	0.35

Slc25a20	solute carrier family 25 (carnitine/acylcarnitine translocase), member 20	0.56
Slc26a4	solute carrier family 26, member 4	0.53
Slc29a3	solute carrier family 29 (nucleoside transporters), member 3	0.38
Slc35a2	solute carrier family 35 (UDP-galactose transporter), member A2	0.52
Slc37a4	solute carrier family 37 (glucose-6-phosphate transporter), member 4	0.51
Slc38a4	solute carrier family 38, member 4	0.30
Slc39a7	solute carrier family 39 (zinc transporter), member 7	0.48
Slc51b	solute carrier family 51, beta subunit	0.50
Slc52a2	solute carrier family 52, riboflavin transporter, member 2	0.45
Smim1	small integral membrane protein 1	0.54
Snapap	SNAP-associated protein	0.55
Snrpc	small nuclear ribonucleoprotein polypeptide C	0.49
Snx11	sorting nexin 11	0.55
Socs3	suppressor of cytokine signaling 3	0.48
Sod2	superoxide dismutase 2, mitochondrial	0.50
Sostdc1	sclerostin domain containing 1	0.05
Spa17	sperm autoantigenic protein 17	0.46
Sparc	secreted protein, acidic, cysteine-rich (osteonectin)	0.43
Spata24	spermatogenesis associated 24	0.48
Spcs3	signal peptidase complex subunit 3 homolog (S. cerevisiae)	0.53
Sqle	squalene epoxidase	0.42
Srebf1	sterol regulatory element binding transcription factor 1	0.52
Srebf2	sterol regulatory element binding transcription factor 2	0.55
Srsf1	serine/arginine-rich splicing factor 1	0.39
Srsf1	serine/arginine-rich splicing factor 1	0.39
Srsf10	serine/arginine-rich splicing factor 10	0.45
Srsf2	serine/arginine-rich splicing factor 2	0.46
Srsf3	serine/arginine-rich splicing factor 3	0.44
Srsf7	serine/arginine-rich splicing factor 7	0.57
Ssr1	signal sequence receptor, alpha	0.54
Stard4	StAR-related lipid transfer (START) domain containing 4	0.33
Stt3b	STT3, subunit of the oligosaccharyltransferase complex, homolog B (S. cerevisiae)	0.55
Sub1	SUB1 homolog (S. cerevisiae)	0.48
Sucla2	succinate-CoA ligase, ADP-forming, beta subunit	0.46
Syne4	spectrin repeat containing, nuclear envelope family member 4	0.21
Taf10	TAF10 RNA polymerase II, TATA box binding protein (TBP)-associated factor	0.40
Taf9b	TAF9B RNA polymerase II, TATA box binding protein (TBP)-associated factor	0.51
Tagln	transgelin	0.53
Tax1bp3	Tax1 (human T-cell leukemia virus type I) binding protein 3	0.48
Tbc1d7	TBC1 domain family, member 7	0.49
Tceanc	transcription elongation factor A (SII) N-terminal and central domain containing	0.55
Tcf25	transcription factor 25 (basic helix-loop-helix)	0.50
Tead1	TEA domain family member 1	0.57
Tecpr1	tectonin beta-propeller repeat containing 1	0.33
Ten1	TEN1 telomerase capping complex subunit	0.36

Tex19.1	testis expressed 19.1	0.37
Tex26	testis expressed 26	0.39
Tgm1	transglutaminase 1 (K polypeptide epidermal type I, protein-glutamine-gamma-glutamyltransferase)	0.50
Tgm6	transglutaminase 6	0.44
Thap7	THAP domain containing 7	0.53
Tia1	TIA1 cytotoxic granule-associated RNA binding protein	0.43
Timm8b	translocase of inner mitochondrial membrane 8 homolog b (yeast)	0.55
Tk1	thymidine kinase 1, soluble	0.48
Tle1	transducin-like enhancer of split 1 (E(sp1) homolog, Drosophila)	0.47
Tlr2	toll-like receptor 2	0.38
Tm9sf4	transmembrane 9 superfamily protein member 4	0.54
Tmem128	transmembrane protein 128	0.57
Tmem18	transmembrane protein 18	0.54
Tmem183a	transmembrane protein 183A	0.54
Tmem208	transmembrane protein 208	0.49
Tmem223	transmembrane protein 223	0.45
Tmem223	transmembrane protein 223	0.45
Tmem30a	transmembrane protein 30A	0.56
Tmem97	transmembrane protein 97	0.49
Tnnc1	troponin C type 1 (slow)	0.25
Tp53inp2	tumor protein p53 inducible nuclear protein 2	0.34
Tpi1	triosephosphate isomerase 1	0.53
Tpi1	triosephosphate isomerase 1	0.54
Tpm1	tropomyosin 1, alpha	0.47
Tprkb	Tp53rk binding protein	0.52
Tra2b	transformer 2 beta homolog (Drosophila)	0.37
Trim47	tripartite motif-containing 47	0.57
Trps1	trichorhinophalangeal syndrome I	0.55
Trpt1	tRNA phosphotransferase 1	0.56
Tsta3	tissue specific transplantation antigen P35B	0.38
Ttc22	tetratricopeptide repeat domain 22	0.34
Tuba1a	tubulin, alpha 1A	0.33
Tuba1b	tubulin, alpha 1B	0.50
Tuba1c	tubulin, alpha 1C	0.44
Tuba1c	tubulin, alpha 1C	0.44
Tuba3a	tubulin, alpha 3A	0.53
Tubgcp4	tubulin, gamma complex associated protein 4	0.57
Tyms	thymidylate synthetase	0.53
Ube2i	ubiquitin-conjugating enzyme E2I	0.57
Ube2k	ubiquitin-conjugating enzyme E2K	0.52
Ube2l6	ubiquitin-conjugating enzyme E2L 6	0.40
Ube2v1	ubiquitin-conjugating enzyme E2 variant 1	0.57
Ubl4a	ubiquitin-like 4A	0.44
Ubox5	U-box domain containing 5	0.49

Ufd1l	ubiquitin fusion degradation 1 like (yeast)	0.44
Ufm1	ubiquitin-fold modifier 1	0.52
Ulk2	Unc-51 like kinase 2 (C. elegans)	0.42
Unc50	unc-50 homolog (C. elegans)	0.51
Use1	unconventional SNARE in the ER 1 homolog (S. cerevisiae)	0.51
Usp4	ubiquitin specific peptidase 4 (proto-oncogene)	0.56
Usp9x	ubiquitin specific peptidase 9, X-linked	0.52
Uxs1	UDP-glucuronate decarboxylase 1	0.49
Vars	valyl-tRNA synthetase	0.54
Vav3	vav 3 guanine nucleotide exchange factor	0.47
Vcp	valosin-containing protein	0.57
Vimp	VCP-interacting membrane protein	0.38
Vps45	vacuolar protein sorting 45 homolog (S. cerevisiae)	0.52
Wbscr22	Williams Beuren syndrome chromosome region 22	0.51
Wdr18	WD repeat domain 18	0.37
Wdr35l	WD repeat domain 35-like	0.49
Wdr78	WD repeat domain 78	0.40
Wls	wntless homolog (Drosophila)	0.52
Wnt2	wingless-type MMTV integration site family member 2	0.32
Ybey	ybeY metallopeptidase	0.45
Yif1a	Yip1 interacting factor homolog A (S. cerevisiae)	0.45
Yif1b	Yip1 interacting factor homolog B (S. cerevisiae)	0.56
Yipf4	Yip1 domain family, member 4	0.56
Yme1l1	YME1-like 1 (S. cerevisiae)	0.43
Ywhaz	tyrosine 3-monooxygenase/tryptophan 5-monooxygenase activation protein, zeta polypeptide	0.49
Zbtb1	zinc finger and BTB domain containing 1	0.46
Zbtb6	zinc finger and BTB domain containing 6	0.57
Zdhhc20	zinc finger, DHHC-type containing 20	0.45
Zfp207	zinc finger protein 207	0.50
Zfp7	zinc finger protein 7	0.30
Zic2	Zic family member 2	0.31
Zic5	Zic family member 5	0.42
Zmat2	zinc finger, matrin type 2	0.29

Legend to Figures

Supplementary figure 1: Characterization of Mn/ MPP$^+$ and 3-NPA neurtoxicity in N27 cells. N27 cells were exposed to increasing concentrations of 3-NPA (0-8 mM) for 24 h (A) and 48 h (B) and its toxicity was assessed by measuring the cell viability by MTT dye reduction assay. Cell viability assay revealed a dose and time dependent increase in cell death upon 3-NPA treatment. Similarly, N27 cells treated with Mn (C) and MPP$^+$ (D) also showed concentration-dependent increase in cell death at 48 h. LD_{50} concentration of 3-NPA/ Mn/ MPP$^+$ was found to be 4 mM, 150 µM and 250 µM respectively (n= 6 trials per experiment; *p< 0.05, **p < 0.01, ***p< 0.001, ****p< 0.0001). Assessment of gross morphology of N27 cells treated with 2 mM and 4 mM 3-NPA for 48h. N27 cells were cultured on PLL coated coverslips and treated with 3-NPA at LD_{50} and subjected to H & E staining. Representative images of control and 3-NPA treated N27 cells taken at 20X magnification (E) showed prominent changes including loss of cellular processes accompanied by nuclear condensation along with flattening of cells in comparison with their respective control (n= 3 coverslips per group). The insets correspond to magnified images from a field of the same plate but different from the one shown in the 20X image.

Supplementary figure 2. Mitochondrial dysfunction in 3-NPA treated N27 cells. 3-NPA induced reduction in mitochondrial CII activity (100 % activity= 15µg/µL/min) (A) as measured by reduction in absorbance of DCIP at 600nm, along with increased ADP/ ATP (B) and NAD/ NADH (D) at LD_{50}. Dose dependent decrease in mitochondrial membrane potential as demonstrated by JC1 assay also indicated compromised mitochondrial and cellular energy status upon 3-NPA treatment in N27 cells (C) (n= 6 trials per experiment; *p< 0.05, **p < 0.01, ***p< 0.001).

Supplementary figure 3. Ultrastructural evidences of mitochondrial dysfunction in the 3-NPA cell model. In comparison to untreated N27 cells that showed normal mitochondrial morphology (A-i), 3-NPA treated cells showed morphological hallmarks of mitochondrial dysfunction at both 2 mM (B-i) and 4 mM concentrations (C-i, ii). Mitochondrial dysfunction was predominantly characterized by presence of elongated mitochondria (B-i) at 2mM 3-NPA, enlarged mitochondria with abnormal cristae (C-i). 3-NPA treatment also showed extensive vacuolations (B-ii, C-ii) (n= 3 experiments per group). The arrows in the representative electron micrographs depict the described pathological features.

Supplementary figure 4. Assessment of oxidative stress and antioxidant response induced in 3-NPA treated N27 cells. 3-NPA showed concentration dependent increase in formation of ROS in terms of total hydroperoxides both at 24 h (A) and 48 h (B) of treatment highlighting its dose and time dependence along with increased levels of NO released from the cells (C), both of which were measured according to the manufacturer's instructions. Compromised glutathione dynamics as measured by OPT method, was indicated by decrease in GSH/ GSSG with increasing concentration and time (D- 24 h; E- 48 h) (n= 6 trials per experiment; *$p< 0.05$, **$p < 0.01$, ***$p< 0.001$, ****$p< 0.0001$).

Supplementary figure 5. Assessment of quality of the microarray data. mRNA from control and 3-NPA treated N27 cells was utilized for microarray analysis following its hybridization with rat whole genome array (Agilent technologies). Following hybridization, the array was scanned for differences in fluorescence intensities between the control and treatment groups and their qualitative differences were a direct indication of the gene expression. The box plots (A) showing normalized intensity across the duplicates, and principle component analysis (PCA) (D) revealed the data to be of high quality with reproducible results across duplicates. Fold change analysis is represented in the form of box plot (B) showing the median fold change in 3-NPA treatment and volcano plot depicting the upregulated (red) and downregulated (green) genes (C) (n= 2 replicates per group).

Supplementary figure 6. Functional clustering of genes across Mn/ MPP$^+$/ 3-NPA treatment groups. Kmean clustering of dataset from Mn (A), MPP$^+$ (B) and 3-NPA (C) treatment showed maximum number of genes in CL6, CL3 and CL3 respectively, indicating the exclusivity of the dataset in 3-NPA treatment. Differentially expressed genes across the three groups as a fraction of their variability plotted as a Pareto graph showed 3-NPA treatment to have maximum effect in terms of gene expression (D).

Supplementary figure 7. Gene ontology analysis of upregulated (A) and downregulated (B) genes in 3-NPA treated N27 cells showed them to be categorizing into various functional groups including mitochondrial, synaptic and autophagic genes.

Supplementary figure 8. *In silico* analysis and network interpretation of differentially expressed transcripts. Heatmap cluster of drug treated cells according to the pre-defined, 'canonical' sub-systems (A) (generated by MATLAB, version 2015b). Pathway map of the N27 cell line specific Pentose sugar metabolism co-set (generated by Escher; URL- https://escher.github.io.). This pathway was markedly reduced in MPP$^+$ treated cells, differentiating it from the other neurotoxin (and non-treated) N27 cells (B).

Supplementary figure 9. Complete blot of LC3 upon 3-NPA (i, corresponds to figure 4D) and Mn/ MPP$^+$ (ii, corresponds to figure 4D) treatment along with their corresponding replicates (iii). Complete blot of LC3 upon CQ+ 3-NPA co- treatment (iv, corresponds to figure 4F). For most of the blots, care was taken to retain the full length blot for hybridization of antibody and acquisiation of images. In experiments where the primary antibody availability was limited, the nitrocellulose membrane containing the transferred protein was cut based on the molecular weight so as to retain only the areas corresponding to the protiens of interest prior to the treatment with the desired primary antibody or to perform anti β- actin western. The trials correspond to individual experiments (biological replicates). The lanes labeled with text in red have been selected for inclusion in the main figure. The area shown

by red boxes correspond to the respective cropped versions in the main figure.

Supplementary figure 10. Complete blot of p62 in 3-NPA, Mn and MPP^+ treatment (i, corresponds to figure 4I) with their corresponding replicates (iii- iv). Expression level of p62 upon CQ+ 3-NPA co- treatment (v, corresponds to figure 4F) and its replicate (vi). For most of the blots care was taken to retain the full length blot for hybridization of antibody and acquisiation of images. In experiments where the primary antibody availability was limited, the nitrocellulose membrane containing the transferred protein was cut based on the molecular weight so as to retain only the areas corresponding to the protiens of interest prior to the treatment with the desired primary antibody or to perform anti β- actin western. The trials correspond to individual experiments (biological replicates). The lanes labeled with text in red have been selected for inclusion in the main figure. The area shown by red boxes correspond to the respective cropped versions in the main figure.

Supplementary figure 11. Complete blot for LAMP1 (I, corresponds to figure 4K) and its replicate upon Mn/ MPP^+ and 3-NPA treatment. Complete blot depicting the expression levels of AMPK (ii, corresponds to figure 5A), pAMPK (iii, corresponds to figure 5A) and mTOR (iv, corresponds to figure 5A) with their corresponding replicates (C= control, T= 3-NPA treated). For most of the blots care was taken to retain the full length blot for hybridization of antibody and acquisiation of images. In experiments where the primary antibody availability was limited, the nitrocellulose membrane containing the transferred protein was cut based on the molecular weight so as to retain only the areas corresponding to the protiens of interest prior to the treatment with the desired primary antibody or to perform anti β- actin western. The trials correspond to individual experiments (biological replicates). The lanes labeled with text in red have been selected for inclusion in the main figure. The area shown by red boxes correspond to the respective cropped versions in the main figure.

Supplementary figure 12. Complete blot for mTORC1 components and its downstream

targets including RPTOR (i, corresponds to figure 5D), PRAS40 (ii, corresponds to figure 5D), S6K1 (iii, corresponds to figure 5D) and EIF4Ebp1 (iv, corresponds to figure 5D) with their replicates (v) (C= control, T= 3-NPA treated). For most of the blots care was taken to retain the full length blot for hybridization of antibody and acquisiation of images. In experiments where the primary antibody availability was limited, the nitrocellulose membrane containing the transferred protein was cut based on the molecular weight so as to retain only the areas corresponding to the protiens of interest prior to the treatment with the desired primary antibody or to perform anti β- actin western. The trials correspond to individual experiments (biological replicates). The lanes labeled with text in red have been selected for inclusion in the main figure. The area shown by red boxes correspond to the respective cropped versions in the main figure.

Supplementary figure 13. Complete blot depicting the phosphorylation status of mTORC1 components and its downstream targets including pPRAS40 (i, corresponds to figure 5D), pS6K1 (ii, corresponds to figure 5D) and pEIF4Ebp1 (iii) with their replicates (iv, corresponds to figure 5D) (C= control, T= 3-NPA treated). For most of the blots care was taken to retain the full length blot for hybridization of antibody and acquisiation of images. In experiments where the primary antibody availability was limited, the nitrocellulose membrane containing the transferred protein was cut based on the molecular weight so as to retain only the areas corresponding to the protiens of interest prior to the treatment with the desired primary antibody or to perform anti β- actin western. The trials correspond to individual experiments (biological replicates). The lanes labeled with text in red have been selected for inclusion in the main figure. The area shown by red boxes correspond to the respective cropped versions in the main figure.

Supplementary figure 14. Complete blot for mTORC2 components ant its downstream targets including RICTOR (i, corresponds to figure 5G), mSIN1 (ii, corresponds to figure 5G)

and AKT (iii, corresponds to figure 5G) with their respective replicates (C= control, T= 3-NPA treated). For most of the blots care was taken to retain the full length blot for hybridization of antibody and acquisiation of images. In experiments where the primary antibody availability was limited, the nitrocellulose membrane containing the transferred protein was cut based on the molecular weight so as to retain only the areas corresponding to the protiens of interest prior to the treatment with the desired primary antibody or to perform anti β- actin western. The trials correspond to individual experiments (biological replicates). The lanes labeled with text in red have been selected for inclusion in the main figure. The area shown by red boxes correspond to the respective cropped versions in the main figure.

Supplementary figure 15. Complete blot depicting the phosphorylation status of mTORC2 components ant its downstream targets including pmSIN1 (i, corresponds to figure 5G), pAKT(T308) (ii, corresponds to figure 5G) and pAKT(S473) (iii, corresponds to figure 5G) with their respective replicates (C= control, T= 3-NPA treated). For most of the blots care was taken to retain the full length blot for hybridization of antibody and acquisiation of images. In experiments where the primary antibody availability was limited, the nitrocellulose membrane containing the transferred protein was cut based on the molecular weight so as to retain only the areas corresponding to the protiens of interest prior to the treatment with the desired primary antibody or to perform anti β- actin western. The trials correspond to individual experiments (biological replicates). The lanes labeled with text in red have been selected for inclusion in the main figure. The area shown by red boxes correspond to the respective cropped versions in the main figure.

Supplementary figure 16. Complete blot of autopahgic marker BECN1 (i, corresponds to figure 6A) in 3-NPA toxicity along with its dose (ii, corresponds to figure 6D) and time dependence (iv, corresponds to figure 6H) with their respective replicates (iii). Complete blot for caspase 9 with its replicated upon 3-NPA treatment (v, corresponds to figure 6A) (C=

control, T= 3-NPA treated). For most of the blots care was taken to retain the full length blot for hybridization of antibody and acquisiation of images. In experiments where the primary antibody availability was limited, the nitrocellulose membrane containing the transferred protein was cut based on the molecular weight so as to retain only the areas corresponding to the protiens of interest prior to the treatment with the desired primary antibody or to perform anti β- actin western.The trials correspond to individual experiments (biological replicates). The lanes labeled with text in red have been selected for inclusion in the main figure. The area shown by red boxes correspond to the respective cropped versions in the main figure.

Supplementary figure 17. Complete blot for apoptotic markers including BCL2 (i, corresponds to figure 6A) and caspase-3 (vii, corresponds to figure 6A) along with their replicates. Dose and time dependence of expression of apoptotic marker BCL2 (ii- iii, corresponds to figure 6D and H) and autophagic marker p62 (iv, vi) with their replicates (v, corresponds to figure 6D and H) following 3-NPA treatment (C= control, T= 3-NPA treated). For most of the blots care was taken to retain the full length blot for hybridization of antibody and acquisiation of images. In experiments where the primary antibody availability was limited, the nitrocellulose membrane containing the transferred protein was cut based on the molecular weight so as to retain only the areas corresponding to the protiens of interest prior to the treatment with the desired primary antibody or to perform anti β- actin western. The trials correspond to individual experiments (biological replicates). The lanes labeled with text in red have been selected for inclusion in the main figure. The area shown by red boxes correspond to the respective cropped versions in the main figure.

Supplementary figure 18. Complete blot depicting BDNF expression upon 3-NPA treatment in N27 cells (i, corresponds to figure 7A). Expression level of p62 (ii, corresponds to figure 7C) and LAMP1 (iii, corresponds to figure 7C) upon BDNF and 3-NPA co-treatment. Complete blot of AKT (iv, corresponds to figure 7G) and its phosphorylation at S473 (v,

corresponds to figure 7G) during BDNF and 3-NPA co-treatment (C= control, T= 3-NPA treated). For most of the blots care was taken to retain the full length blot for hybridization of antibody and acquisiation of images. In experiments where the primary antibody availability was limited, the nitrocellulose membrane containing the transferred protein was cut based on the molecular weight so as to retain only the areas corresponding to the protiens of interest prior to the treatment with the desired primary antibody or to perform anti β- actin western. The trials correspond to individual experiments (biological replicates). The lanes labeled with text in red have been selected for inclusion in the main figure. The area shown by red boxes correspond to the respective cropped versions in the main figure.

Supplementary figure 19. Representative blots of β- actin depicting equal load used as loading control and for protein normalization (i- ii, correspond to figure 4D; iii correspond to figure 4F; iv correspond to figure 4K, v correspond to figure 4I; vi correspond to figure 5A; vii correspond to figure 5D; viii correspond to figure 5G; ix correspond to figure 6A; x correspond to figure 6D; xi correspond to figure 7A; xii correspond to figure 6H; xiii correspond to figure 7C; xiv correspond to figure 7G) (C= control, T= 3-NPA treated). The images correspond to the nitrocellulose membrane containing the transferred protein which was cut so as to retain only the area corresponding to the protein of interest prior to hybridization with the primary antibody. The trials correspond to individual experiments (biological replicates). The lanes labeled with text in red have been selected for inclusion in the main figure. The area shown by red boxes correspond to the respective cropped versions in the main figure.

Supplementary spreadsheets have been enclosed as a separate MS- Excel file.

Supplementary figure 1

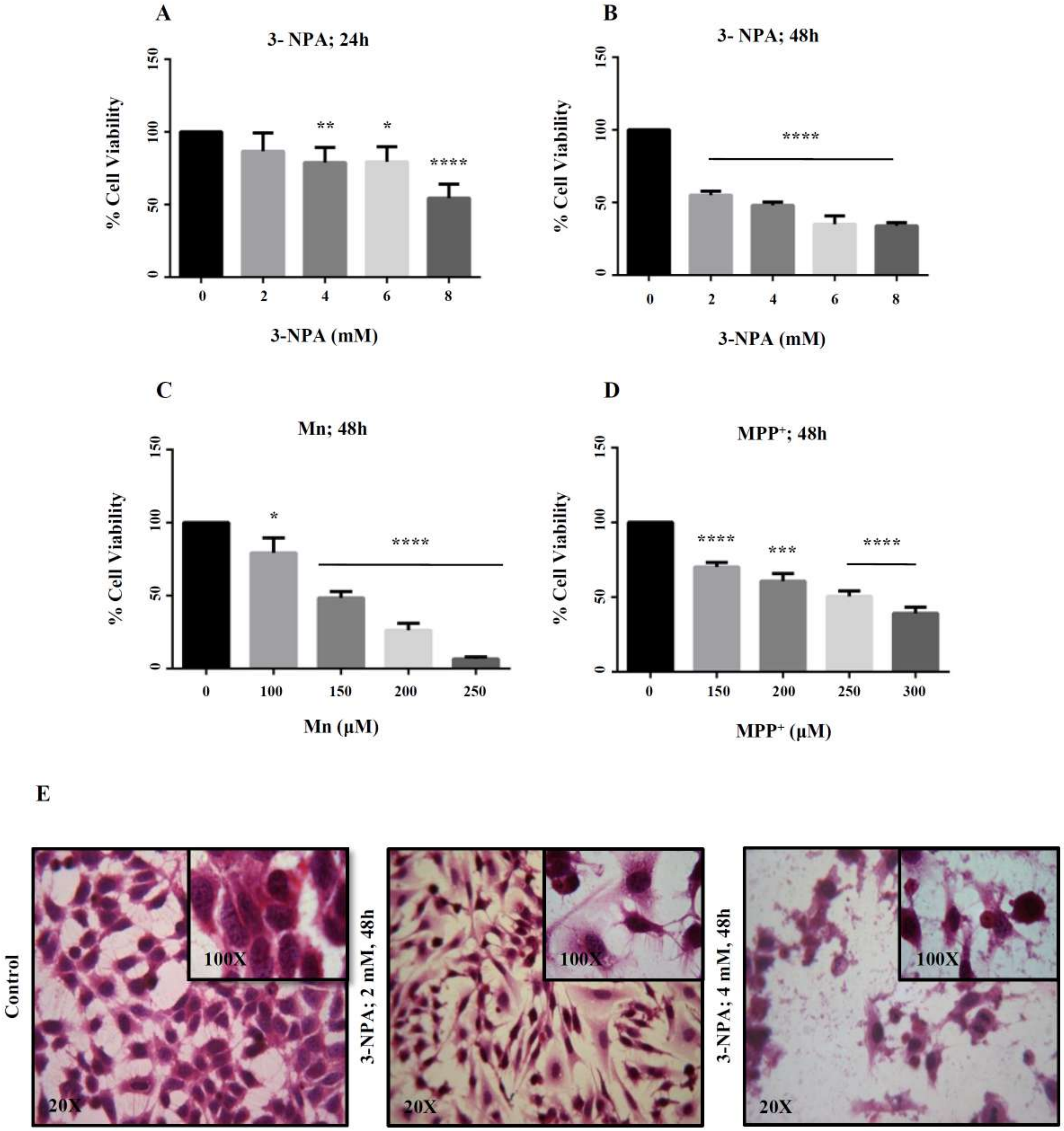

Supplementary figure 2

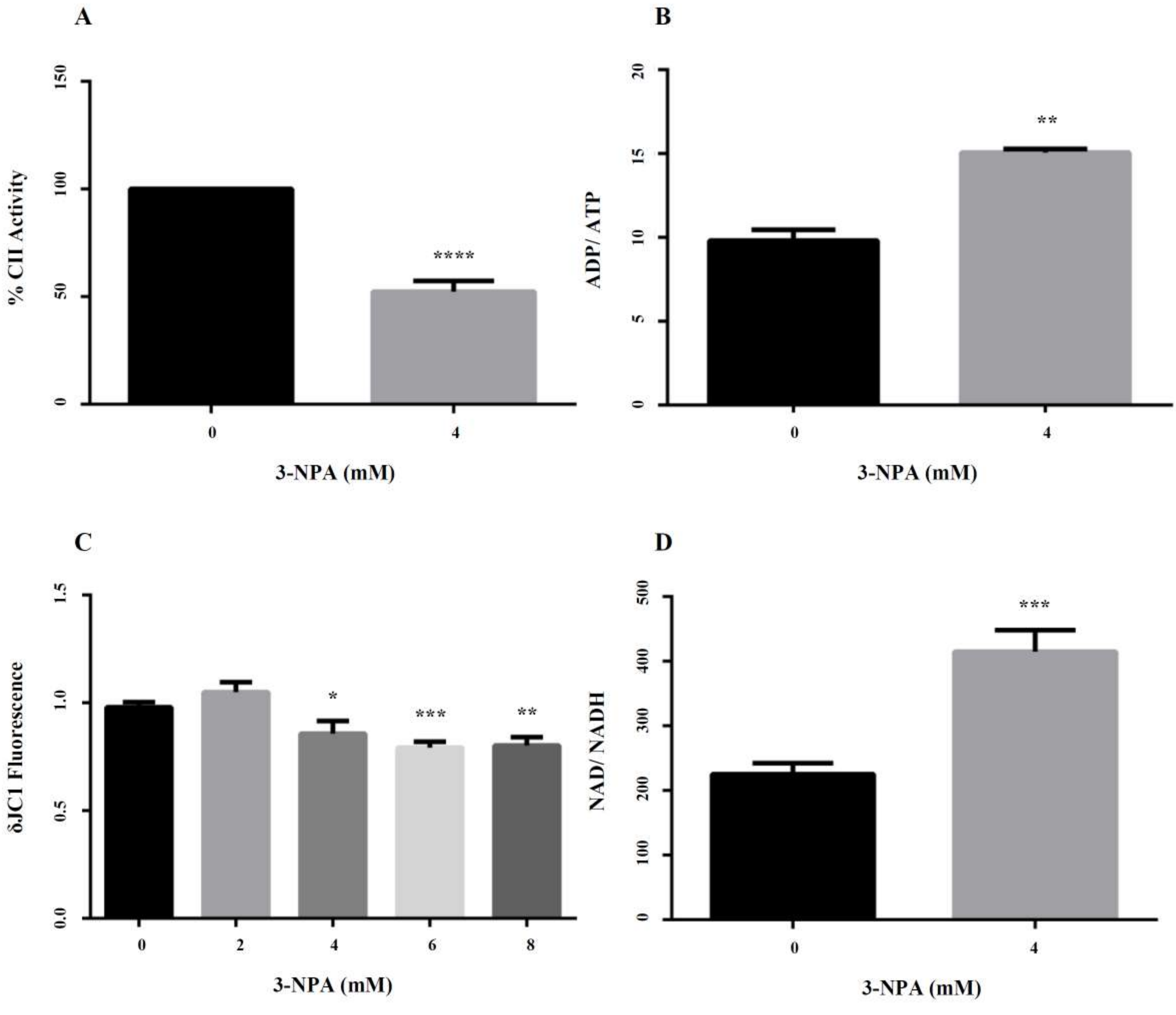

Supplementary figure 3

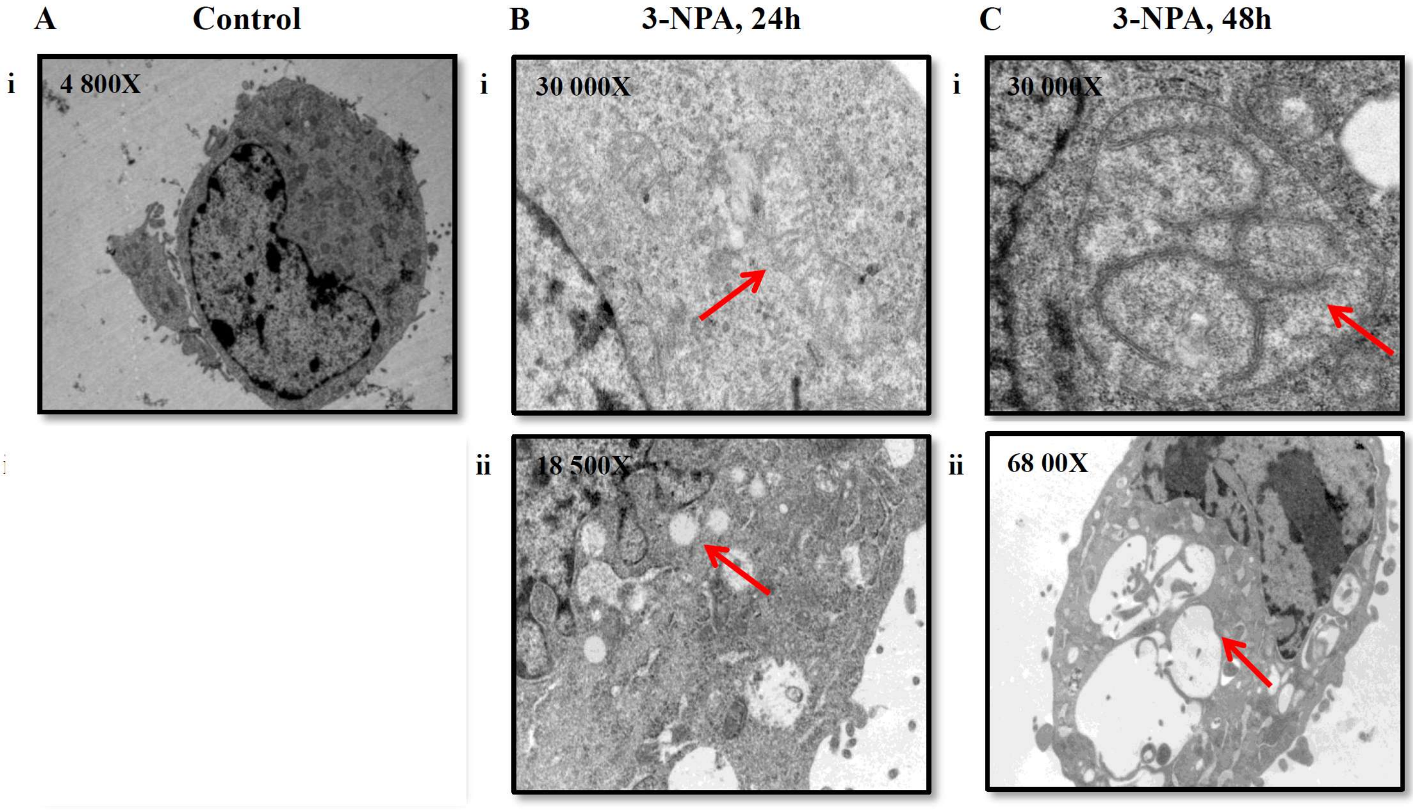

Supplementary figure 4

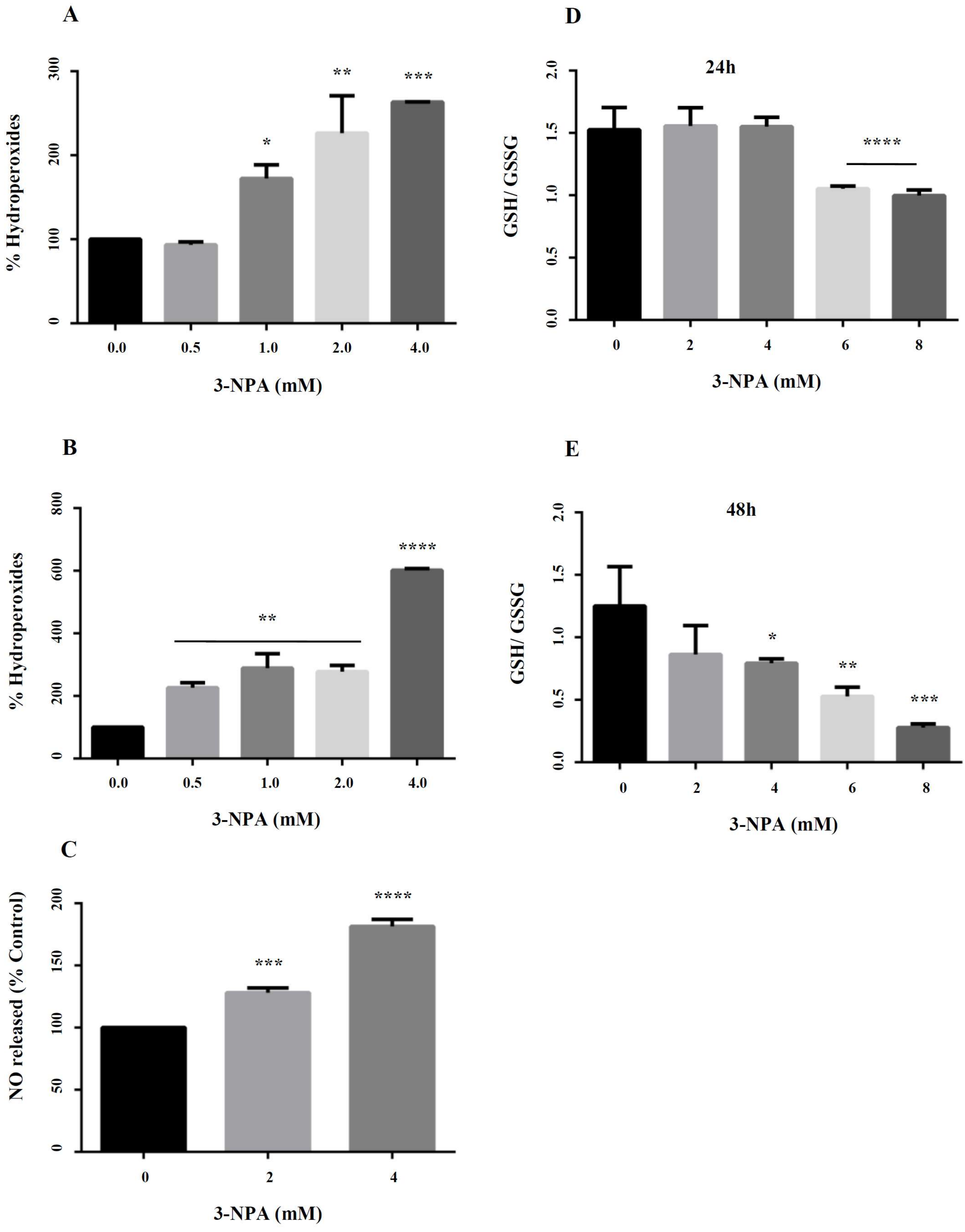

Supplementary figure 5

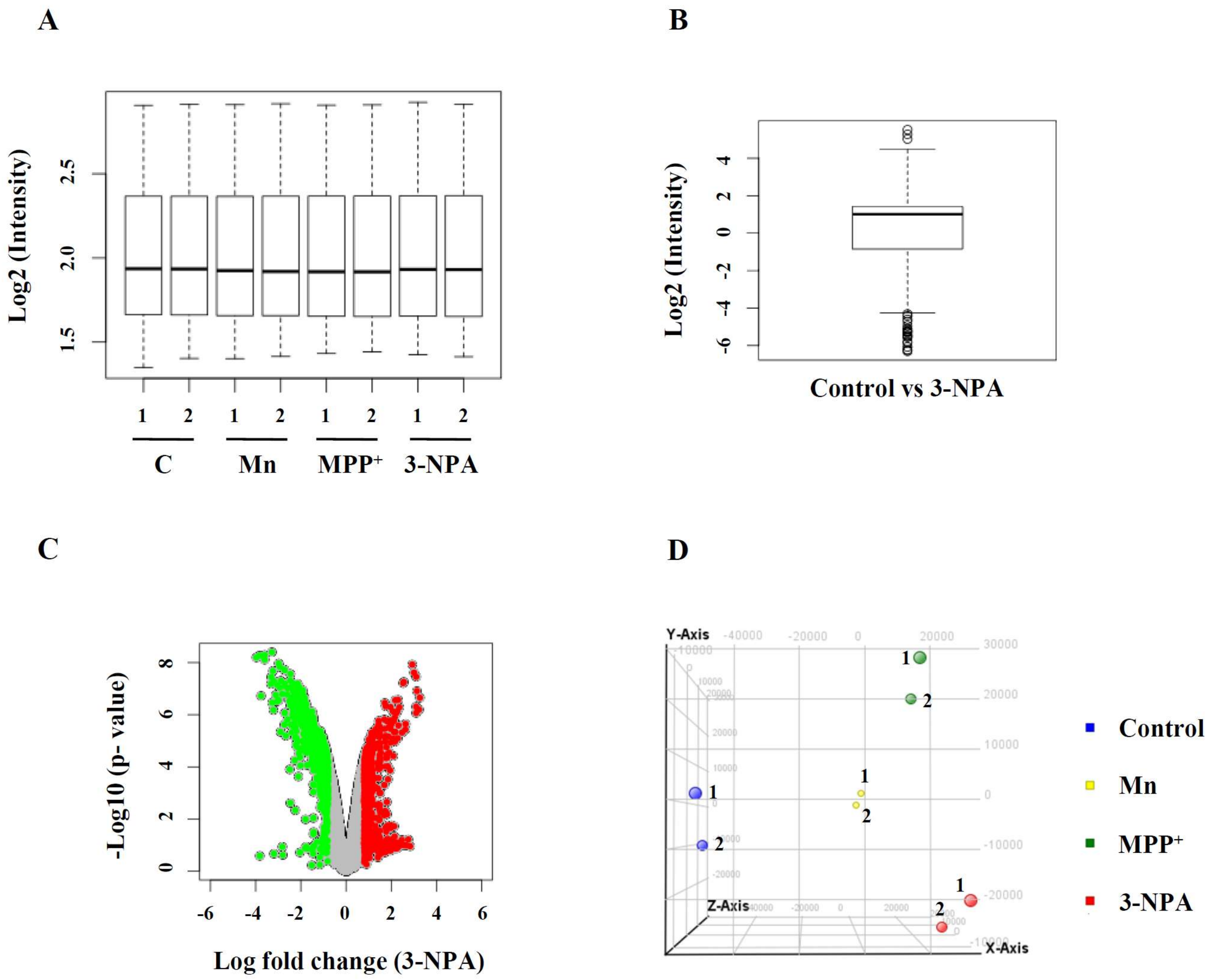

Supplementary figure 6

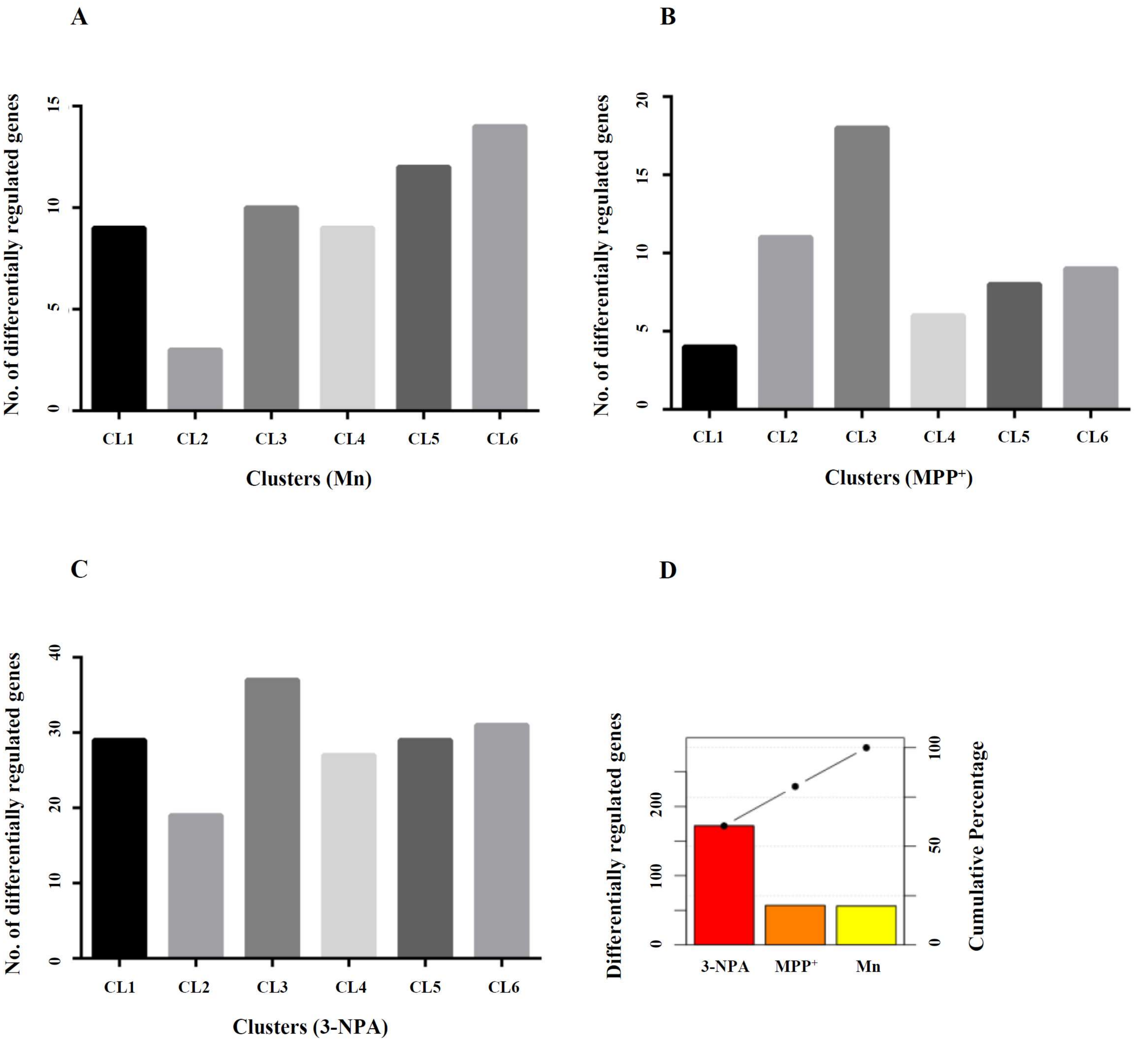

Supplementary figure 7

A

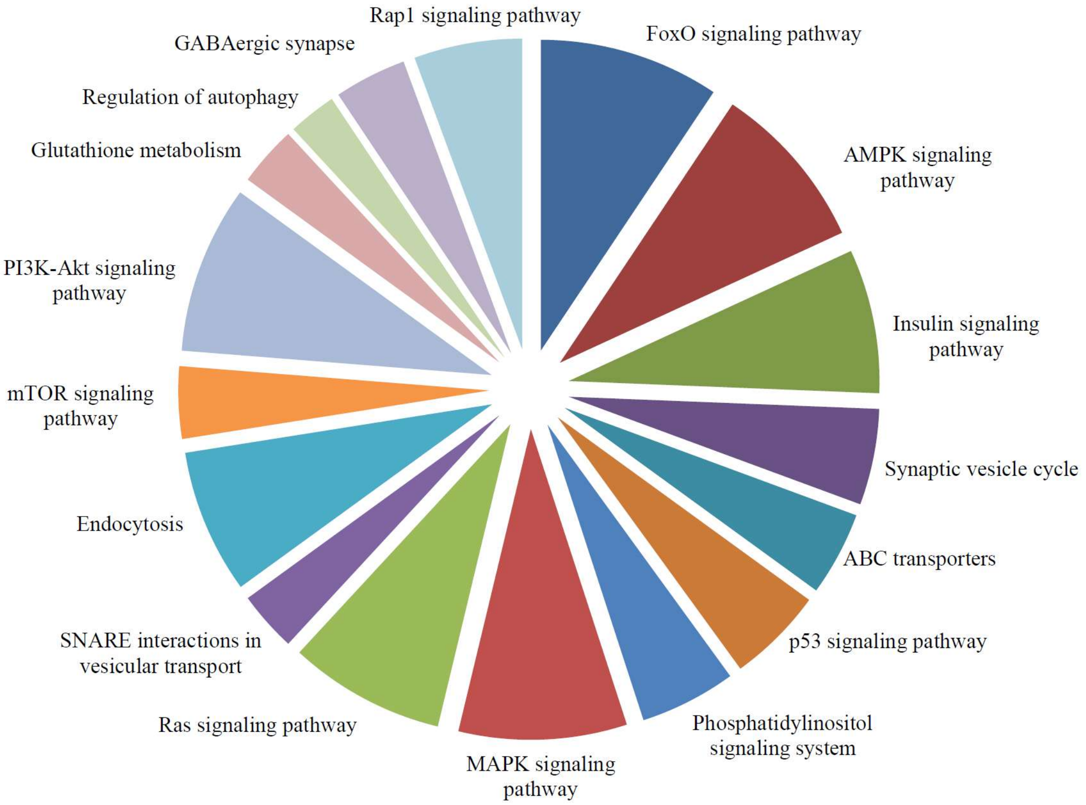

B

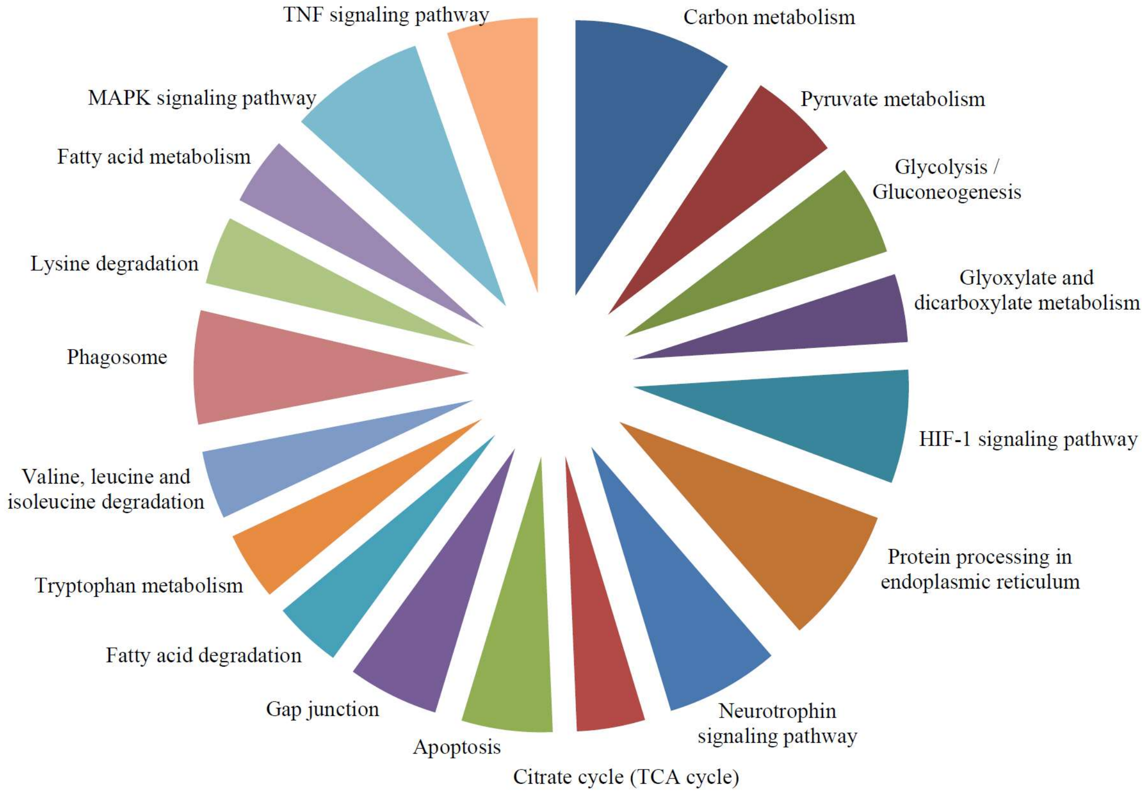

Supplementary figure 8

A

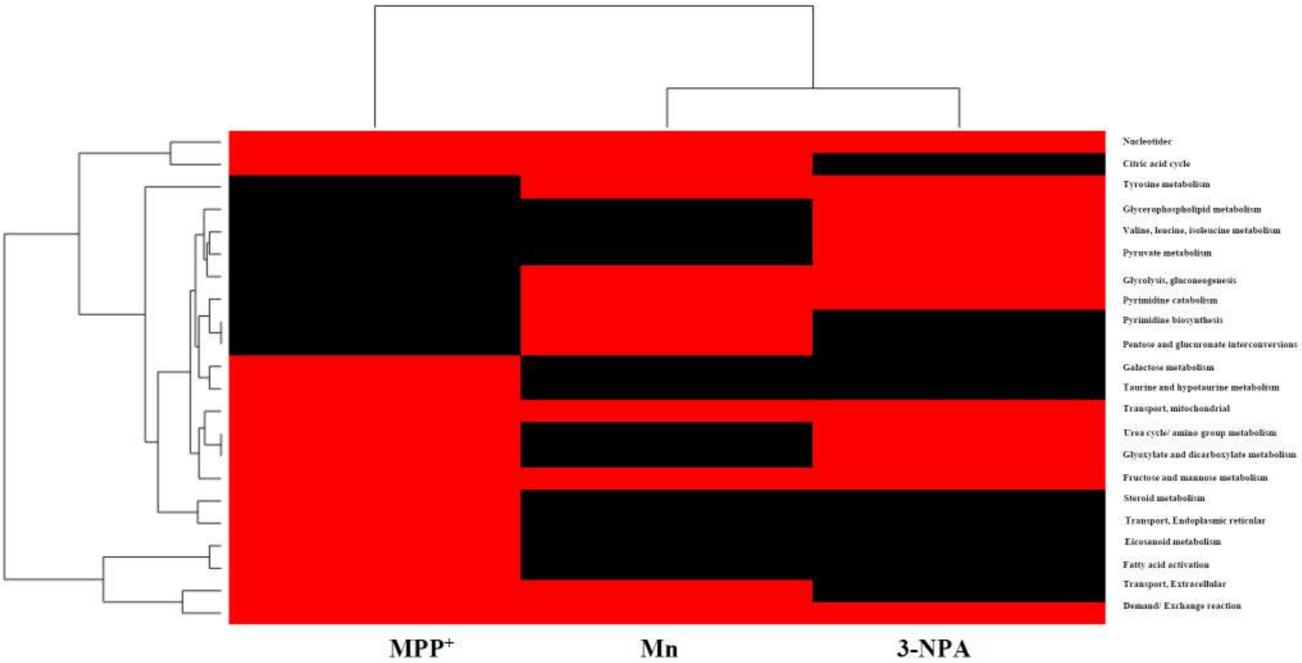

B

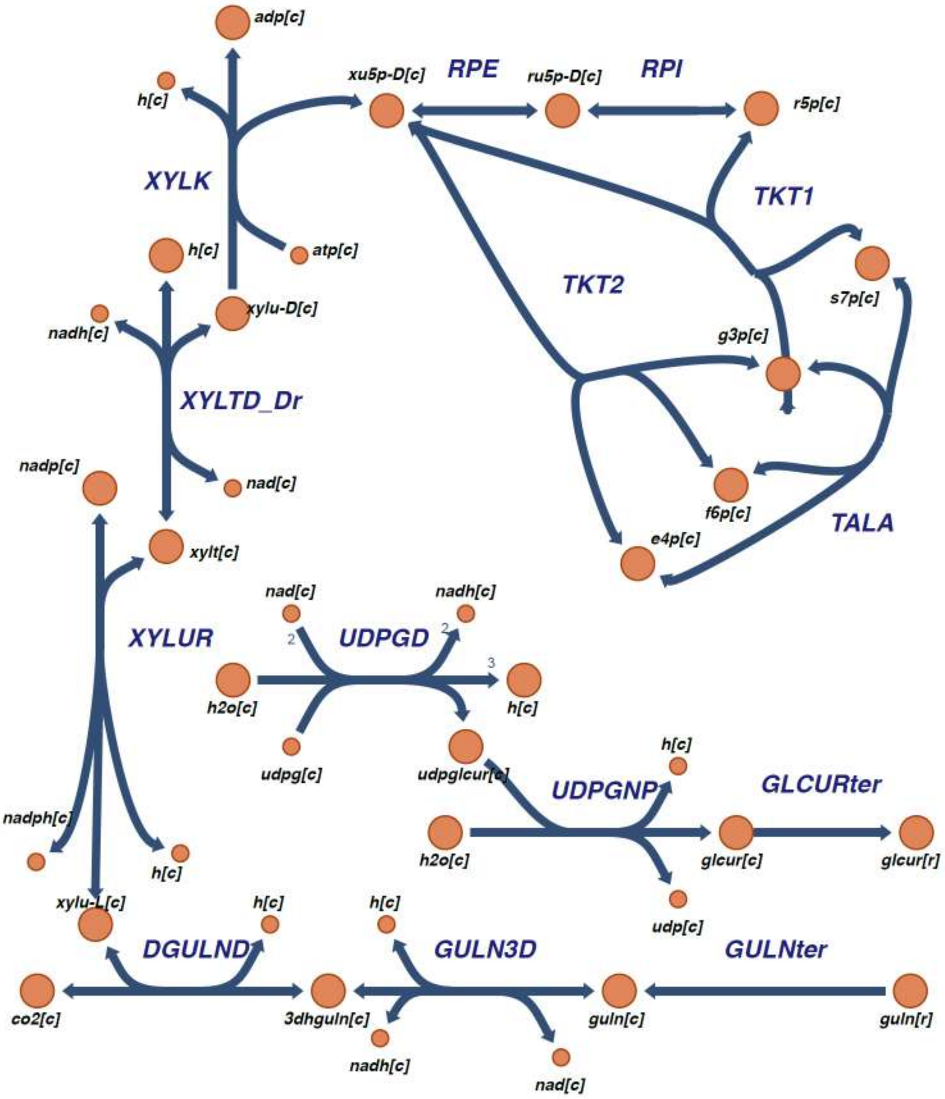

Supplementary figure 9

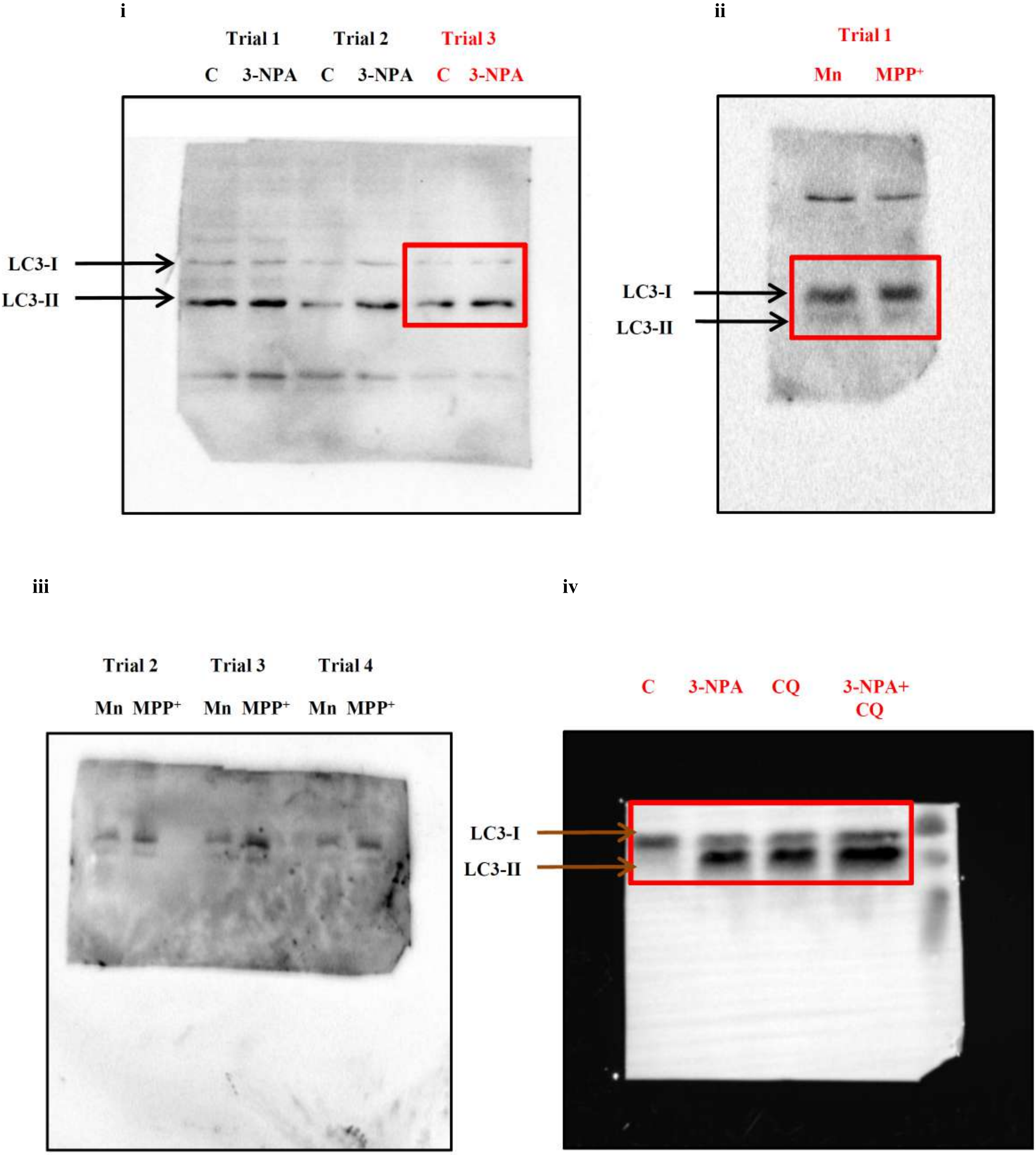

Supplementary figure 10

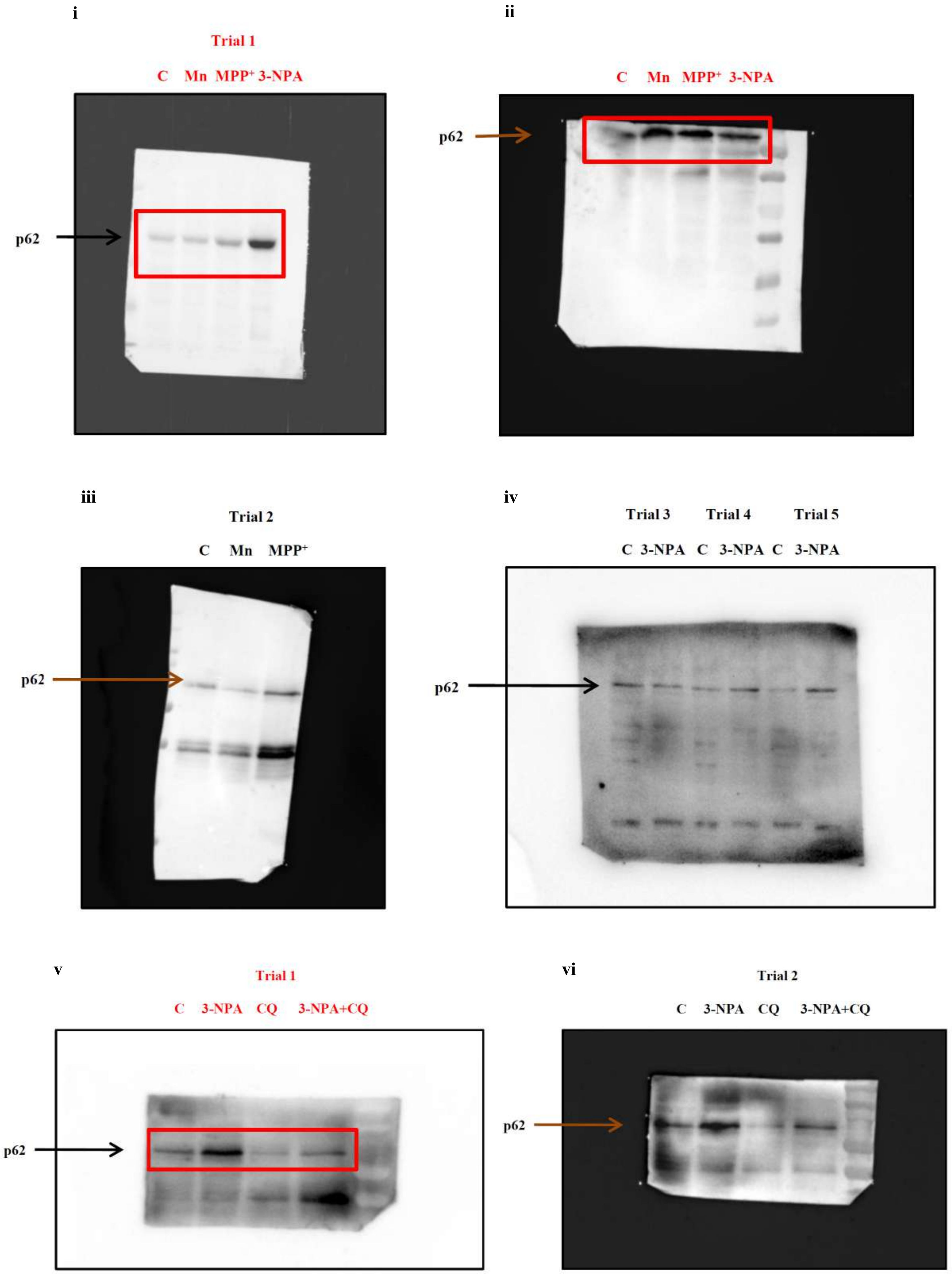

Supplementary figure 11

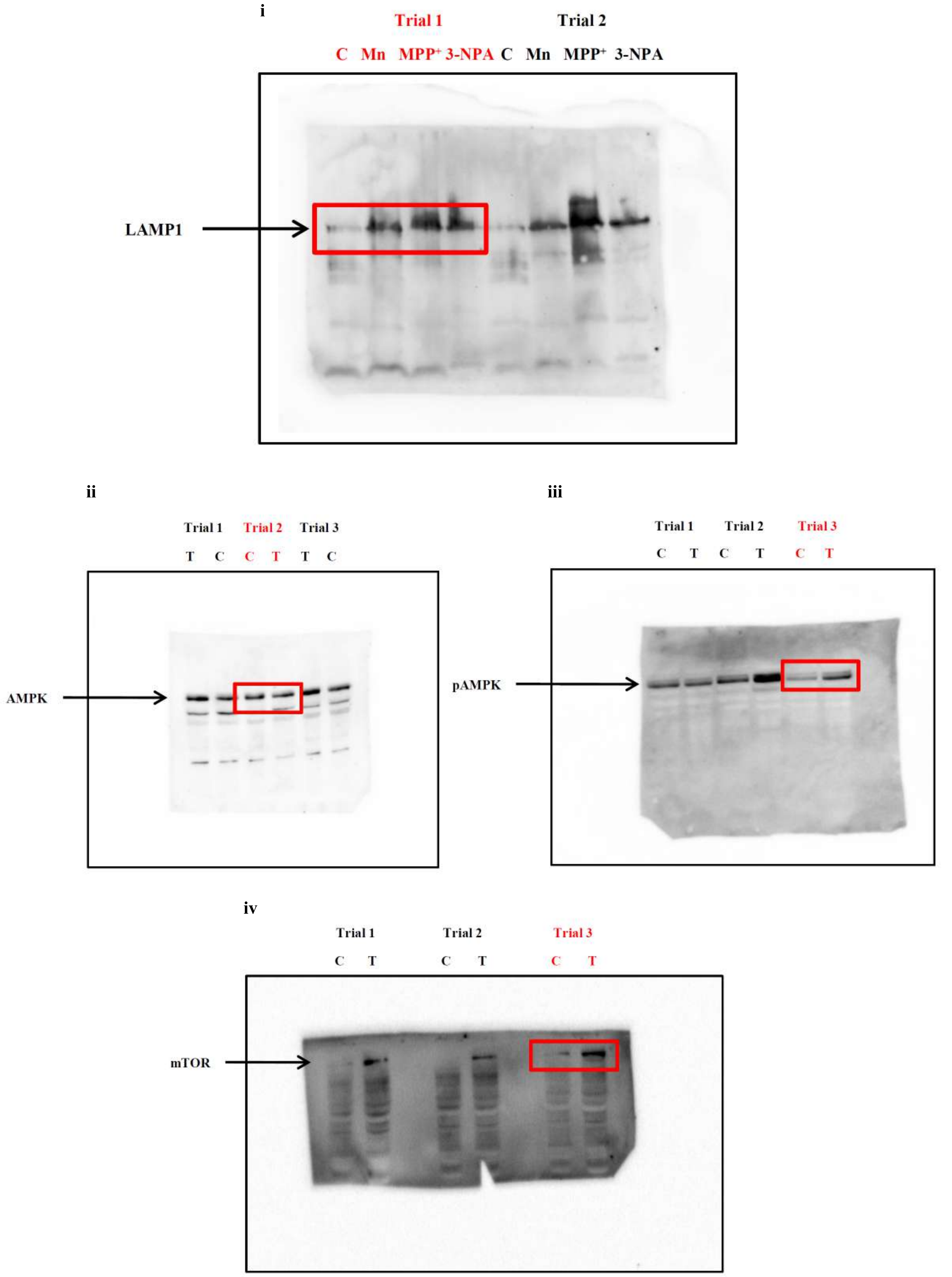

Supplementary figure 12

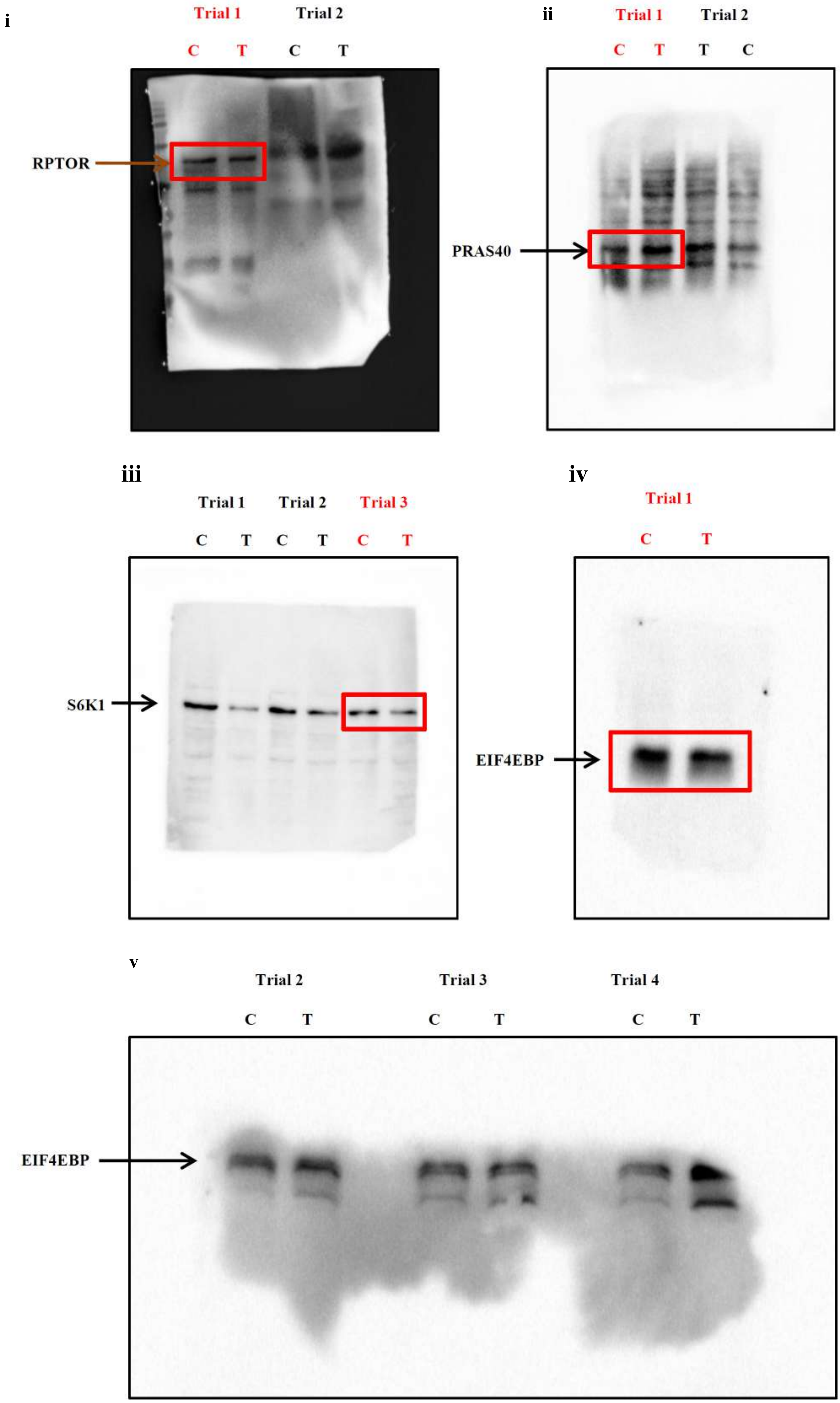

Supplementary figure 13

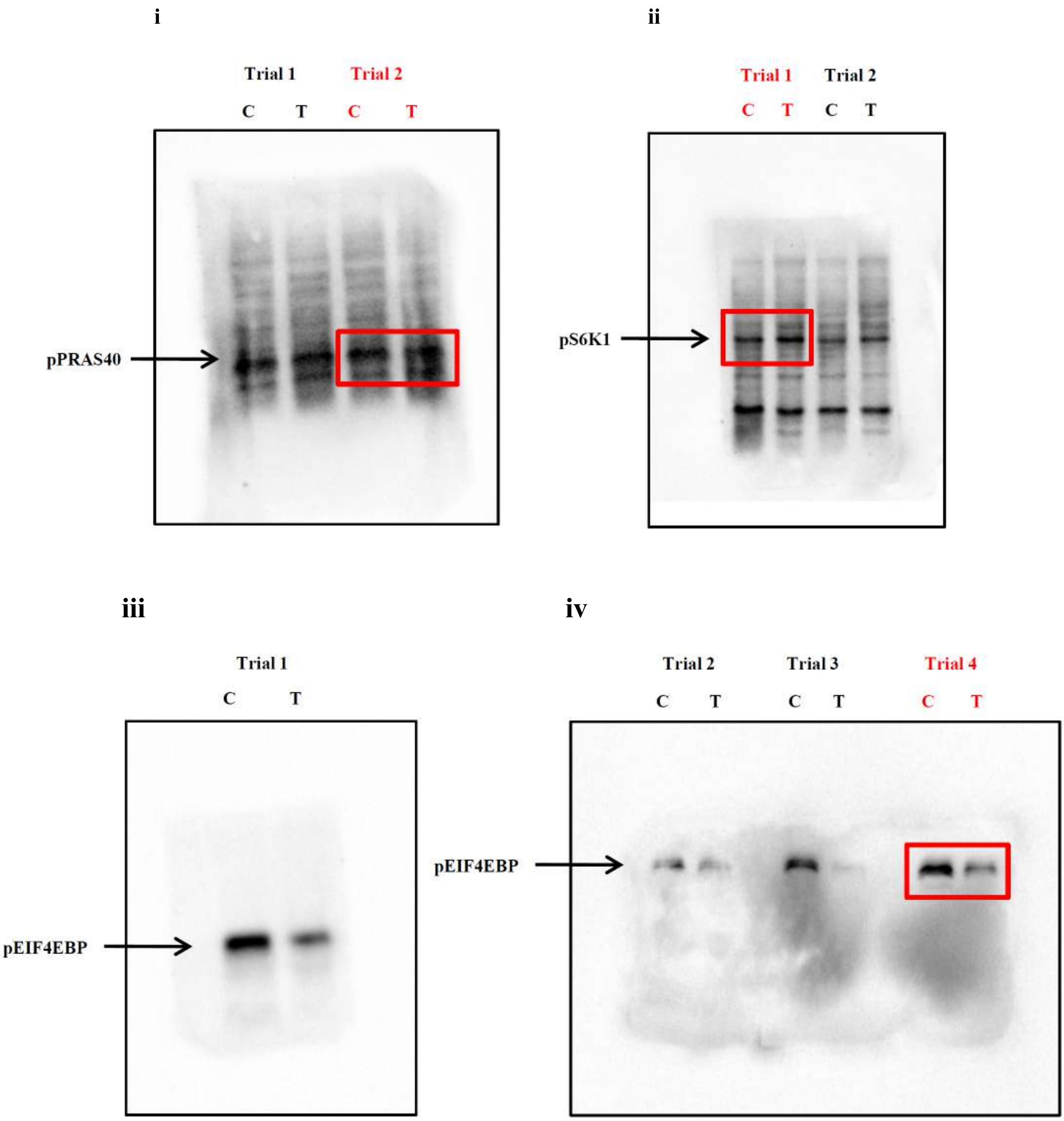

Supplementary figure 14

i

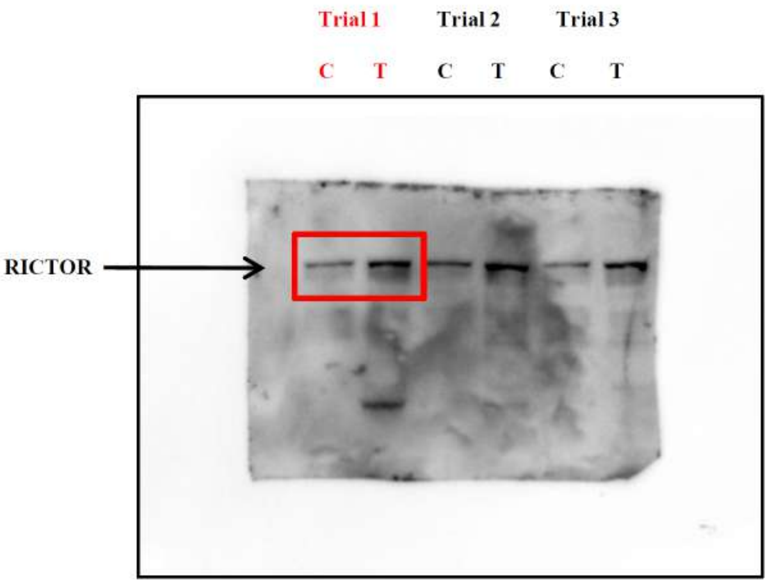

ii

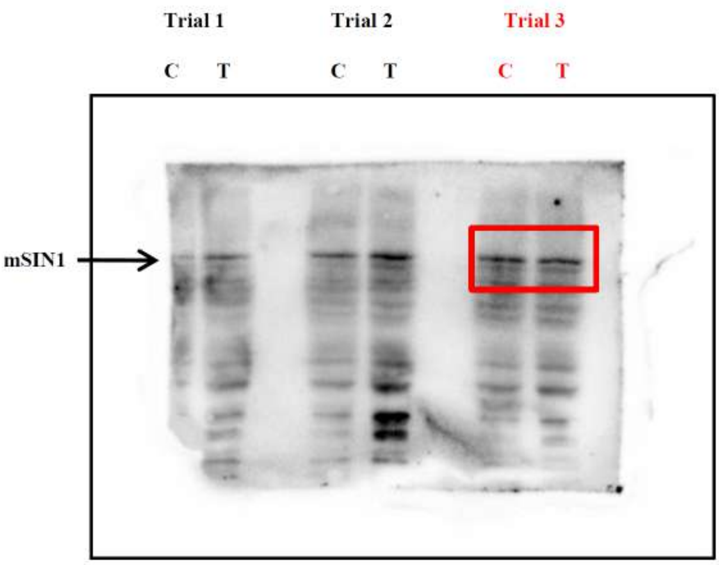

iii

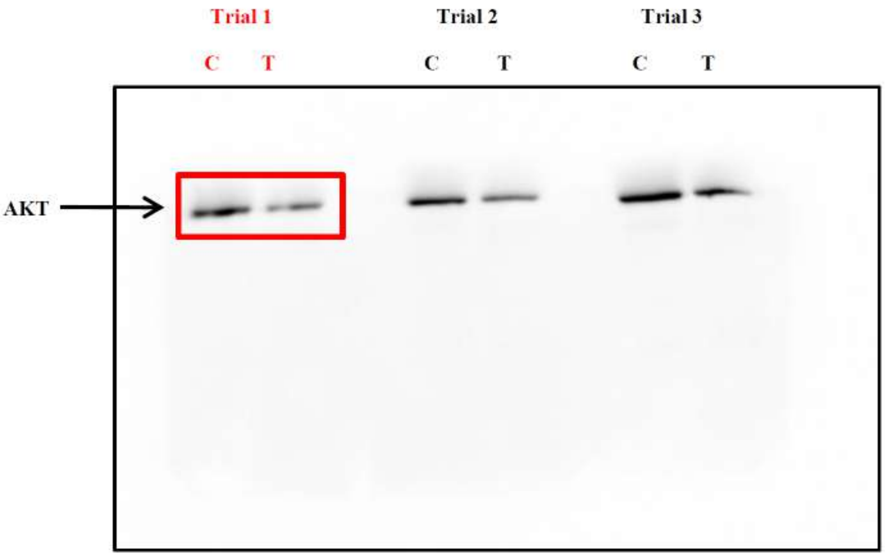

Supplementary figure 15

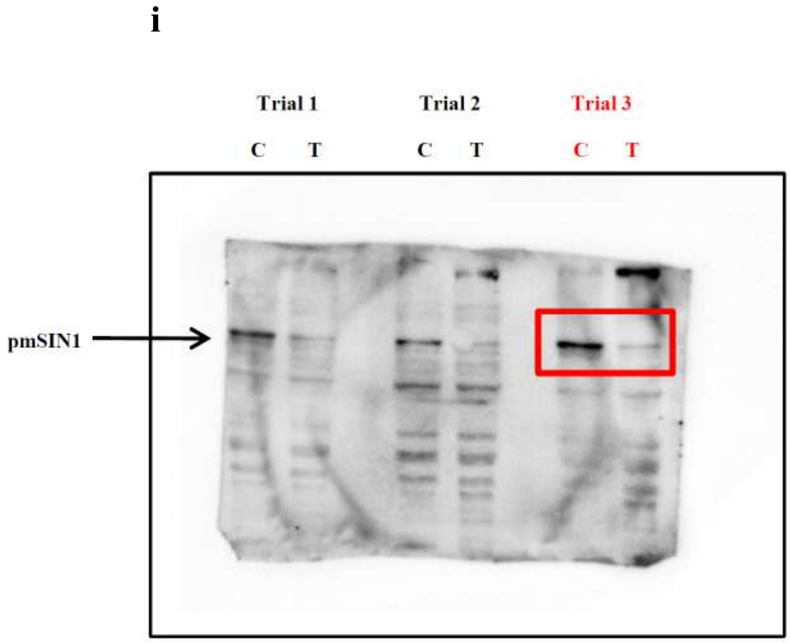

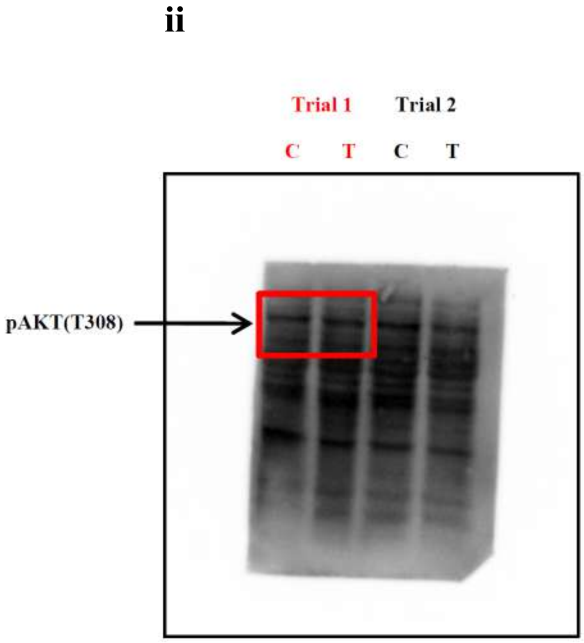

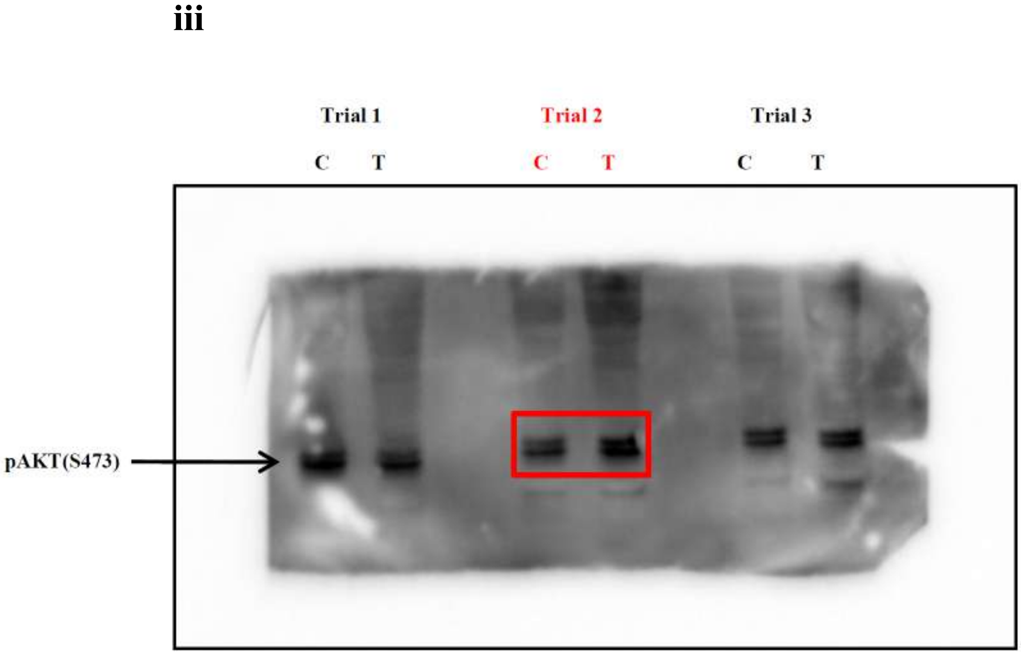

Supplementary figure 16

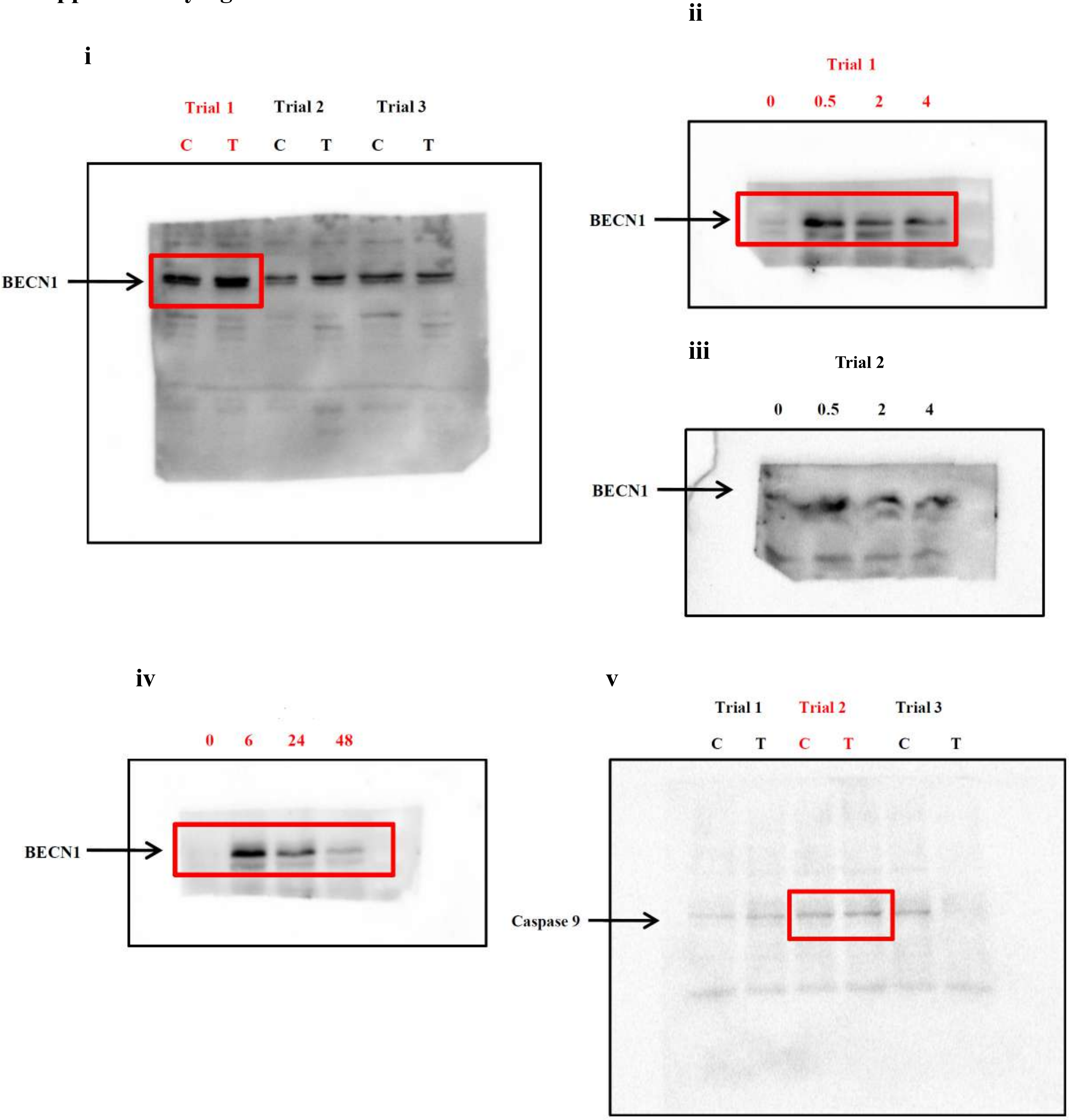

Supplementary figure 17

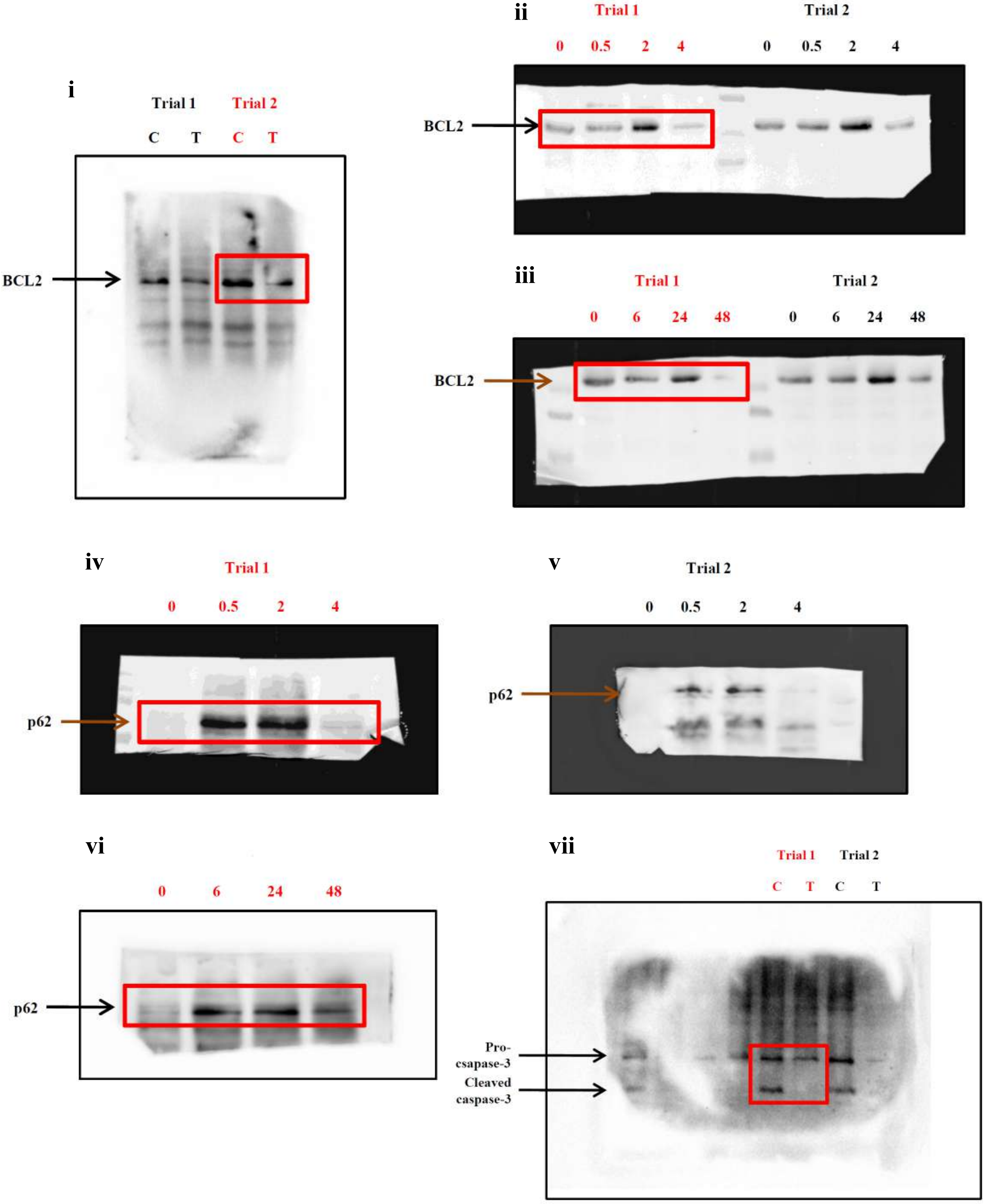

Supplementary figure 18

i

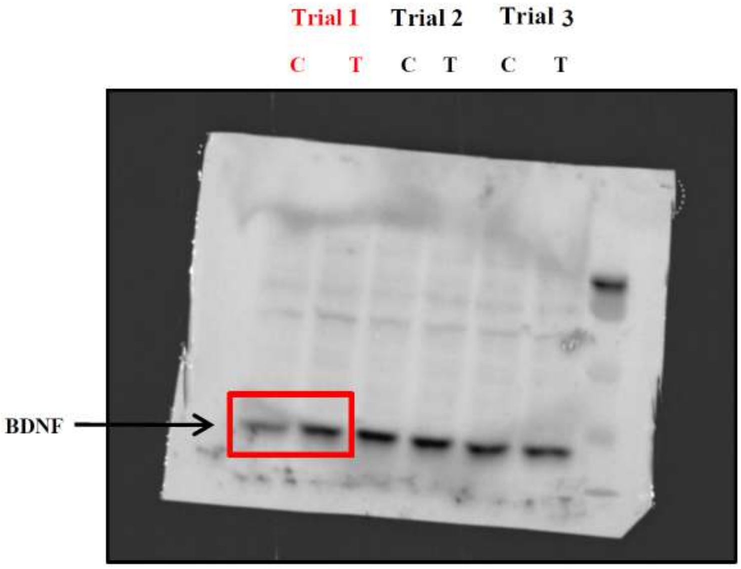

ii

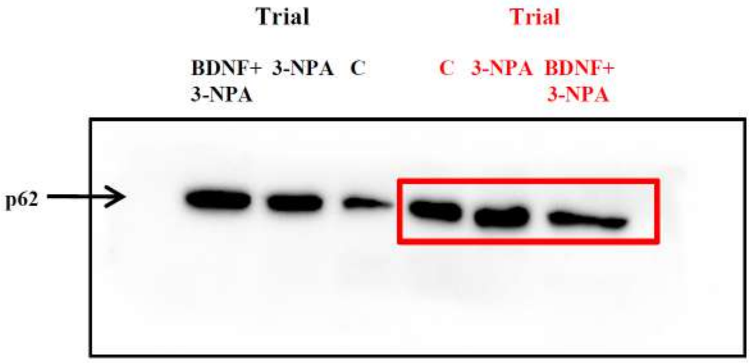

iii

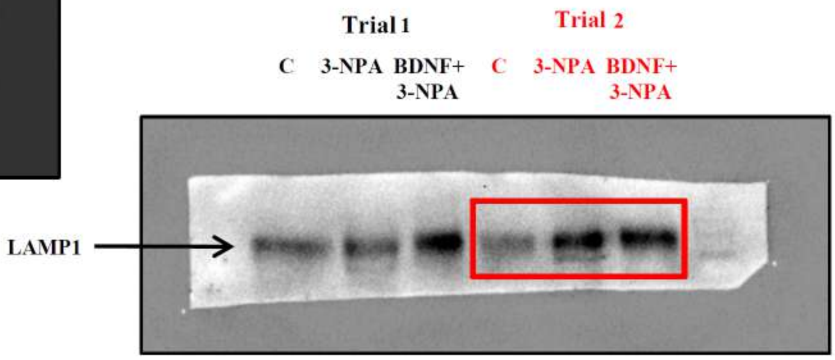

iv

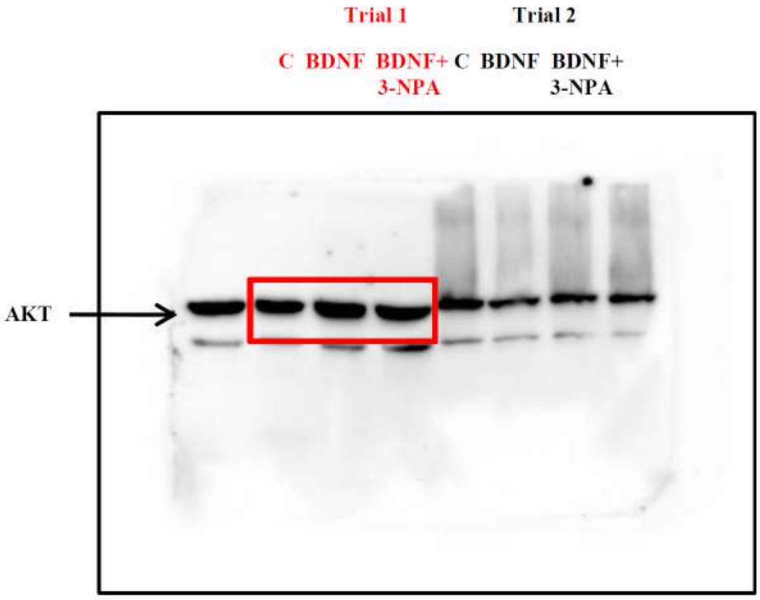

v

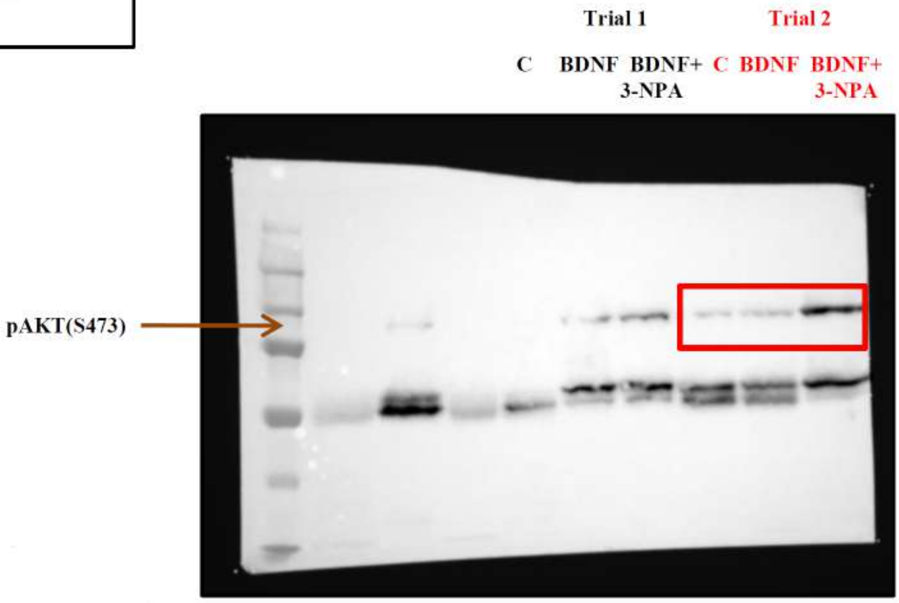

Supplementary figure 19

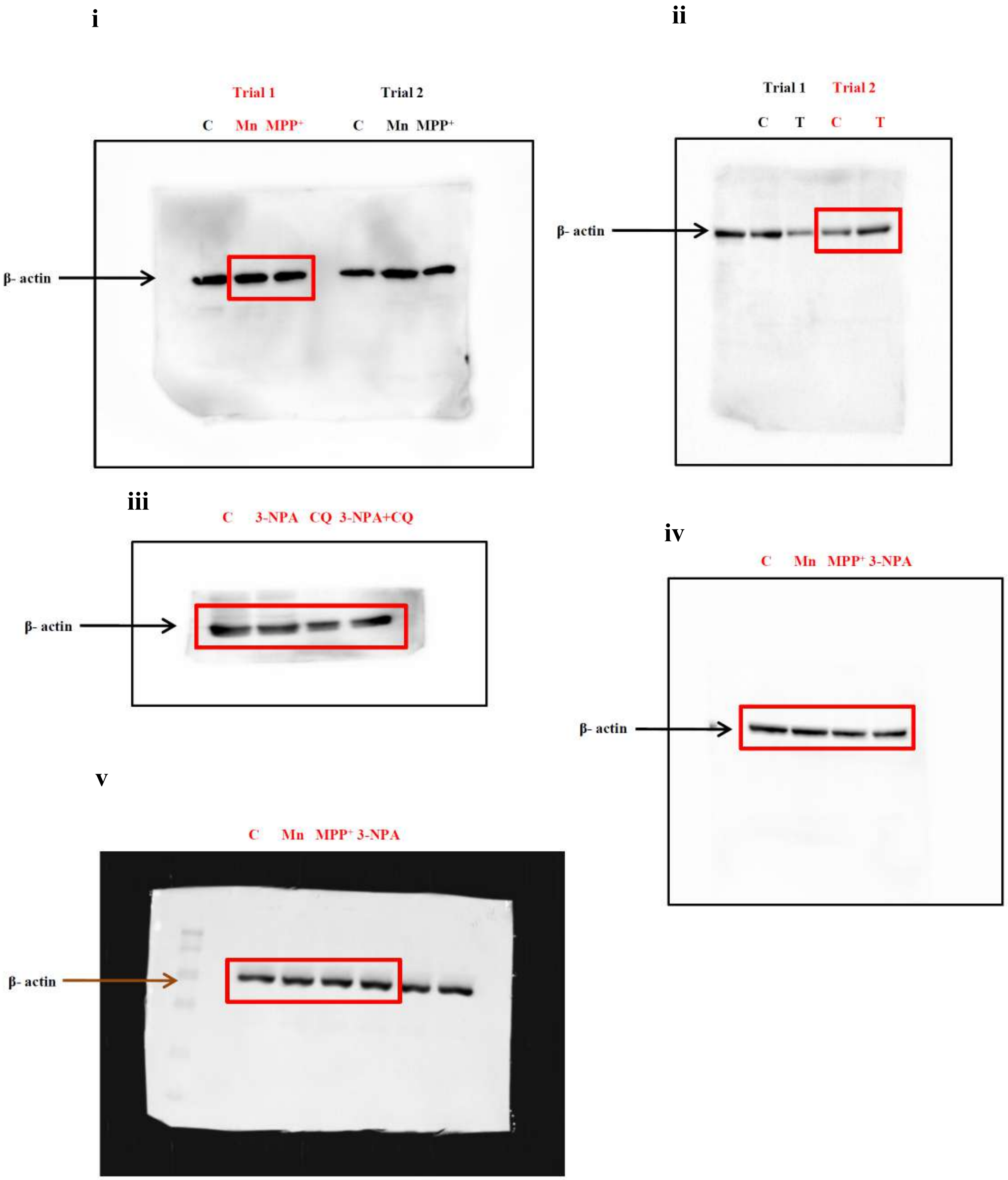

vi

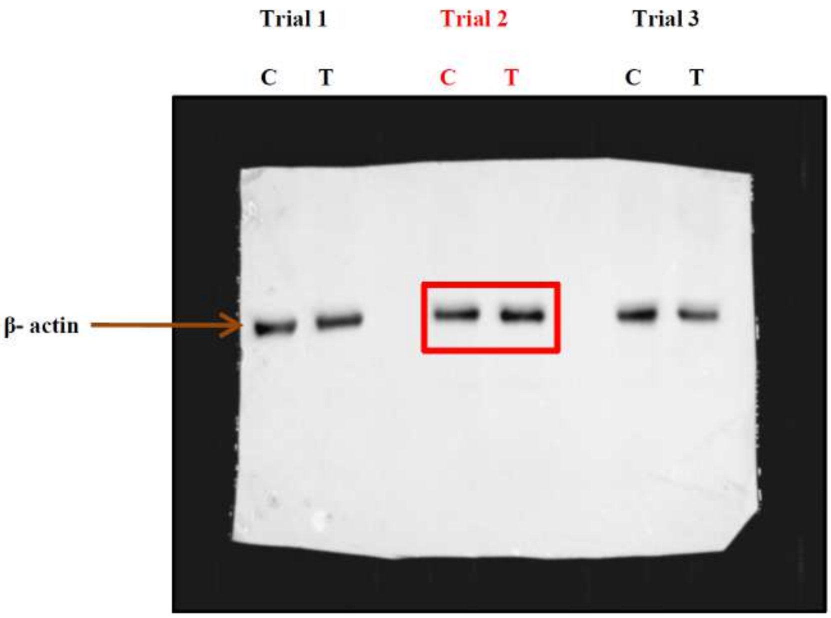

vii

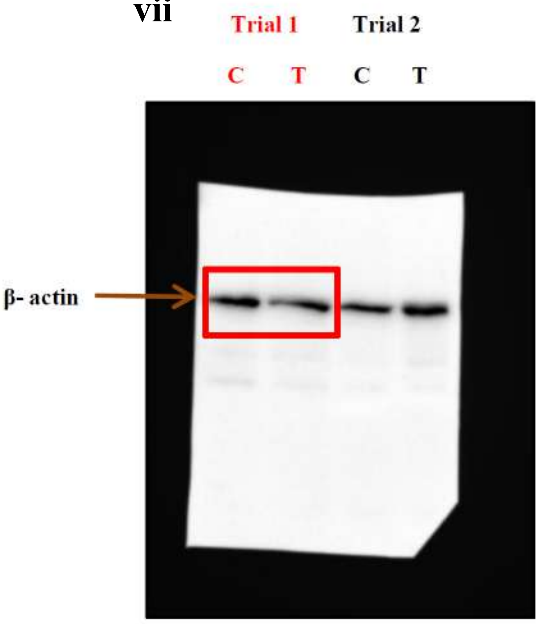

viii

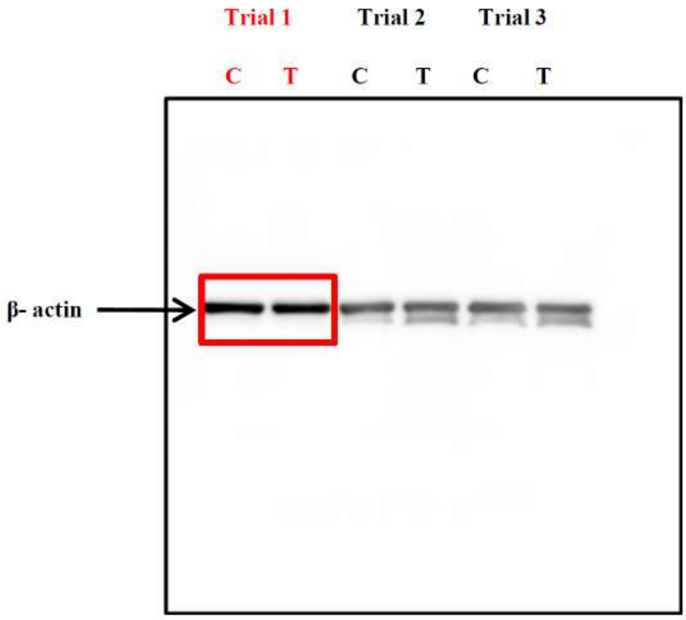

ix

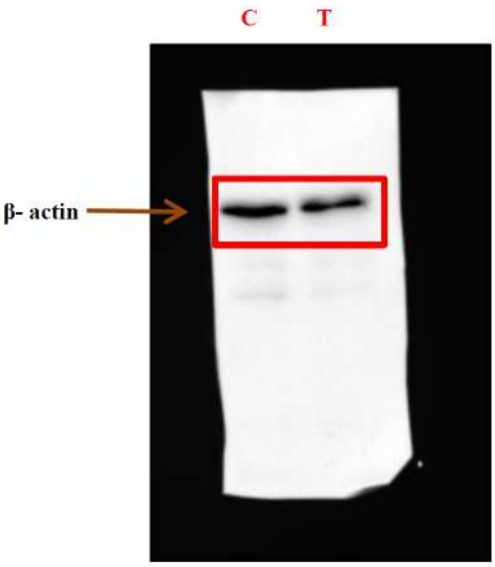

x

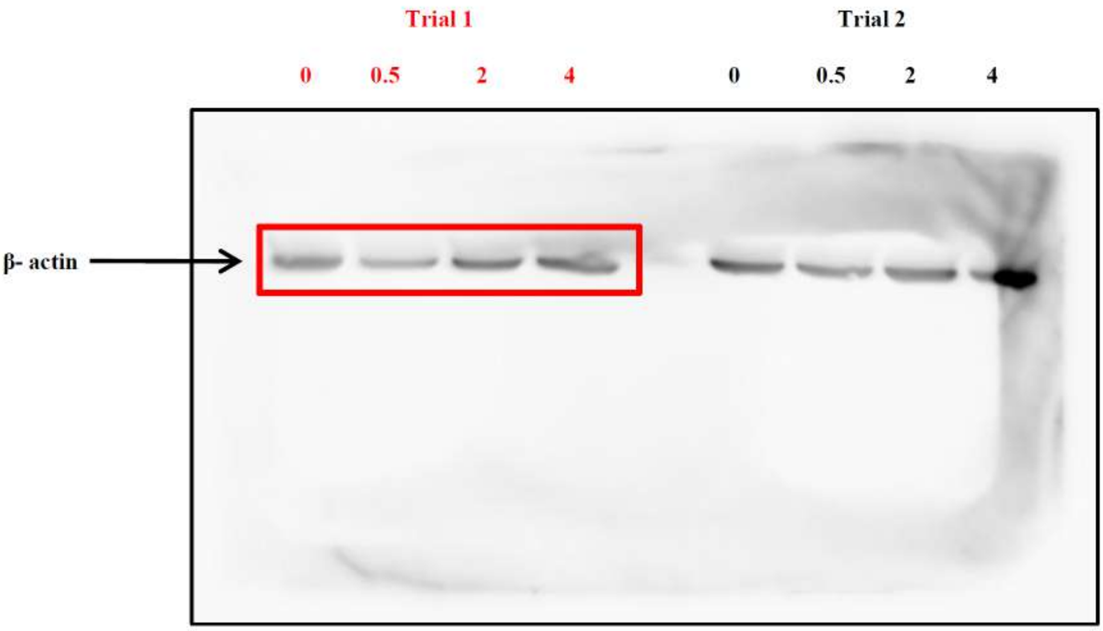

xi

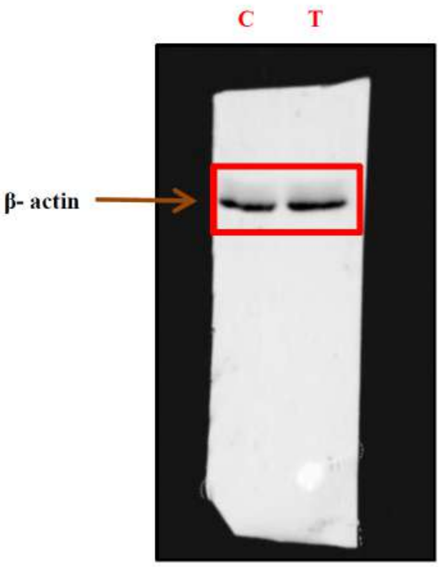

xii

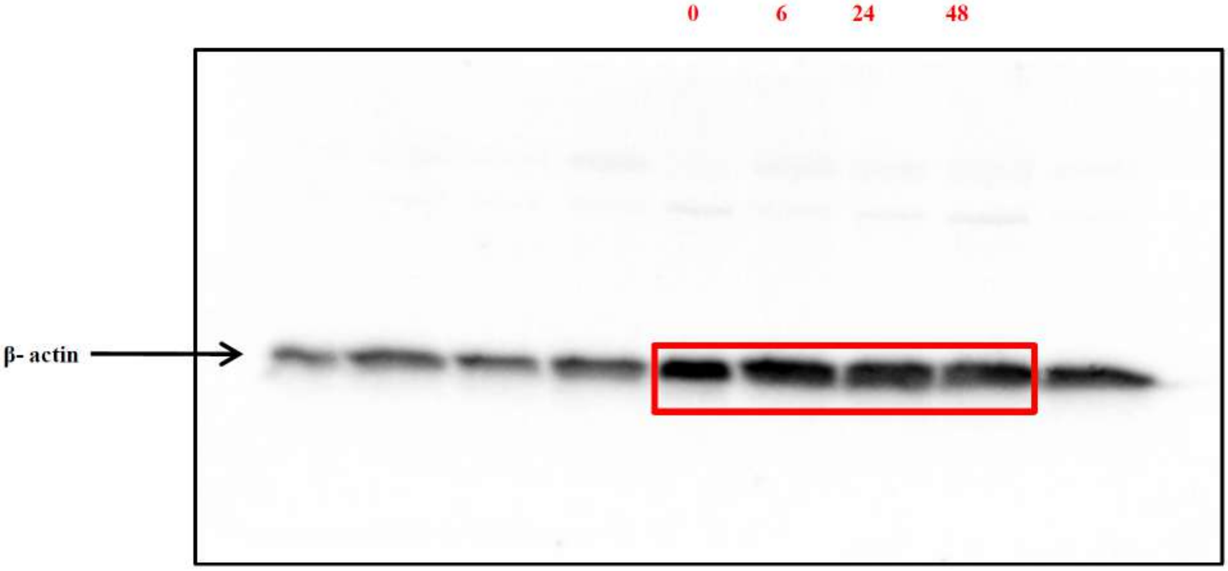

xiii

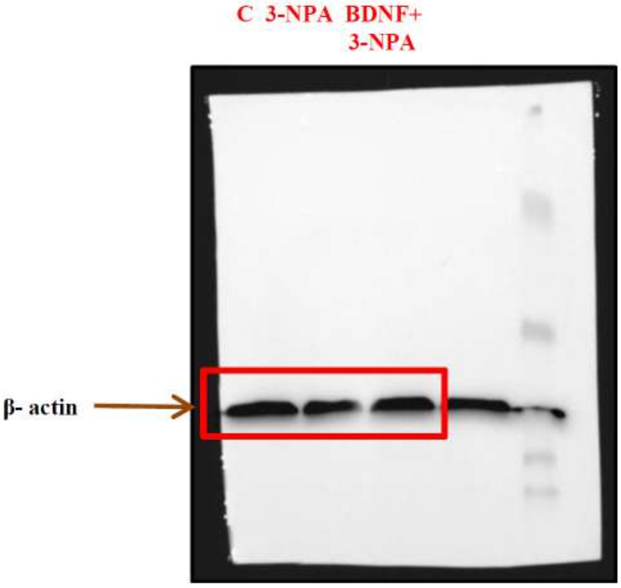

xiv

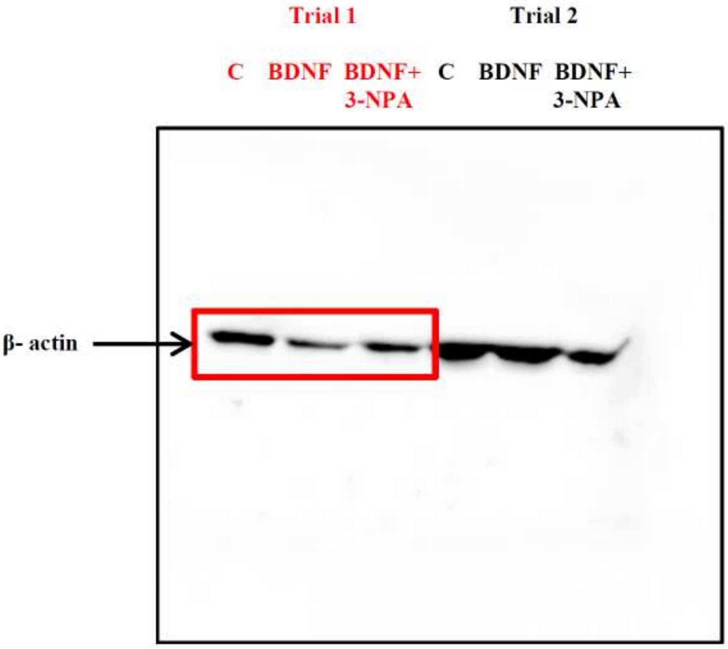

Supplement: Supplementary file 1 — Supplementary Information 1. [file 41598_2020_79339_MOESM1_ESM.pdf]
